# Total Syntheses of the Proposed Structure of Iriomoteolide-1a, -1b and Synthesis of Three Derivatives for Structural Studies

**DOI:** 10.3390/md20100587

**Published:** 2022-09-20

**Authors:** Arun K. Ghosh, Hao Yuan

**Affiliations:** 1 Department of Chemistry, Department of Medicinal Chemistry, Purdue University, West Lafayette, IN 47907, USA; 2 Department of Chemistry, Purdue University, West Lafayette, IN 47907, USA

**Keywords:** iriomoteolide-1a, total synthesis, macrolides, cytotoxicity, ene reaction, diastereomers

## Abstract

Iriomoteolide-1a and iriomoteolide-1b are very potent cytotoxic agents, isolated from marine dinoflagellates. We carried out the enantioselective syntheses of the proposed structures of these natural products. However, our analysis of the NMR spectra of the synthetic iriomoteolide-1a and the natural products revealed that the structures of iriomoteolide-1a and iriomoteolide-1b were assigned incorrectly. Based upon our detailed analysis of the spectral data of the synthetic iriomoteolide-1a and the natural products, we rationally designed three diastereomers of the proposed structure of **1** in an effort to assign the correct structures. The key steps of our syntheses of the proposed structures of iriomoteolides involved a highly diastereoselective ene reaction, a carbocupration that utilized a Gilman reagent, a Julia–Kocienski olefination to couple fragments, and Yamaguchi macrolactonization to form the target macrolactone. This synthetic route was then utilized to carry out syntheses of three diastereomers to the proposed structure of **1**. These diastereomeric structures show close similarities to natural iriomoteolide-1a; however, there were differences in their spectral data. While natural iriomoteolides exhibited potent cytotoxicies, our preliminary biological evaluation of synthetic iriomoteolide-1a, iriomoteolide-1b, and all three synthetic derivatives did not show any appreciable cytotoxic properties.

## 1. Introduction

**Figure 1:**
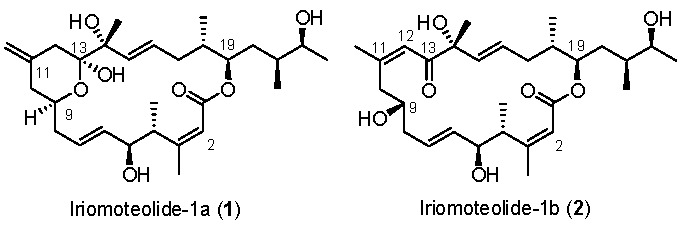
Proposed structures of iriomoteolide-1a (**1**) and -1b (**2**).

Marine natural products are a great source of structurally intriguing bioactive molecules with novel modes of action [1,2]. The field of marine natural products is immensely important in modern drug discovery. Already, many new approved drugs with interesting biological mechanisms are in pharmacies [3,4]. The field has great potential in modern medicine; however, it is vastly unexplored. The synthesis of these bioactive molecules and exploration of structure activity relationship studies are playing an important role in drug discovery today [5,6]. Iriomoteolide-1a (**1**) (Figure 1) is a 20-memembered cytotoxic macrolide, which was isolated by Tsuda and co-workers from a benthic HYA024 strain of dinoflagellate *Amphidinium* sp. collected off Iriomote Island, Japan in 2007 [7,8]. It displayed very potent cytotoxicity against human B lymphocyte DG-75 cells, with an IC_50_ value of 2 ng/mL. Furthermore, it exhibited cytotoxicity against Epstein–Barr virus (EBV)-infected human lymphocyte Raji cells (IC_50_ = 3 ng/mL) [7,8]. The initial structure of iriomoteolide-1a (**1**) was determined based on extensive 2D-NMR studies and mass spectroscopic analyses. The relative and absolute configurations were assigned based on the NMR studies, conformational analyses of derivatives of **1** with Mosher’s reagent. Later, Tsuda et al. reported the isolation of iriomoteolide-1b (**2**) [8], which was isolated from the same HY A024 strain of dinoflagellate *Amphidinium* sp. Iriomoteolide-1b (**2**) is structurally related to iriomoteolide-1a (**1**). Instead of a 6-membered hemiketal ring at the C9–C13 position and an exo-methylene group at C11 in iriomoteolide-1a (**1**), iriomoteolide-1b (**2**) possesses a ketone at C13 conjugated with a *Z*-double bond at C11–C12 and a hydroxyl group at C9. Treatment of iromoteolide-1a (**1**) with triethylamine in dichloromethane for 168 h furnished a polar product. The ^1^H NMR analyses reveal that the product is identical to iriomoteolide-1b (**2**). However, the IC_50_ value of **2** against DG-75 cells is found to be less potent than that of **1** (IC_50_ 900 ng/mL) [7,8]. Iriomoteolides targets have attracted considerable synthetic interest, leading to the syntheses of various segments of iriomoteolides [9,10,11,12,13,14,15,16,17]. In addition, synthesis of the proposed structures of iriomoteolides, as well as syntheses of structural variants of iriomoteolides, have been reported [18,19,20,21,22,23]. Thus far, neither the biological mechanism of action nor the correct structures of iriomotelides have been reported. Herein, we report our revised syntheses of the proposed structures of iriomoteolide-1a and -1b. Our convergent and highly stereoselective synthetic route was utilized for the syntheses of three rationally designed structural variants for structural elucidation and biological studies. 

## 2. Results and Discussion

### 2.1. Synthetic Plan 

**Figure 2:**
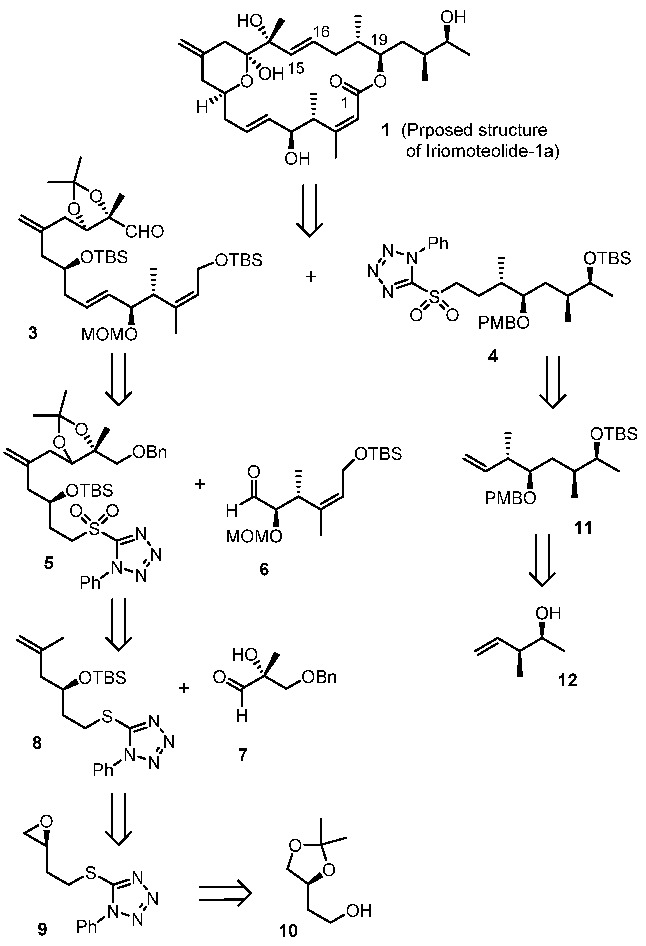
Retrosynthetic approach to iriomoteolide-1a (**1**).

Our retrosynthetic analysis is outlined in Figure 2. Our convergent synthetic strategy involves a Julia–Kocienski olefination [24,25] of aldehyde **3** and sulfone **4**. The resulting *trans*-olefin intermediate was converted to iriomoteolide macrolatone, using Yamaguchi macrolactonization [26] as the key step to build the 20-membered macrolactone. The synthesis of C1–C15 fragment **3** relied upon another Julia–Kocienski olefination from sulfone **5** and aldehyde **6**. An ene reaction of aldehyde **7** and olefin **8** was designed to furnish Sulfone **5**. A Cu(I)-mediated epoxide ring opening reaction provides the olefin **8** from expoxide **9**, which was readily prepared from the known alcohol **10**. The synthesis of C16–C23 segment **4** was planned from alkene **11** by hydroboration-oxidation, followed by conversion of the resulting alcohol to sulfone derivative **4**. Alkene **11** was be synthesized using an asymmetric crotylboration of the aldehyde derived from **12** as the key step. Optically active alcohol **12** can be conveniently obtained from asymmetric crotylboration of acetaldehyde.

### 2.2. Synthesis of C7–C15 Fragment **5**

**Scheme 1:**
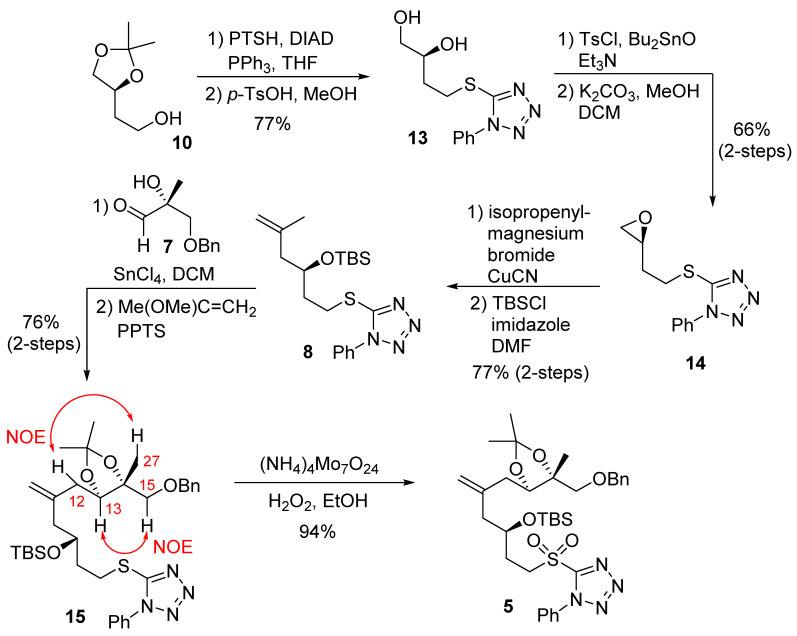
Synthesis of C7–15 fragment **5**.

The synthesis of C7–C15 fragment **5** was planned by using a diastereoselective ene reaction [27,28], as outlined in Scheme 1. Treatment of the known alcohol **10** with 1-phenyl-1H-tetrazole-5-thiol under Mitsunobu’s condition [29] afforded the corresponding sulfide. Deprotection of the acetonide group gave the diol **13**. Tosylation of diol **13** in the presence of triethylamine and dibutyltin oxide, followed by treatment of potassium carbonate in a mixture of methanol and dichloromethane, furnished the epoxide **14**. Reaction of epoxide **14** with isopropenylmagnesium bromide in the presence of a catalytic amount of copper(I) cyanide resulted in the corresponding alcohol. Interestingly, Curran and co-workers reported that the reaction of the enantiomer of **14** with an alkynyllithium reagent furnished a by-product that resulted from the displacement of 1-phenyltetrazole [30]. The alcohol from **14** was then protected with TBSCl to afford the silyl ether **8**. A SnCl_4_-mediated ene reaction of aldehyde **7** and olefin **8** was carried out to provide the corresponding alcohol in 76% yield as a mixture of diastereomers with good selectivity (8:1 dr). The use of TiCl_4_ as a Lewis acid led to the desired product but with a lower yield (40%). A chelation-controlled addition between the *α*–hydroxyl group and the aldehyde resulted in good diastereoselectivity (8:1 dr) for the ene reaction [31,32]. The diol was protected as an acetonide derivative to afford **15**. The absolute configuration of the new chiral center at C13 was identified as drawn in **15** (*R*-configuration) using ^1^H-NMR NOESY experiments. NOESY between H13 and H15, and NOESY between H12 and H27 were observed, as shown in acetonide **15**.

While this new chiral center would be removed in the late stage of the synthesis via oxidation to the corresponding ketone, good diastereoselectivity in the ene reaction simplified the NMR spectra. Sulfide **15** was then oxidized by ammonium molybdate and hydrogen peroxide to afford the sulfone **5** in good yield.

#### 2.2.1. Syntheses of C16–C23 Segment **4** and C1–C6 Fragment **6**

**Scheme 2:**
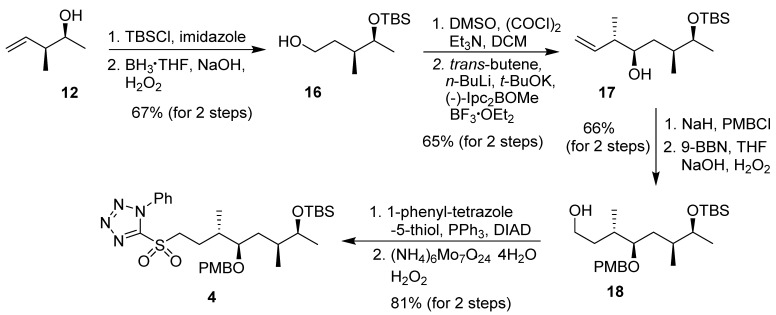
Synthesis of C16–C23 fragment **4**.

The synthesis of C16–C23 segment **4** was carried out, as shown in Scheme 2. Asymmetric crotylboration of acetaldehyde using cis-2-butene and (+)-B-methoxy-diisopinocamphenylborane using the protocol developed by Brown and co-workers [33,34] furnished optically active syn-alcohol **12**. As reported previously [19], alcohol functionality was protected as a TBS-ether and hydroboration-oxidation of the alkene under standard condition afforded alcohol **16** in good yield. Swern oxidation of **16** provided the aldehyde, which was subjected to Brown’s asymmetric crotylboration using (−)-B-methoxy-diisopinocamphenylborane and trans-2-butene to afford alcohol **17** in good yield and with excellent diastereoselectivity (10:1). Alcohol **17** was protected as a PMB-ether and hydroboration-oxidation of the olefin furnished alcohol **18**. Syntheses of derivatives of **17** and **18** with different protecting groups have been reported [17]. This was converted to sulfone **4** by the Mitsunobu reaction, followed by oxidation of the sulfide to sulfone.

**Scheme 3:**
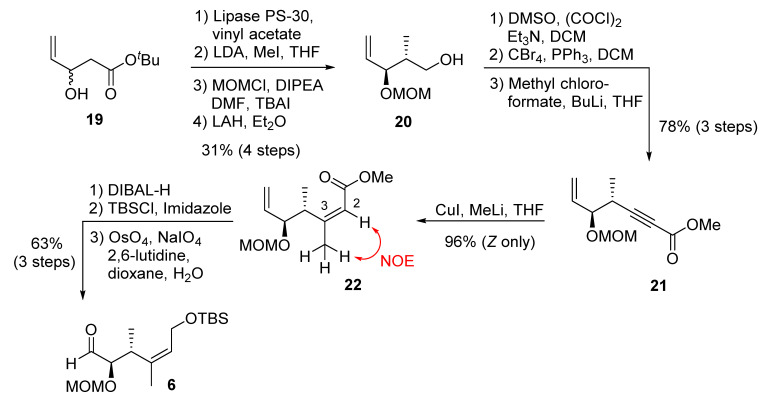
Synthesis of C1–C6 fragment **6**.

The synthesis of C1–C6 fragment **6** is shown in Scheme 3. The racemic alcohol **19**, obtained from the aldol reaction of *tert*-butylacetate and acrolein, was subjected to immobilized lipase PS-30 catalyzed kinetic resolution in pentane in the presence of excess vinyl acetate, at 30 °C for 19 h, to provide enantio-enriched (*R*)-**19** in 98% ee*,* along with the corresponding enantiomeric acetate derivative [35]. Treatment of (*R*)-**19** with lithium diisopropylamide, followed by the reaction of the resulting dianion with methyl iodide as described by previously [36], afforded the corresponding anti-alcohol as a single isomer by ^1^H NMR analysis. The resulting alcohol was protected as a MOM-ether. Reduction of the resulting ester with LAH furnished alcohol **20**. Synthesis of derivative of **20** with different protecting groups was reported [17]. Swern oxidation, followed by Corey–Fuchs’ homologation [37] of the aldehyde, provided the corresponding dibromo olefin. Treatment of the dibromide with butyllithium, followed by reaction of the resulting alkynyl anion with methyl chloroformate, furnished alkynyl ester **21** in excellent yield. A carbocupration of alkynyl ester **21** was carried out with freshly prepared Gilman reagent [38] at −40 °C to provide *Z*-olefin **22** as a single product in excellent isolated yield. The observed NOE between the protons at C2 and Me at C3 is consistent with the assigned *Z* geometry in ester **22**. DIBAL-H reduction of **22,** followed by protection of the resulting alcohol with TBSCl, furnished the corresponding silyl ether. Selective oxidative cleavage of the terminal olefin provided the C1–C6 fragment **6**.

**Scheme 4:**
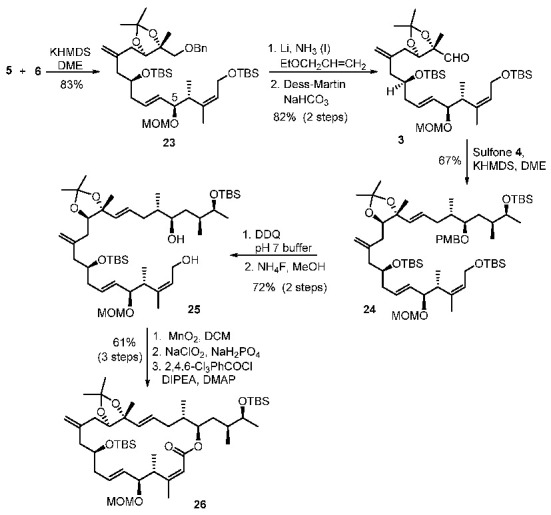
Synthesis of macrolactone intermediate **26**.

**Scheme 5:**
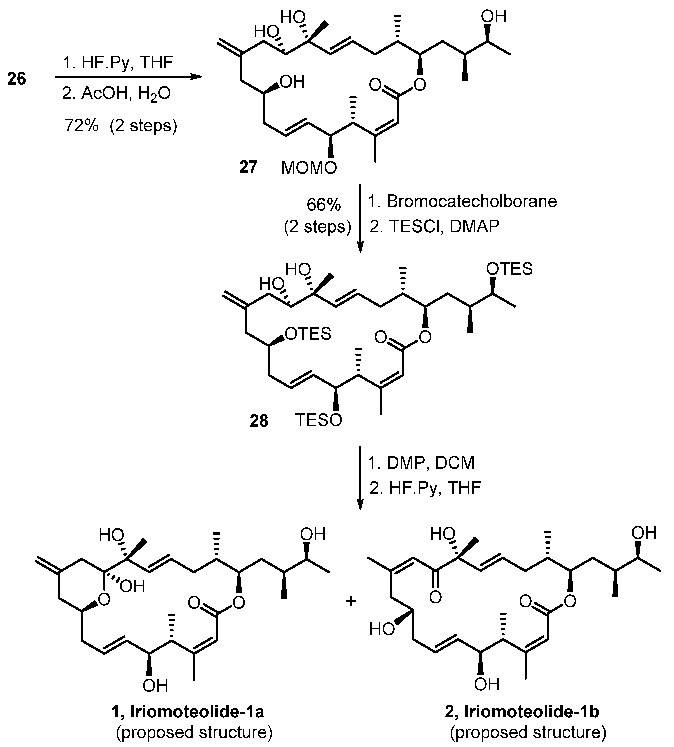
Synthesis of proposed structures of iriomoteolide-1a and -1b.

With sulfone **5** and aldehyde **6** in hand, total syntheses of the proposed structures of iriomoteolide-1a and 1b were successfully achieved. The synthesis featured two successive Julia–Kocienski olefinations. As shown in Scheme 4, the first Julia–Kocienski reaction between sulfone **5** and aldehyde **6** afforded *trans*-olefin **23** in excellent yield. Removal of the benzyl ether, followed by DMP oxidation [39,40] of the resulting alcohol, afforded aldehyde **3**. A second Julia–Kocienski reaction of aldehyde **3** and sulfone **4** furnished *E*-olefin **24** as the only isolated product in good yield. Removal of PMB ether, followed by selective removal of the primary TBS-ether with NH_4_F, resulted in allylic alcohol **25**. Oxidation of **25** with MnO_2_ followed by NaClO_2_ afforded the corresponding carboxylic acid [41]. Yamaguchi macrolactonization furnished macrolactone **26** in good yield. Macrolactone **26** was converted to the proposed structures of irimoteolide-1a and irimoteolide-1b, as shown in Scheme 5. Treatment of **26** with HF•Py followed by aqueous AcOH resulted in tetraol derivative **27**. Bromocatecholborane promoted the removal of the MOM group and furnished the corresponding pentaol derivative. Treatment of the free alcohols with TESCl and DMAP selectively provided TES- ether derivative **28** in good yield. DMP oxidation [39,40] of the secondary alcohol provided the corresponding ketone and removal of the TES groups with exposure to HF•Py furnished iriomoteolide-1a (**1**) in 56% yield and iriomoteolide-1b (**2**) in 17% yield after silica gel chromatography. The ^1^H-NMR and ^13^C-NMR of synthetic iriomoteolide-1a and iriomoteolide-1b did not match with the reported data for these natural products [7,8].

**Figure 3:**
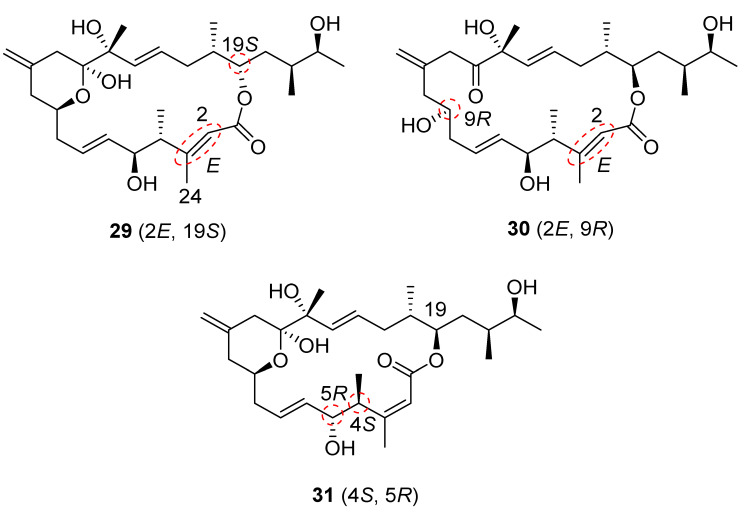
Structures of diastereomers **29**, **30** and **31**.

Although the ^1^H NMR and ^13^C NMR spectral data of our synthetic iriomoteolide-1a (**1**) are comparable to those of independent work reported from other groups [18,20], neither data of **1** nor data of **2** matched those of natural iriomoteolide-1a and -1b (Please see Supplementary Materials for NMR comparison). This suggested that the structures of both natural iriomoteolide-1a and iriomoteolide-1b have been assigned incorrectly. While there are many minor differences, the major discrepancies involve the ^1^H and ^13^C shifts at C4 (3.98 ppm and 40.6 ppm, respectively, for synthetic iriomoteolide-1a compared to 2.46 ppm and 47.9 ppm, respectively, for the natural product). In addition, there is a distinction of chemical shifts at C24 (1.96 and 20.8 ppm, respectively, for synthetic **1** compared to 2.12 and 23.8 ppm, respectively, for natural **1**). These discrepancies reveal that the α,β-unsaturated double bond configuration might be *E* instead of *Z*. In addition, the structure may be an epimer at the C4 and C5 positions. Based on the NMR analysis, three diastereomers, **29**, **30** and **31** (Figure 3), were designed. The syntheses of these structural variants were carried out utilizing our convergent synthetic route.

#### 2.2.2. Synthesis of Diastereomer **29**

**Scheme 6:**
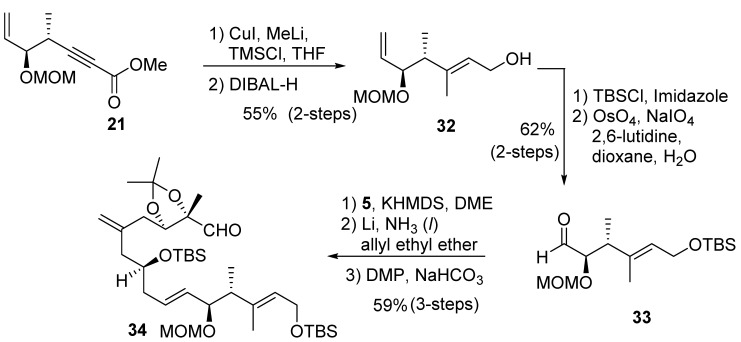
Synthesis of aldehyde **34**.

The synthesis of diastereomer **29** requires alteration of olefin geometry at C2–C3_._ As shown in Scheme 6, a carbocupration of alkynyl ester **21** with Gilman reagent at 0 °C in the presence of TMSCl resulted in the desired *E*-olefin as a major isomer (*E*:*Z* = 7:3) [38]. DIBAL-H reduction furnished the alcohol **32**, which was separated from its *Z*-isomer by flash column chromatography over silica gel. Protection of alcohol with *tert*-butyldimethylsilyl chloride and oxidative cleavage of the terminal olefin provided the C1–C6 fragment aldehyde **33** for diastereomer **29**. Treatment of sulfone **5** with slightly less than one equivalent of KHMDS, followed by exposure to aldehyde **33,** afforded the *E*-olefin. However, we found that the use of more than one equivalent base led to the epimerization of the chiral center on C5. Removal of the benzyl group followed by Dess–Martin oxidation [39,40] provided the C1–C15 fragment, aldehyde **34**.

**Scheme 7:**
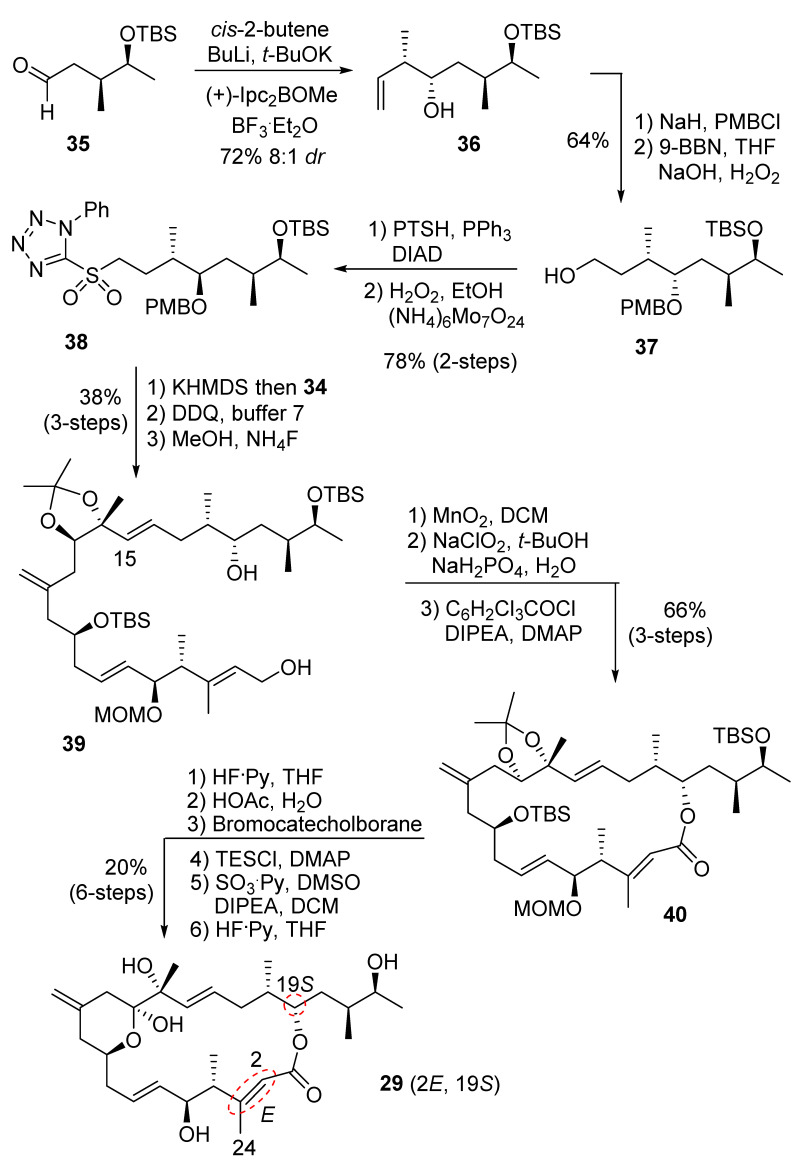
Synthesis of diastereomer **29**.

The synthesis of diastereomer **29** is shown in Scheme 7. Brown asymmetric crotyllation, by utilizing (+)-B-methoxydiisopino-campheylborane and *cis*-2-butene with aldehyde **35** derived from **16,** provided *syn*-alcohol **36** in good diastereoselectivity (8:1 dr). PMB protection and hydroboration-oxidation provided alcohol **37**. Alcohol **37** was converted to sulfone **38**, as described previously. A Julia–Kocienski olefination [24,25] between aldehyde **34** and sulfone **38** using KHMDS in 1,2-dimethoxyethane furnished (15*E*)-olefin in good selectivity (*E*:*Z* = 9:1). The use of THF as a solvent caused an increase in *Z*-olefin by-product (*E*:*Z* = 4:1). Removal of the PMB ether and primary TBS ether led to diol **39**. MnO_2_ oxidation and Pinnick oxidation [41] gave the corresponding seco-acid. Yamaguchi esterification furnished the macrolactone **40**. A protecting group exchange protocol [26] afforded the corresponding vicinal diol. A one-pot reaction with the addition of HF•Py and Parikh–Doering oxidation led to isomer **29** exclusively, without isomerization of the exomethylene group at C11.

#### 2.2.3. Synthesis of Diastereomer **30**

**Scheme 8:**
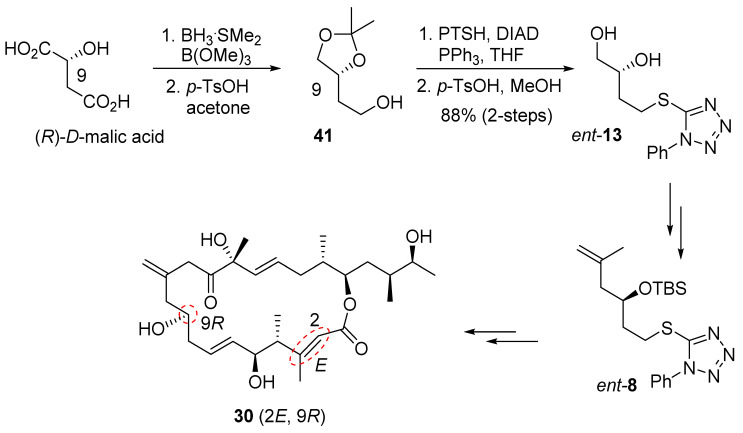
Synthesis of iriomoteolide diastereomer **30**.

The synthesis of diastereomer **30** requires alteration of the stereochemistry at C9. Our synthesis started with commercially available (*R*)-*D*-malic acid. As shown in Scheme 8, reduction of malic acid with the borane dimethylsulfide complex furnished the corresponding triol. Selective protection of the diol with acetone and *p*-TsOH furnished the alcohol **41** with desired chirality at C9. Using the previous synthetic route, enantiomeric sulfone *ent*-**8** was obtained. Sulfone *ent*-**8** was then converted to the corresponding macrolactone, as described previously. Protecting the group exchange protocol and oxidation, followed by the removal of the TES group as described previously, provided diastereomeric structure **30,** where the hydroxyl ketone stays as a *δ*-hydroxyl-ketone instead of cyclic hemiketal, as revealed from the analysis of its ^1^H NMR and ^13^C NMR spectral data.

#### 2.2.4. Synthesis of Diastereomer **31**

**Scheme 9:**
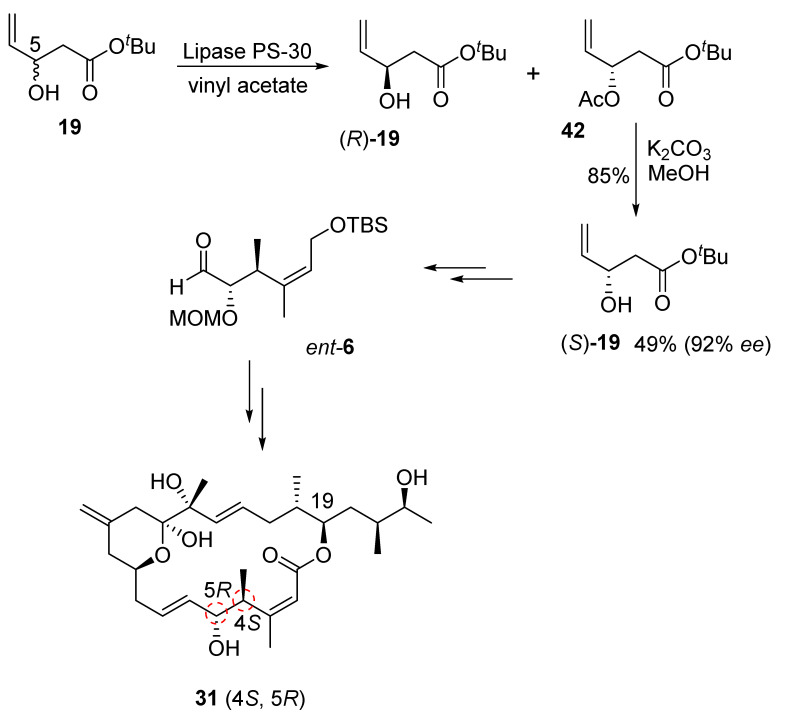
Synthesis of *ent*-**19** alcohol and iriomoteolide-1a diastereomer **31**.

Our synthesis of this diastereomeric structure of iriomoteolide-1a required altering the configurations at the C_4_ and C_5_ chiral centers. Therefore, the enantiomeric C1–C5 segment aldehyde, *ent*-**6,** was synthesized as shown in Scheme 9. Our enzymatic resolution of racemic alcohol **19** provided nearly a 1:1 mixture of (*R*)-**19** alcohol and its acetate derivative **42** in excellent yield. Saponification of acetate by treatment of K_2_CO_3_ in MeOH at −30 °C provided (*S*)-**19** alcohol in 92% *ee*. Seebach–Fráter alkylation [36] of (*S*)-**19** with methyl iodide, as described in Scheme 3, furnished the corresponding anti-alcohol. Protection of alcohol as an MOM ether, followed by the reduction of the ester using LAH, afforded *ent-***20** alcohol. This was then converted to C1–C6 segment aldehyde *ent*-**6**. Aldehyde *ent*-**6** was then exposed to Julia–Kocienski olefination with sulfone **5** to provide the corresponding *trans*-olefin, which was converted to the corresponding diastereomeric aldehyde, as described in Scheme 4. A second Julia–Kocienski olefination [24,25] with sulfone **4** provided the carbon framework for diastereomer **31**. This was converted to the macrolactone, followed by the final target diastereomer **31** by following the steps described in Scheme 5.

**Figure 4:**
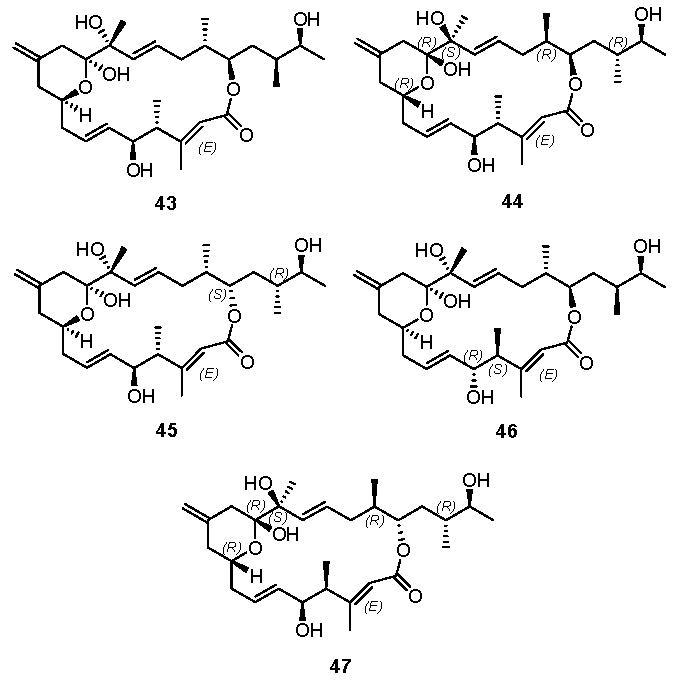
Structures of diastereomers **43, 44, 45, 46,** and **47**.

NMR spectra analysis of these diastereomers revealed that some individual chemical shifts, such as H4 and H24 of **29**, H19 and H26 of **31**, came closer to those of the natural product. These observations suggest that there may be an *E*-enoate and/or C4 and C5-epimers in the natural product. However, none of these isomers match the natural product. We carried out biological evaluations of the synthetic iriomoteolide-1a (**1**), -1b (**2**) and structural variants **29**, **30**, and **31**. However, none of these compounds show any appreciable cytotoxicity. Yang and Dai’s research groups also reported their independent synthetic approach of several other diastereomers, such as **43** [20,22], **44** [20], **45** [20], **46** [22] and **47** [20], as shown in Figure 4. Unfortunately, none of these structures match that of natural iriomoteolide-1a. The real structure of this biologically potent natural product remains veiled, waiting for collective effort in the synthetic community.

## 3. Materials and Methods

With regard to the general techniques used in this study, all moisture sensitive reactions were carried out under argon atmosphere. Anhydrous solvents were obtained as follows: THF and DME distilled from sodium and benzophenone; dichloromethane, toluene, triethylamine and diisopropylamine, distilled from CaH_2_. Column chromatography was performed with 230–400 mesh silica gel under low pressure of 5–10 psi. TLC was carried out with silica gel 60-F-254 plates, visualized under UV light and stained with phosphomolybdic acid. In addition, 1H NMR and 13C NMR spectra were recorded on Bruker Avance ARX- 400 (400 and 100 MHz), or Bruker DRX500 (500 and 125 MHz) spectrometers. High and low resolution mass spectra were carried out by the Mass Spectroscopy Center at Purdue University. HPLC analysis and preparative HPLC were performed on Agilent 1100 Series instruments (Agilent Technologies, Santa Clara, CA, USA, Agilent 1200 Series Autosampler used for analytical work).

**(*S*)-4-(1-phenyl-1H-tetrazol-5-ylthio)butane-1,2-diol** (**13**): To a stirred solution of alcohol **10** (1.505 g, 10.3 mmol), 1-(4-Hydroxyphenyl)-1*H*-tetrazole-5-thiol (3.67 g, 20.6 mmol) and triphenylphosphine (4.05 g, 15.5 mmol) in THF (30 mL), we added DIAD (3.6 mL, 18.5 mmol) at 0 °C. The reaction mixture was warmed up to rt and stirred overnight, before it was poured into sat NaHCO_3_ (20 mL). The organic layer was separated and the aq layer was extracted with Et_2_O; the combined organic layer was washed with water and brine, dried over MgSO_4_ and concentrated in vacuo. Column chromatography (20% EtOAc/hexanes) provided the corresponding sulfide as colorless oil (2.68 g, 85%). R_f_ value (EtOAc/hexane 1:1): 0.75; [α]_D_^20^ = +12.5 (*c =* 1.0, CHCl_3_); IR (film, cm^−1^) 3384, 2936, 1644, 1596, 1499, 1462, 1388, 1318, 1280, 1074, 760; ^1^H NMR (400 MHz, CDCl_3_): 7.58 (brs, 5 H), 4.13–3.85 (m, 2 H), 3.81–3.68 (m, 1 H), 3.68–3.56 (m, 2 H), 3.55–3.42 (m, 1 H), 2.55 (brs, 1 H), 2.09–1.90 (m, 2 H); ^13^C NMR (100 MHz, CDCl_3_): 155.1, 133.5, 130.4, 129.9, 124.0, 69.5, 66.4, 33.7, 29.8.

To a stirred solution of the above acetonide (2.60 g, 8.49 mmol) in MeOH (100 mL), we added *p*-TsOH (320 mg, 1.70 mmol) at rt and stirred for 24 h. Et_3_N (2 mL) was added at 0 °C to quench the reaction. The mixture was concentrated in vacuo. Flash chromatography on silica gel (5% MeOH/CHCl_3_) resulted in diol **13** (2.06 g, 91%) as a white solid. R_f_ value (EtOAc/hexane/MeOH 80:20:6): 0.5; [α]_D_^20^ = +10.7 (*c =* 0.5, CHCl_3_); IR (film, cm^−1^) 3411, 3384, 2936, 1644, 1596, 1499, 1462, 1388, 1318, 1280, 1075, 759; ^1^H NMR (400 MHz, CDCl_3_): 7.58 (brs, 5 H), 4.13–3.85 (m, 2 H), 3.81–3.68 (m, 1 H), 3.68–3.56 (m, 2 H), 3.55–3.42 (m, 1 H), 2.55 (brs, 1 H), 2.09–1.90 (m, 2 H); ^13^C NMR (100 MHz, CDCl_3_): 155.1, 133.5, 130.4, 129.9, 124, 69.5, 66.4, 33.7, 29.8.

**(*S*)-5-(3-(oxiran-2-yl)propyl)-1-phenyl-1*H*-tetrazole** (**14**): To a stirred solution of diol **13** (2.06 g, 7.74 mmol) in DCM (60 mL), Bu_2_SnO (3.85 g, 15.5 mmol), triethylamine (1.3 mL, 9.29 mmol) and tosyl chloride (1.58 g, 8.12 mmol) were added at 0 °C. The reaction mixture was stirred for 6 h, followed by dilution with water (10 mL). The organic layer was separated and the aqueous layer was extracted with DCM. The combined organic extracts were dried over anhydrous MgSO_4_ and concentrated. The crude product was purified by flash column chromatography on silica gel (ethyl acetate–hexanes 1:1) to yield the corresponding tosylate (2.52 g, 81%). R_f_ value (EtOAc/hexane 1:1): 0.4; [α]_D_^20^ = +12.0 (*c =* 0.65, CHCl_3_); IR (film, cm^−1^): 3400, 3060, 2946, 1597, 1499, 1387, 1357, 1243; ^1^H NMR (400 MHz, CDCl_3_): 7.75 (d, *J* = 8.2 Hz, 2 H), 7.53 (s, 5 H), 7.30 (d, *J* = 8.2 Hz, 2 H), 4.03–3.92 (m, 4 H), 3.50–3.43 (m, 2 H), 2.03–1.87 (m, 2 H), 2.39 (s, 3 H); ^13^C NMR (100 MHz, CDCl_3_): 154.4, 144.9, 133.3, 132.3, 130.1, 129.8, 129.7, 127.7, 123.6, 73.1, 66.9, 32.7, 29.2, 21.4.

To a stirred solution of the above tosylate (3.54 g, 8.79 mmol) in CH_3_OH–DCM (9:1, 90 mL), K_2_CO_3_ (1.58 g, 11.4 mmol) was added at 0 °C. The reaction mixture was stirred at room temperature for 1 h, concentrated, and then diluted with dichloromethane (30 mL) and water (10 mL). The organic layer was separated and the aqueous layer was extracted with dichloromethane. The combined organic extracts were dried over anhydrous MgSO_4_ and concentrated to provide **14** (1.58 g, 82%) as a colorless oil. R_f_ value (EtOAc/hexane 1:1): 0.65; [α]_D_^20^ = −15.5 (*c =* 1, CHCl_3_); IR (film, cm^−1^): 3056, 2991, 2924, 1596, 1500, 1461; ^1^H NMR (400 MHz, CDCl_3_): 7.56–7.49 (m, 5 H), 3.54–3.45 (m, 2 H), 3.06–3.01 (m, 1 H), 2.78–2.74 (m, 1 H), 2.52 (dd, *J* = 4.6, 2.7 Hz, 1 H) 2.29–2.21 (t, *J* = 4.6, 1 H),1.92 (td, *J* = 14.2, 6.9 Hz, 1 H); ^13^C NMR (100 MHz, CDCl3): 153.9, 133.5, 130.1, 29.8, 129.8, 123.7, 50.6, 46.8, 32.0.

**(*R*)-5-(3-(*tert*-butyldimethylsilyloxy)-5-methylhex-5-enylthio)-1-phenyl-1*H*-tetrazole** (**8**): To a stirred solution of epoxide **14** (1.49 g, 6 mmol) and CuCN (54 mg, 0.6 mmol) in THF (40 mL) at −78 °C, we added isopropenylmagnesium bromide (3.6 mL, 0.9 mmol). The resulting suspension was warmed up to 0 °C and stirred for 30 min. The reaction mixture was cooled again to −78 °C and more vinylmagnesium bromide (12 mL, 6 mmol) was added dropwise. The reaction mixture was warmed up to 0 °C and stirred for 1 h, before 20 mL of saturated NH_4_Cl and 10 mL of NH_4_OH were added to quench the reaction. The layers were separated and the aqueous layer was extracted with diethyl ether (3 × 30 mL). The combined organic extracts were washed with brine and dried over anhydrous magnesium sulfate. Filtration and concentration under reduced pressure gave a crude product. Flash chromatography on silica gel (20% EtOAc/hexanes) afforded the corresponding alcohol as a colorless oil (1.6 g, 92%). R_f_ value (EtOAc/hexane 1:2) 0.25; [α]_D_^20^ = +15.8 (*c =* 2, CHCl_3_); IR (film, cm^−1^): 3414, 2932, 1596, 1500, 1388, 1074, 761; ^1^H NMR (400 MHz, CDCl_3_) δ 7.63–7.48 (m, 1H), 4.85 (s, 1H), 4.77 (s, 1H), 3.89 (ddq, *J* = 12.8, 6.5, 3.3 Hz, 1H), 3.58–3.48 (m, 1H), 2.75 (s, 1H), 2.20 (d, *J* = 6.5 Hz, 1H), 2.09–1.97 (m, 1H), 1.89 (ddd, *J* = 21.4, 8.0, 6.2 Hz, 1H), 1.73 (s, 1H); ^13^C NMR (100 MHz, CDCl_3_) δ 154.7, 142.1, 133.5, 130.1, 129.7, 123.7, 113.6, 66.8, 45.8, 36.6, 29.9, 22.4.

To a stirred solution of the above alcohol (942mg, 3.24 mmol) in DMF (6 mL), we added imidazole (353 mg, 5.18 mmol) and TBSCl (538 mg, 3.57 mmol), respectively, at 0 °C. The reaction mixture was stirred at 23 °C for 12 h. A solution of saturated NaHCO_3_ (aq) was added and the aqueous layer was extracted by diethyl ether_._ The combined organic extracts were washed with water and brine, dried over anhydrous MgSO_4_, filtered, and concentrated in vacuo. Flash chromatography (4% EtOAc/hexanes) gave the silyl ether **8** (1.25 g, 95%) as a colorless oil. R_f_ value (EtOAc/hexane 1:10) 0.45; [α]_D_^20^ = +12.5 (*c =* 1, CHCl_3_); IR (film, cm^−1^): 2953, 2929, 2857, 1598, 1500, 1387, 1074, 821, 775; ^1^H NMR (400 MHz, CDCl_3_) δ 7.81–7.37 (m, 5H), 4.77 (s, 1H), 4.70 (s, 1H), 3.96 (tdd, *J* = 7.5, 5.4, 4.0 Hz, 1H), 3.55–3.34 (m, 2H), 2.27 (dd, *J* = 13.6, 5.3 Hz, 1H), 2.17 (dd, *J* = 13.6, 7.6 Hz, 1H), 2.09–1.95 (m, 1H), 1.93–1.79 (m, 1H), 1.71 (s, 3H), 0.88 (s, 9H), 0.06 (d, *J* = 1.0 Hz, 6H); ^13^C NMR (100 MHz, CDCl_3_) δ 154.3, 141.8, 133.6, 129.9, 129.7, 123.7, 113.5, 69.3, 45.8, 35.6, 29.5, 25.8, 22.8, 17.9, −4.4, −4.7. MS (ESI, *m*/*z*) [M+Na]^+^ 427.2.

**Acetonide 15**: To a stirred solution of olefin **8** (1.23 g, 3 mmol) and aldehyde **7** (645 mg, 3.3 mmol) in DCM (30 mL), we added SnCl_4_ (4.5 mL, 1 M soln in DCM, 4.5 mmol) at −78 °C; the reaction mixture was warmed up to 0 °C over 1 h and stirred for 4 h. The reaction mixture was then poured into sat NaHCO_3_ (20 mL) with crushed ice. The organic layer was separated and the aq layer was extracted with diethyl ether (3 × 30 mL). The combined organic extracts were washed with water and brine, dried on anhyd MgSO_4_ and evaporated in vacuo. The crude product was purified by flash chromatography (EtOAc/hexane 1:3) to give the corresponding diol (1.4 g, 78% yield). To a stirred solution of the diol (1.4 g, 2.34 mmol) and 2-methoxypropene (0.66 mL, 7 mmol) in DCM (40 mL), we added PPTS (50 mg, 0.2 mmol) at 0 °C. The reaction mixture was stirred at 0 °C for 1 h before Et_3_N (1.0 mL) was added. The solvents were removed in vacuo and the crude product was purified by flash chromatography (EtOAc/hexane 1:10) to give the acetonide **12** (1.47 g, 98%) as a colorless oil. R_f_ value (EtOAc/hexane 1:4): 0.55; [α]_D_^20^ = −1.8 (*c =* 1, CHCl_3_); IR (film, cm^−1^) 3068, 2886, 1500, 1410, 1097; ^1^H NMR (400 MHz, CDCl_3_) δ7.56–7.53 (m, 5H), 7.32–7.25 (m, 5H), 4.90 (s, 1H), 4.85 (s, 1H), 4.55 (AB, *J*_AB_ = 12.0 Hz, Δυ_AB_ = 20.7 Hz, 2H), 4.13–4.10 (m, 1H), 4.0–3.97 (m 1H), 3.52–3.40 (m, 2H), 3.43–3.38 (m, 2H), 2.50–2.45 (m, 1H), 2.22–2.13 (m, 3H), 2.02–1.99 (m, 1H), 1.86–1.81 (m, 1H), 1.46 (s, 3H), 1.35 (s, 3H), 1.11 (s, 3H), 0.88 (s, 9H), 0.06 (s, 6H); ^13^C NMR (400 MHz, CDCl_3_) δ 154.3, 143.0, 138.2, 133..6, 130.0, 129.7, 128.2, 127.5, 127.3, 123.7, 114.8, 107.2, 81.6, 78.7, 74.8, 73.5, 69.3, 44.2, 36.4, 35.4, 29.4, 28.6, 26.5, 25.8, 19.2, 18.0, −4.3, −4.8; MS (ESI, *m*/*z*) [M + Na]^+^ 661.3.

**Sulfone 5**: To a stirred solution of thus obtained sulfide **15** (502 mg, 0.79 mmol) in ethanol (13 mL), we added a soln of ammonium molybdate (320 mg, 0.26 mmol) in hydrogen peroxide (1.6 mL) and water (0.8 mL) at rt. The reaction mixture was stirred for 12 h and poured into a mixture of sat NaHCO_3_ (10 mL) and sodium thiosulfate (10 mL). The organic layer was separated and the aq layer was extracted with diethyl ether (3 × 30 mL). The combined organic extracts were washed with water and brine, dried on anhyd MgSO_4_ and evaporated in vacuo. The crude product was purified by flash chromatography (EtOAc/hexane 1:10) to give the sulfone **5** as a colorless oil (519 mg, 98% yield). R_f_ value (EtOAc/hexane 1:4): 0.5; [α]_D_^20^ = −2.6 (*c =* 1, CHCl3); IR (film, cm^−1^) 3038, 2887, 1512, 1215, 1097; ^1^H NMR (400 MHz, CDCl_3_) δ7.69–7.58 (m, 5H), 7.33–7.27 (m, 5H), 4.94 (s, 1H), 4.87 (s, 1H), 4.56 (AB, *J*_AB_ = 12.0 Hz, Δυ_AB_ = 20.7 Hz, 2H), 4.12–4.05 (m, 1H), 4.07–4.03 (m 1H), 3.90–3.73 (m, 2H), 3.47–3.41 (m, 2H), 2.54–2.50 (m, 1H), 2.22–2.17 (m, 2H), 2.15–2.10 (m, 2H), 2.0–1.92 (m, 1H), 1.46 (s, 3H), 1.36 (s, 3H), 1.14 (s, 3H), 0.90 (s, 9H), 0.09 (s, 3H), 0.08 (s, 3H); ^13^C NMR (100 MHz, CDCl_3_) δ 153.3, 142.8, 138.1, 133, 131.3, 129.6, 128.3, 127.5, 127.4, 125, 115.2, 107.3, 81.5, 79.1, 77.4, 76.7, 75, 73.5, 68.2, 52.3, 43.9, 36.4, 28.6, 28, 26.5, 25.8, 19.2, 17.9, −4.4, −4.9; MS (ESI, *m*/*z*) [M + Na]+ 693.3. HRMS (ESI) [M + Na]^+^ calcd for C_34_H_50_N_4_O_6_SSiNa 693.3118, found 693.3111.

**(2*R*,3*S*)-3-(methoxymethoxy)-2-methylpent-4-en-1-ol** (**20**): To a stirred solution of racemic alcohol **19** (15.2 g, 88.4 mmol) in vinyl acetate (60 mL) and pentane (120 mL), we added immobilized lipase PS 30 (20% on celite, 15.2 g); the suspension was stirred at 23 °C for 30 h, monitored by ^1^H NMR, until a half conversion was obtained. Suction filtration furnished a crude product, which was purified by flash chromatography (EtOAc/hexane 1:1) to give the (+)-alcohol as a colorless oil (7.70 g, 44%), along with corresponding acetate (9.3 g, 49%). R_f_ value (EtOAc/hexane 1:5) 0.45; [α]_D_^20^ = +8.8 (*c =* 1.7, CHCl_3_); IR (film, cm^−1^): 3422, 2981, 1744, 1372, 1236, 1026, 947; ^1^H NMR (400 MHz, CDCl_3_) δ 5.90–5.68 (m, 1H), 5.22 (d, *J* = 17.3 Hz, 1H), 5.11–4.95 (m, 1H), 4.40 (s, 1H), 3.38 (s, 1H), 2.54–2.18 (m, 2H), 1.38 (d, *J* = 1.5 Hz, 9H); ^13^C NMR (100 MHz, CDCl_3_) δ 171.4, 139.0, 114.9, 81.1, 68.9, 42.1, 27.9.

To a stirred solution of diisopropylamine (15.8 mL, 0.112 mol) in THF (80 mL) at −78 °C, we added *n*-BuLi (1.6 M in hexane, 71.8 mL, 0.107 mol) dropwise. The mixture was kept at −78 °C for 20 min before a solution of the above alcohol (7.4 g, 42.9 mmol) was added dropwise. After another 20 min of stirring, MeI (6.7 mL, 0.107 mol) was added to the reaction mixture in a dropwise manner. The reaction was stirred at −10 °C for 4 h before 50 mL sat NH_4_Cl was added. The organic layer was separated and the aq layer was extracted with diethyl ether (3 × 50 mL). The combined organic solution was washed with water and brine, dried over anhydrous MgSO_4_, filtered, and concentrated in vacuo. Flash chromatography on silica gel (10% EtOAc/hexanes) afforded the *anti*-methyl alcohol (5.84 g, 73%) as a colorless oil. R_f_ value (EtOAc/hexane 1:5) 0.5; [α]_D_^20^ = −9.9 (*c =* 1.26, CHCl_3_); IR (film, cm^−1^): 3414, 2982, 2936, 1744, 1394, 1370, 1235, 1024, 991; ^1^H NMR (400 MHz, CDCl_3_) δ 5.79 (ddd, *J* = 17.0, 10.4, 6.2 Hz, 1H), 5.24 (dd, *J* = 17.2, 1.0 Hz, 1H), 5.13 (dd, *J* = 10.4, 1.1 Hz, 1H), 4.10 (q, *J* = 6.5 Hz, 1H), 3.01 (d, *J* = 5.9 Hz, 1H), 2.41 (p, *J* = 7.1 Hz, 1H), 1.40 (s, 9H), 1.09 (dd, *J* = 7.2, 1.0 Hz, 3H); ^13^C NMR (100 MHz, CDCl_3_) δ 174.7, 138.2, 116.2, 80.9, 74.6, 45.7, 27.9.

To a stirred solution of the above alcohol (5.41 g, 29.1 mmol) in DMF (20 mL), we added DIPEA (12.6 mL, 72.6 mmol), MOMCl (4.4 mL, 58.2 mmol), and the catalytic amount of TBAI at 0 °C, respectively. The reaction mixture was stirred for 12 h before poured into saturated NaHCO_3_ (aq) and the aqueous layer was extracted by Et_2_O (3 × 50 mL). The combined organic extracts were washed with water and brine, dried over anhydrous MgSO_4_, filtered, and concentrated in vacuo. Flash chromatography on silica gel (5% EtOAc/hexanes) afforded the MOM ether (6.42 g, 96%) as a colorless oil. R_f_ value (EtOAc/hexane 1:5) 0.65; [α]_D_^20^ = +70 (*c =* 1.34, CHCl_3_); IR (film, cm^−1^): 2946, 2936, 1745, 1394, 1371, 1291, 1160, 1026, 991, 845; ^1^H NMR (400 MHz, CDCl_3_) δ 5.79 (ddd, *J* = 17.0, 10.4, 6.2 Hz, 1H), 5.24 (dd, *J* = 17.2, 1.0 Hz, 1H), 5.13 (dd, *J* = 10.4, 1.1 Hz, 1H), 4.65 (d, *J* = 6.7 Hz, 1H), 4.47 (d, *J* = 6.7 Hz, 1H), 4.10 (q, *J* = 6.5 Hz, 1H), 3.34 (s, 3H), 3.01 (d, *J* = 5.9 Hz, 1H), 2.41 (p, *J* = 7.1 Hz, 1H), 1.40 (s, 9H), 1.09 (dd, *J* = 7.2, 1.0 Hz, 3H); ^13^C NMR (100 MHz, CDCl_3_) δ 174.7, 138.2, 116.2, 94.5, 80.9, 74.6, 55.6, 45.7, 27.9.

To a stirred suspension of LiAlH_4_ (1.54 g, 38.5 mmol) in Et_2_O (40 mL), a solution of the above ester (4.44 g, 19.3 mmol) in Et_2_O (10 mL) was transferred in at 0 °C. The reaction mixture was stirred at 0 °C for 1 h before being quenched by adding 20 mL saturated NH_4_Cl (aq) and 20 mL 25% Rochelle salt solution. The mixture was stirred at rt for 4 h and extracted by Et_2_O (3 × 50 mL). The combined organic extracts were washed with water and brine, dried over anhydrous Na_2_SO_4_, filtered, and concentrated in vacuo. Flash chromatography on silica gel (25% Et_2_O/hexanes) afforded alcohol **20** (3.06 g, 99%). R_f_ value (EtOAc/hexane 1:2) 0.4; [α]_D_^20^ = +150 (*c =* 2.04, CHCl_3_); IR (film, cm^−1^): 3440, 3090, 1612, 1394, 1291, 1156, 1025; ^1^H NMR (400 MHz, CDCl_3_) δ 5.75 (ddd, *J* = 17.0, 10.7, 7.3 Hz, 1H), 5.29–5.19 (m, 2H), 4.66 (d, *J* = 6.6 Hz, 1H), 4.54 (d, *J* = 6.7 Hz, 1H), 4.19–4.11 (m, 1H), 3.69–3.59 (m, 1H), 3.55–3.48 (m, 1H), 3.37 (s, 3H), 2.57 (s, 1H), 2.00–1.87 (m, 1H), 0.88 (d, *J* = 7.0 Hz, 3H); ^13^C NMR (100 MHz, CDCl_3_) δ 135.5, 118, 94.2, 79.6, 65.2, 55.6, 39.6, 11.7. MS (ESI, *m*/*z*) [M + Na]^+^ 183.1.

**(4*R*,5*S*)-methyl 5-(methoxymethoxy)-4-methylhept-6-en-2-ynoate** (**21**): To a stirred solution of DMSO (3.7 mL, 52.3 mmol) in DCM (50 mL), we added (COCl)_2_ (2.7 mL, 31.4 mmol) at −78 °C. The mixture was stirred for 5 min, before a solution of alcohol **14** (3.35 g, 20.9 mmol) in DCM (10 mL) was added dropwise. The resulting suspension was stirred at −78 °C for 30 min. Et_3_N (14.6 mL, 0.105 mol) was added slowly. The reaction mixture was stirred at −78 °C for 1 h and allowed to warm up to room temperature and stirred for 30 min, before pouring it into 1M NaHSO_4_ solution. The organic layer was separated and the aqueous layer was extracted with DCM. The combined organic extracts were washed with brine, dried over anhydrous Na_2_SO_4_, filtered, and concentrated in vacuo to give crude aldehyde (3.35 g), which was used in the next step without further purification. R_f_ value (EtOAc/hexane 1:2) 0.7.

To a stirred solution of the CBr_4_ (13.86 g, 41.8 mmol) in DCM (50 mL) at 0 °C, we added PPh_3_ (21.93 g, 83.6 mmol). The reaction mixture was stirred at 0 °C for 10 min. A solution of the above aldehyde in DCM (10 mL) was added dropwise. The mixture was warmed up to 23 °C and stirred for 30 min. The reaction was poured into saturated NaHCO_3_ (aq). The aqueous layer was extracted by DCM. The combined organic extracts were washed with brine and dried over anhydrous Na_2_SO_4_, concentrated in vacuo. Flash chromatography on silica gel (5% EtOAc/hexanes) gave the dibromide (5.12 g, 78% for two steps) as a colorless oil. R_f_ value (EtOAc/hexane 1:4) 0.8; ^1^H NMR (400 MHz, CDCl_3_) δ 7.26 (s, 1H), 6.35 (d, *J* = 9.4 Hz, 1H), 5.64 (ddd, *J* = 17.3, 10.4, 7.9 Hz, 1H), 5.38–5.11 (m, 2H), 4.67 (d, *J* = 6.8 Hz, 1H), 4.50 (d, *J* = 6.8 Hz, 1H), 3.89 (dd, *J* = 7.7, 5.9 Hz, 1H), 3.37 (s, 3H), 2.72–2.55 (m, 1H), 1.32–0.82 (m, 3H); ^13^C NMR (100 MHz, CDCl_3_) δ 140.4, 135.7, 119.0, 93.6, 88.8, 79.6, 55.5, 42.9, 15.2.

To a stirred solution of the above dibromide (3.88 g, 10.1 mmol) in THF (30 mL), we added *n*-BuLi (1.6 M in hexanes, 19.0 mL, 30.3 mmol) at −78 °C. The mixture was stirred at −78 °C for 15 min before methyl chloroformate (2.34 mL, 30.3 mmol) was added. The reaction mixture was stirred at −78 °C for 1 h before pouring it into saturate NH_4_Cl (aq). The organic layer was separated and the aqueous layer was extracted by Et_2_O (3 × 50 mL). The organic extracts were combined, washed by water and brine, dried over anhydrous MgSO_4_, filtered, and concentrated in vacuo. Flash chromatography on silica gel (5% EtOAc/hexanes) produced alkynyl ester **21** (2.14 g, 99%) as a pale yellow oil. R_f_ value (EtOAc/hexane 1:10) 0.45; [α]_D_^20^ = +82 (*c =* 1.22, CHCl_3_); IR (film, cm^−1^): 2950, 2888, 1716, 1644, 1435, 1225, 1156, 1031, 918; ^1^H NMR (400 MHz, CDCl_3_) δ 5.64 (ddd, *J* = 17.0, 10.6, 7.7 Hz, 1H), 5.37–5.13 (m, 2H), 4.63 (d, *J* = 6.9 Hz, 1H), 4.49 (d, *J* = 6.9 Hz, 1H), 4.03–3.87 (m, 1H), 3.67 (s, 3H), 3.33 (s, 3H), 2.79–2.63 (m, 1H), 1.16 (d, *J* = 7.1 Hz, 3H); ^13^C NMR (100 MHz, CDCl_3_) δ 153.9, 134.7, 119.8, 93.5, 90.3, 78.5, 73.8, 55.5, 52.3, 31.3, 15.6.

**(4*R*,5*S*,2*Z*)-methyl-5-(methoxymethoxy)-3,4-dimethylhepta-2,6-dienoate** (**22**): To a suspension of CuI (5.42 g, 28.4 mmol) in THF (60 mL), we added MeLi (28.5 mL, 45.6 mmol) at −60 °C. The mixture was slowly warmed up to 0 °C to obtain a clear solution. A soln of alkynyl ester **21** (2.41 g, 11.4 mmol) in THF (5 mL) was added slowly at −60 °C and stirred at −40 °C for 2 h. AcOH (2.74 mL, 47.9 mmol) was added to quench the reaction, followed by sat NH_4_Cl (50 mL). The organic layer was separated and the aqueous layer was extracted by Et_2_O (3 × 50 mL). The combined organic extracts were washed with water and brine, dried over anhydrous MgSO_4_, filtered, and concentrated in vacuo. Flash chromatography on silica gel (5% EtOAc/hexanes) produced *Z*-enoate **22** (2.50 g, 96%) as a colorless oil. R_f_ value (EtOAc/hexane 1:10) 0.45; [α]_D_^20^ = +56 (*c =* 0.3, CHCl_3_); IR (film, cm^−1^): 2951, 2889, 1718, 1646, 1227, 1157, 1031, 1093, 919, 859; ^1^H NMR (300 MHz, CDCl_3_) δ 5.71 (brs, 1H), 5.28–5.19 (m, 1H), 4.66 (d, *J* = 7 Hz, 1H), 4.47 (d, *J* = 7 Hz, 1H), 4.13 (dq, *J* = 8.8, 7 Hz, 1H), 3.95 (t, *J* = 8.5 Hz, 1H), 3.65 (s, 3H), 3.33 (s, 3H), 1.88 (s, 3H), 0.98 (d, *J* = 7 Hz, 3H); ^13^C NMR (75 MHz, CDCl_3_) δ 166.5, 161.8, 136.6, 119.2, 93.5, 80.1, 55.7, 50.7, 38.3, 30, 19.9, 15.1.

**(2*R*,3*R*,*Z*)-6-(*tert*-butyldimethylsilyloxy)-2-(methoxymethoxy)-3,4-dimethylhex-4-enal** (**6**): To a stirred soln of ester **22** (2.43 g, 10.6 mmol) in DCM (50 mL), we added DIBAL-H (31.9 mL, 31.9 mmol) at −78 °C. The reaction mixture was stirred 1 h, before the addition of 20 mL saturated NH_4_Cl (aq) and 20 mL 25% Rochelle salt solution. The mixture was stirred at rt for 4 h and extracted by Et_2_O (3 × 50 mL). The combined organic extracts were washed with water and brine, dried over anhydrous Na_2_SO_4_, filtered, and concentrated in vacuo. Flash chromatography on silica gel (25% Et_2_O/hexanes) afforded the allyl alcohol (2.02 g, 95%). R_f_ value (EtOAc/hexane 1:4) 0.25; [α]_D_^20^ = +106 (*c =* 2, CHCl_3_); IR (film, cm^−1^): 3349, 2962, 2823, 1613, 1444, 1227, 1152, 1028, 916; ^1^H NMR (400 MHz, CDCl_3_): 5.69 (t, *J =* 7.1 Hz, 1H), 5.62–5.50 (m, 1H), 5.26 (d, *J =* 10.1 Hz, 1H), 5.20 (d, *J =* 17.1 Hz, 1H), 4.63 (d, *J* = 6.9 Hz, 1H), 4.35 (d, *J* = 6.9 Hz, 1H), 4.18 (dd, *J* = 8.8, 7.0 Hz, 1H), 3.82–3.75 (m, 2H), 3.26 (s, 3H), 2.85–2.75 (m, 2H). 1.67 (s, 3H), 0.88 (d, *J* = 7.0 Hz, 3H); ^13^C NMR (75 MHz, CDCl_3_) δ 141.9, 136.3, 126.4, 119.7, 92.8, 78.4, 57, 55.5, 38.1, 18.1, 15.1.

To a stirred solution of the above alcohol (1.45 g, 7.24 mmol) in DCM (50 mL), we added imidazole (739 mg, 10.9 mmol) and TBSCl (1.2 g, 7.96 mmol) at 0 °C. The reaction was warmed up to rt and stirred for 1 h, before pouring it into a mixture of sat NaHCO_3_ (50 mL) and crushed ice. The mixture was extracted with ethers (3 × 60 mL) and the organic layer was washed with water and brine, dried over anhyd MgSO_4_ and evaporated in vacuo to give the crude TBS ether as a clear oil, which was used for the next step without further purification. Flash chromatography on silica gel (25% EtOAc/hexanes) afforded the silyl ether (2.28 g, 99%). R_f_ value (EtOAc/hexane 1:4) 0.85; [α]_D_^20^ = +34 (*c =* 1.05, CHCl_3_); IR (film, cm^−1^): 2960 2821, 1607, 1227, 1152, 1091, 916; ^1^H NMR (300 MHz, CDCl_3_): 5.62–5.53 (m, 1H), 5.38 (t, *J =* 7.1 Hz, 1H), 5.25–5.15 (m, 2H), 4.62 (d, *J* = 6.9 Hz, 1H), 4.39 (d, *J* = 6.9 Hz, 1H), 4.32–4.23 (m, 1H), 4.2–4.1 (m, 1H), 3.82 (t, *J =* 8.5 Hz, 1H), 3.30 (s, 3H), 2.72–2.62 (m, 1H). 1.69 (d, *J =* 1 Hz, 3H), 0.91 (d, *J* = 7.0 Hz, 3H), 0.88 (s, 9H), 0.05 (s, 6H); ^13^C NMR (75 MHz, CDCl_3_) δ 137.7, 137, 127.3, 118.7, 93.2, 79.8, 60, 55.3, 38.8, 25.9, 18.7, 18.3, 15.1, −5.2.

To a stirred solution of above olefin (1.09 g, 3.47 mmol) in dioxane (24 mL) and water (8 mL), we added 2,6-lutidine (2.02 mL, 17.4 mmol), OsO_4_ (2.5% in *t*-BuOH, 1.74 mL, 0.14 mmol) and NaIO_4_ (2.97 g, 12.9 mmol) at 0 °C. The mixture was stirred at 0 °C for 18 h before saturated NaHCO_3_ (10 mL) and NaS_2_O_3_ (10 mL) were added. The mixture was stirred for another 30 min, extracted by EtOAc. The combined organic extracts were dried over anhydrous Na_2_SO_4_, filtered, and concentrated in vacuo. Flash chromatography on silica gel (25% EtOAc/hexanes) produced the aldehyde **6** (736 mg, 67%). R_f_ value (EtOAc/hexane 1:10) 0.65; [α]_D_^20^ = +28 (*c =* 0.5, CHCl_3_); ^1^H NMR (400 MHz, CDCl_3_) δ 9.53 (dd, *J* = 3.1, 0.5 Hz, 1H), 5.41 (t, *J* = 6.2 Hz, 1H), 4.61 (dd, *J* = 22.5, 6.9 Hz, 3H), 4.23 (dd, *J* = 13.0, 7.0 Hz, 1H), 4.17–4.04 (m, 1H), 3.70 (dd, *J* = 8.2, 3.3 Hz, 1H), 3.35 (d, *J* = 0.6 Hz, 4H), 3.00 (p, *J* = 7.2 Hz, 1H), 1.69 (s, 4H), 1.01 (d, *J* = 7.0 Hz, 4H), 0.90 (s, 3H), 0.88 (s, 9H), 0.05 (s, 6H); ^13^C NMR (100 MHz, CDCl_3_) δ 202.2, 136.0, 128.2, 96.8, 84.7, 59.3, 55.9, 34.9, 25.9, 18.9, 18.3, 14.3, −5.3.

**Coupling product (23):** To a stirred solution of sulfone **5** (628 mg, 0.94 mmol) in DME (30 mL), we added KHMDS (1.85 mL, 0.5 M soln in toluene, 0.93 mmol) at −78 °C. The reaction mixture was stirred for 30 min, before a soln of aldehyde 6 (357 mg, 1.13 mmol) in DME (5 mL) was transferred in. The reaction mixture was stirred for another 30 min, before it was warmed up to rt. The reaction was quenched by sat NH_4_Cl (10 mL) at −78 °C. The organic layer was separated and the aq layer was extracted with diethyl ether (3 × 20 mL). The combined organic extracts were washed with water and brine, dried on anhydrous MgSO_4_ and evaporated in vacuo. The crude product was purified by flash chromatography (EtOAc/hexane 1:30) to give coupling product **23** as a colorless oil (544 mg, 76% yield). R_f_ value (EtOAc/hexane 1:10): 0.5; [α]_D_^20^ = +14 (*c* = 0.6, CHCl_3_); IR (film, cm^−1^) 2928, 1455, 1248, 1108; ^1^H NMR (400 MHz, CDCl_3_) δ 5.73–5.54 (m, 1H), 5.40 (t, *J* = 6.1 Hz, 1H), 5.23 (dd, *J* = 15.4, 8.5 Hz, 1H), 4.88 (d, *J* = 32.2 Hz, 2H), 4.68 (d, *J* = 6.8 Hz, 1H), 4.56 (d, *J* = 5.1 Hz, 2H), 4.41 (d, *J* = 6.8 Hz, 1H), 4.21 (d, *J* = 6.1 Hz, 3H), 4.14 (dd, *J* = 8.7, 3.7 Hz, 1H), 3.86 (t, *J* = 8.3 Hz, 2H), 3.41 (q, *J* = 9.8 Hz, 2H), 3.30 (s, 3H), 2.35–2.09 (m, 8H), 1.63 (s, 3H), 1.44 (s, 3H), 1.35 (s, 3H), 1.13 (s, 3H), 0.95 (d, *J* = 7.0 Hz, 3H), 0.9 (s, 9H), 0.87 (s, 9H), 0.12–−0.07 (m, 12H); ^13^C NMR (100 MHz, CDCl_3_) δ 143.50, 138.19, 131.28, 130.98, 128.21, 127.41, 127.29, 125.83, 114.25, 107.15, 93.11, 81.53, 79.29, 78.34, 74.98, 73.45, 70.52, 60.11, 55.31, 46.97, 43.68, 39.56, 36.49, 28.56, 26.44, 25.90, 25.80, 19.16, 18.27, 17.97, 15.76, 13.52, −4.59, −4.65, −5.15. MS (ESI, *m*/*z*) [M + Na]^+^ 783.5.

**Aldehyde (3):** To a stirred solution of benzyl ether **23** (550 mg, 0.72 mmol) in THF (10 mL) and allyl ethyl ether (1 mL), we transferred a soln of lithium metal (50 mg, 7.2 mmol) in liquid ammonia (12 mL) in portions at −78 °C. The reaction was carefully monitored by TLC and stopped immediately after the solution became slightly blue. Ammonium chloride (2 g) was added to quench the reaction. The mixture was allowed to warm up to rt to evaporate the ammonia, before water (10 mL) was added. The organic layer was separated and the aq layer was extracted with diethyl ether (3 × 20 mL). The combined organic extracts were washed with water and brine, dried over anhyd MgSO_4_ and evaporated in vacuo. The crude product was purified by flash chromatography (EtOAc/hexane 1:10) to give the alcohol as a colorless oil (402 mg, 83% yield), along with the recovered starting material. R_f_ value (EtOAc/hexane 1:4): 0.43; [α]_D_^20^ = +36.0 (*c =* 1.2, CHCl_3_); IR (film, cm^−1^) 3410, 2954, 1253, 1096; ^1^H NMR (400 MHz, CDCl_3_) δ5.69–5.62 (m, 1H), 5.37 (t, *J* = 4.2 Hz, 1H), 5.25 (dd, *J* = 15.5, 8.6 Hz, 1H), 4.95 (s, 1H), 4.87 (s, 1H), 4.62 (d, *J* = 6.8 Hz, 1H), 4.36 (d, *J* = 6.8 Hz, 1H), 4.28 (dd, *J* = 8.0, 5.2 Hz, 1H), 4.22–4.18 (m 1H), 4.2–4.12 (m, 1H), 3.9–3.82 (m, 1H), 3.80 (t, *J* = 8.7 Hz, 1H), 3.53 (dd, *J* = 12.0, 9.8 Hz, 1H), 3.38–3.33 (m, 1H), 3.30 (s, 3H), 2.58–2.50 (m, 1H), 2.32–2.04 (m, 6H), 1.68 (s, 3H), 1.45 (s, 3H), 1.36 (s, 3H), 1.06 (s, 3H), 0.92 (d, *J* = 6.8 Hz, 3H), 0.90 (s, 9H), 0.87 (s, 9H), 0.06–0.03 (m, 12H); ^13^C NMR (100 MHz, CDCl_3_) δ 143.0, 138.0, 131.6, 131.4, 127.2, 114.6, 107.0, 93.0, 82.6, 79.1, 75.9, 70.8, 65.4, 59.8, 55.3, 43.5, 39.7, 39.1, 36.1, 28.6, 26.6, 25.9, 25.8, 18.6, 18.3, 18.0, 15.4, −4.6, −5.2.

To a suspension of the above alcohol (352 mg, 0.53 mmol) and sodium bicarbonate (265 mg, 3.2 mmol) in DCM (20 mL), we added Dess–Martin periodinane (445 mg, 1.05 mmol) at rt. The reaction mixture was stirred for 1 h before it was poured into a mixture of sat NaHCO_3_ (10 mL) and sodium thiosulfate (10 mL). The organic layer was separated and the aq layer was extracted with diethyl ether (3 × 20 mL). The combined organic extracts were washed with water and brine, dried on anhyd MgSO_4_ and evaporated in vacuo to give the crude aldehyde **3** (352 mg, quantitative), which was used directly in the next step without further purification. ^1^H NMR (400 MHz, CDCl_3_) δ 9.61 (s, 1H), 5.70–5.65 (m, 1H), 5.41 (t, *J* = 5.3 Hz, 1H), 5.28 (dd, *J* = 15.5, 8.6 Hz, 1H), 4.97 (s, 1H), 4.91 (s, 1H), 4.66 (d, *J* = 6.8 Hz, 1H), 4.37 (d, *J* = 6.8 Hz, 1H), 4.30 (dd, *J* = 8.0, 5.2 Hz, 1H), 4.21–4.17 (m 2H), 3.90–3.82 (m, 2H), 3.30 (s, 3H), 2.7–2.65 (m, 1H), 2.25–2.12 (m, 6H), 1.69 (s, 3H), 1.50 (s, 3H), 1.46 (s, 3H), 1.20 (s, 3H), 0.92-0.87 (m, 21H), 0.1–0.05 (m, 12H).

**(3*S*,4*S*)-4-(*tert*-butyldimethylsilyloxy)-3-methylpentanal (16):** To a stirred solution of alcohol **12** (7.31 g, 73 mmol) in DMF (70 mL), we added imidazole (5.96 g, 87.6 mmol) and TBSCl (11 g, 73 mmol) at 0 °C. The reaction was warmed up to rt and stirred for 6 h. The reaction mixture was then poured into a mixture of sat NaHCO_3_ (50 mL) and crushed ice. The mixture was extracted with ethers (3 × 60 mL) and the organic layer was washed with water and brine, dried over anhyd MgSO_4_ and evaporated in vacuo to give the crude TBS ether as a clear oil, which was used for the next step without further purification. To a stirred solution of thus obtained olefin in THF (60 mL), we added the BH_3_·THF complex (73 mL, 1 M soln in THF, 73 mmol) at 0 °C. The reaction mixture was allowed to warm up to rt and stirred for 6 h. NaOH (10 mL) and H_2_O_2_ (15 mL, 70% soln) were added and the mixture was refluxed for 1 h. The organic layer was separated and the aq layer was extracted with Et_2_O; the combined organic layer was washed with water and brine, dried over MgSO_4_ and concentrated in vacuo. Column chromatography (EtOAc/hexane 1:20) provided corresponding alcohol **16** as a colorless oil (11.4 g, 67% for two steps). R_f_ value (EtOAc/hexane 1:10): 0.53; [α]_D_^20^ = +2.3 (*c =* 1.0, CHCl_3_); IR (film, cm^−1^) 3340, 2932, 2858, 1463, 1254, 1053; ^1^H NMR (400 MHz, CDCl_3_) δ3.77–3.71 (m, 1H), 3.69–3.62 (m, 1H), 3.59–3.53 (m, 1H), 3.19 (m, 1H), 1.71–1.66 (m, 2H), 1.39–1.34 (m, 1H), 1.1 (d, *J* = 6.4 Hz, 3H), 0.87 (s, 9H), 0.81 (d, *J* = 6.7 Hz, 3H), 0.04 (s, 6H); ^13^C NMR (100 MHz, CDCl_3_) δ 61.8, 38.5, 27.3, 35.2, 25.7, 18.2, 17.9, 17.2, −4.7, −5.1.

**(3*S*,4*R*,6*S*,7*S*)-7-(*tert*-butyldimethylsilyloxy)-3,6-dimethyloct-1-en-4-ol (17):** To a stirred solution of DMSO (2.7 mL, 37.8 mmol) in DCM (70 mL), we added oxalyl chloride (2 mL, 22.7 mmol) at −78 °C. After 10 min of stirring, a solution of alcohol **16** (3.5 g, 15.1 mmol) in DCM (10 mL) was transferred in at the same temperature. The reaction mixture was stirred for 30 min before Et_3_N (10.5 mL, 75.5 mmol) was added. After stirring at −78 °C for 1 h, the reaction mixture was allowed to warm up to 0 °C for 30 min, before it was poured into sat NaHCO_3_ soln (30 mL). The organic layer was separated and the aq layer was extracted with diethyl ether (3 × 20 mL). The combined organic extracts were washed with water and brine, dried over anhyd MgSO_4_ and evaporated in vacuo. The crude product was purified by flash chromatography (EtOAc/hexane 1:30) to give the aldehyde as a colorless oil (3.03 g, 87% yield), which was used in the next step immediately. R_f_ value (EtOAc/hexane 1:10): 0.85.

To a stirred mixture of potassium *tert*-butoxide (8.7 mL, 1.0 M soln in THF, 8.7 mmol) and *trans*-2-butene (1.4 mL, 14.5 mmol) in THF (30 mL), we added *n*-butyllithium (5.5 mL, 1.6 M soln in THF, 8.7 mmol) at −78 °C. After complete addition of *n*-butyllithium, the mixture was stirred at −45 °C for 10 min. The resulting orange solution was cooled to −78 °C again and a solution of (−)-Ipc_2_BOMe (3.3 g, 10.4 mmol) in THF (10 mL) was added dropwise. After 30 min of stirring, boron trifluoride etherate (1.5 mL, 11.6 mmol) was added dropwise. Then, the above aldehyde (1.34 g, 5.8 mmol) in THF (5 mL) was transferred in. The mixture was stirred at −78 °C for 3 h before NaOH (6.8 mL, 3 M soln) and H_2_O_2_ (4.7 mL, 70% soln) were added. The contents were refluxed for 1 h. The organic layer was separated and the aq layer was extracted with diethyl ether (3 × 30 mL). The combined organic extracts were washed with water and brine, dried over anhyd MgSO_4_ and evaporated in vacuo. The crude product was purified by flash chromatography (EtOAc/hexane 1:30) to give the alcohol **17** as a colorless oil (1.28 g, 77% yield). R_f_ value (EtOAc/hexane 1:10): 0.63; [α]_D_^20^ = +6.3 (*c* = 0.67, CHCl_3_); IR (film, cm^−1^) 3411, 2959, 1462, 1045; ^1^H NMR (400 MHz, CDCl_3_) δ5.86–5.77 (m, 1H), 5.1–5.03 (m, 2H), 3.84–3.77 (m, 1H), 3.6–3.54 (m, 1H), 2.21–2.18 (m, 1H), 2.13 (d, *J* = 4.9 Hz, 1H), 1.82–1.74 (m, 1H), 1.67–1.6 (m, 1H), 1.28–1.2 (m, 1H), 1.06 (d, *J* = 6.9 Hz, 3H), 1.02 (d, *J* = 6.4 Hz, 3H), 0.88 (s, 9H), 0.92 (d, *J* = 9.1 Hz, 3H); 0.04 (s, 6H); ^13^C NMR (100 MHz, CDCl_3_) δ 140.4, 115.5, 72.8, 71.2, 43.7, 37.0, 36.4, 25.7, 19.1, 17.9, 16.5, 16.2, −4.4, −4.9; MS (ESI, *m*/*z*) [M + Na]^+^ 309.

**(3*S*,4*R*,6*S*,7*S*)-7-(*tert*-butyldimethylsilyloxy)-4-(4-methoxybenzyloxy)-3,6-dimethyloctan-1-ol (18):** To a stirred solution of alcohol **17** (710 mg, 2.5 mmol) in DMF (15 mL), we added NaH (60%, 150 mg, 3.7 mmol) at 0 °C. The mixture was stirred for 30 min before PMBCl (0.5 mL, 3.7 mmol) was added at 0 °C. After stirring at rt overnight, water (4 mL) and Et_2_NH (2 mL) were added and the mixture was stirred for 1h, before it was poured into sat NaHCO_3_ (aq). The mixture was extracted with diethyl ether (3 × 30 mL). The combined organic extracts were washed with water and brine, dried over anhyd MgSO_4_ and evaporated in vacuo. The crude product was purified by flash chromatography (EtOAc/hexane 1:30) to give the PMB ether as a colorless oil (854 mg, 84% yield). R_f_ value (EtOAc/hexane 1:10): 0.75; [α]_D_^20^ = +5.0 (*c =* 1, CHCl_3_); IR (film, cm^−1^) 2959, 1645, 1059; ^1^H NMR (500 MHz, CDCl_3_) δ 7.30 (d, *J* = 8.5 Hz, 1H), 6.91 (d, *J* = 8.5 Hz, 1H), 5.89–5.82 (m, 1H), 5.08 (d, *J* = 18.0 Hz, 1H), 5.06 (d, *J* = 9.4 Hz, 1H), 4.41 (AB, *J*_AB_ = 11.2 Hz, Δυ_AB_ = 33.1 Hz, 2H), 3.84 (s, 3H), 3.69–3.64 (m, 1H), 3.43–3.4 (m, 1H), 2.55–2.5 (m, 1H), 1.62–1.56 (m, 2H), 1.32–1.29 (m, 1H), 1.08 (d, *J* = 6.9 Hz, 3H), 1.05 (d, *J* = 6.4 Hz, 3H), 0.96 (s, 9H), 0.92 (d, *J* = 9.1 Hz, 3H); 0.06 (s, 3H); ^13^C NMR (125 MHz, CDCl_3_) δ 159.0, 140.7, 131.1, 129.4, 129.3, 114.7, 113.7, 80.8, 70.9, 55.4, 37.2, 34.6, 33.5, 25.9, 20.7, 18.1, 15.2, 15.0, −4.1, −4.7.

To a stirred solution of the above olefin (1.45 g, 3.6 mmol) in THF (30 mL), we added 9-BBN (14.2 mL, 0.5 M soln in THF, 7.2 mmol) at 0 °C. The reaction mixture was allowed to warm up to rt and stirred for 3 h. NaOH (0.6 mL) and H_2_O_2_ (4 mL) were added and the mixture was refluxed for 1 h. The organic layer was separated and the aq layer was extracted with Et_2_O; the combined organic layer was washed with water and brine, dried over Na_2_SO_4_ and concentrated in vacuo. Column chromatography provided alcohol **18** as a colorless oil (1.21 g, 79%). R_f_ value (EtOAc/hexane 1:1): 0.68; [α]_D_^20^ = +3.7 (*c =* 1, CHCl_3_); IR (film, cm^−1^) 3403, 2931, 1613, 1040; ^1^H NMR (400 MHz, CDCl_3_) δ 7.21 (d, *J* = 8.6 Hz, 1H), 6.86 (d, *J* = 8.6 Hz, 1H), 4.45 (s, 2H), 3.81 (s, 3H), 3.75–3.65 (m, 2H), 3.61–3.55 (m, 1H), 3.38–3.33 (m, 1H), 1.88–1.80 (m, 1H), 1.7–1.6 (m, 2H), 1.55–1.45 (m, 2H), 1.4–1.32 (m, 1H), 1.02 (d, *J* = 6.9 Hz, 3H), 0.95 (d, *J* = 6.4 Hz, 3H), 0.92 (s, 9H), 0.85 (d, *J* = 9.1 Hz, 3H), 0.03 (s, 3H), 0.01 (s, 3H); ^13^C NMR (100 MHz, CDCl_3_) δ 129.3, 113.6, 81.2, 71.2, 70.9, 60.1, 55.1, 37.3, 34.6, 34.3, 32.8, 32.1, 27.3, 25.8, 22.6, 18, 15.5, 15.1, −4.8; MS (ESI, *m*/*z*) [M+Na]^+^ 447.4.

**Sulfone (4)**: To a stirred solution of alcohol **18** (880 mg, 2.1 mmol), 1-(4-Hydroxyphenyl)-1*H*-tetrazole-5-thiol (739 mg, 4.1 mmol) and triphenylphosphine (814 mg, 3.11 mmol) in THF (20 mL), we added DIAD (0.72 mL, 3.7 mmol) at 0 °C. The reaction mixture was warmed up to rt and stirred overnight, before it was poured into sat NaHCO_3_ (20 mL). The organic layer was separated and the aq layer was extracted with Et_2_O; the combined organic layer was washed with water and brine, dried over MgSO_4_ and concentrated in vacuo. Column chromatography provided the sulfide as a colorless oil (1.03 g, 85%). R_f_ value (EtOAc/hexane 1:4): 0.56; [α]_D_^20^ = +1.2 (*c =* 0.5, CHCl_3_); IR (film, cm^−1^) 2956, 2886, 1614, 1074; ^1^H NMR (400 MHz, CDCl_3_) δ 7.53–7.57 (m, 5H), 7.23 (d, *J* = 8.6 Hz, 2H), 6.85 (d, *J* = 8.6 Hz, 2H), 4.45 (AB, *J*_AB_ = 11.1 Hz, Δυ_AB_ = 18 Hz, 2H), 3.78 (s, 3H), 3.66–3.63 (m, 1H), 3.55–3.49 (m, 1H), 3.37–3.31 (m, 2H), 1.93–1.89 (m, 2H), 1.7–1.62 (m, 1H), 1.62–1.55 (m, 1H), 1.52–1.48 (m, 1H), 1.31–1.25 (m, 1H), 1.02 (d, *J* = 6.8 Hz, 3H), 1 (d, *J* = 7 Hz, 3H), 0.86 (d, *J* = 6.8 Hz, 1H), 0.84 (s, 9H), 0.01 (s, 3H), 0 (s, 3H); ^13^C NMR (125 MHz, CDCl_3_) δ 129.3, 113.6, 81.2, 71.2, 70.9, 60.1, 55.1, 37.3, 34.6, 34.3, 32.8, 32.1, 27.3, 25.8, 22.6, 18.0, 15.5, 15.1, −4.8. To a stirred solution of thus obtained sulfide (353 mg, 0.57 mmol) in ethanol (9 mL), we added a solution of ammonium molybdate (320 mg, 0.26 mmol) in hydrogen peroxide (1.6 mL) and water (0.8 mL) at rt. The reaction mixture was stirred for 12 h and poured into a mixture of sat NaHCO_3_ (10 mL) and sodium thiosulfate (10 mL). The organic layer was separated and the aq layer was extracted with diethyl ether (3 × 20 mL). The combined organic extracts were washed with water and brine, dried on anhyd MgSO_4_ and evaporated in vacuo. The crude product was purified by flash chromatography (EtOAc/hexane 1:12) to give the sulfone **4** as a colorless oil (352 mg, 95% yield). R_f_ value (EtOAc/hexane 1:4): 0.5; [α]_D_^20^ = +1.8 (*c =* 1, CHCl_3_); IR (film, cm^−1^) 2931, 2857, 1612, 1513, 1249, 1074; ^1^H NMR (500 MHz, CDCl_3_) δ7.72–7.7 (m, 2H), 7.64–7.6 (m, 3H), 7.27 (d, *J* = 8.5 Hz, 1H), 6.90 (d, *J* = 8.5 Hz, 1H), 4.47 (AB, *J*_AB_ = 11.2 Hz, Δυ_AB_ = 21.8 Hz, 2H), 3.93–3.87 (m, 1H), 3.83 (s, 3H), 3.79–3.71 (m, 2H), 3.4–3.37 (m, 1H), 2.1–2.04 (m, 1H), 2.02–1.86 (m, 2H), 1.66–1.61 (m, 1H), 1.58–1.54 (m, 1H), 1.42–1.38 (m, 1H), 1.10 (d, *J* = 6.9 Hz, 3H), 1.05 (d, *J* = 6.4 Hz, 3H), 0.92 (d, *J* = 3.5 Hz, 3H), 0.90 (s, 9H), 0.07 (s, 3H), 0.04 (s, 3H); ^13^C NMR (125 MHz, CDCl_3_) δ 159.3, 153.4, 133.1, 131.4, 130.6, 129.7, 129.4, 125.1, 113.8, 80.8, 71.2, 71.0, 55.3, 54.6, 37.2, 36.6, 34.6, 33.4, 25.8, 24.2, 20.4, 18.1, 15.5, 15.2, −4.1, −4.7; MS (ESI, *m*/*z*) [M + Na]^+^ 639.3. HRMS (ESI) [M+Na]^+^ calcd for C_31_H_48_N_4_O_5_SSiNa 639.3012, found 639.3008.

**Coupling product (24)**: To a stirred solution of aldehyde **3** (422 mg, 0.64 mmol) and sulfone **4** (324 mg, 0.53 mmol) in DME (15 mL), we added KHMDS (1.6 mL, 0.5 M soln in toluene, 0.8 mmol) at −65 °C. The reaction mixture was stirred for 1 h at −65 °C, before it was warmed up to rt. The reaction was quenched by sat NH_4_Cl (5 mL) at −65 °C and the organic layer was separated and the aq layer was extracted with diethyl ether (3 × 20 mL). The combined organic extracts were washed with water and brine, dried on anhyd MgSO_4_ and evaporated in vacuo. The crude product was purified by flash chromatography (EtOAc/hexane 1:20) to give the olefin **24** as a colorless oil (376 mg, 67% yield). R_f_ value (EtOAc/hexane 1:10): 0.48; [α]_D_^20^ = +22.5 (*c =* 0.55, CHCl_3_); IR (film, cm^−1^) 2857, 1729, 1612, 1513, 1257, 1081; ^1^H NMR (500 MHz, CDCl_3_) δ 7.28 (d, *J* = 8.5 Hz, 1H), 6.89 (d, *J* = 8.5 Hz, 1H), 5.75–5.67 (m, 2H), 5.48 (d, *J* = 15.5 Hz, 1H), 5.41 (t, *J* = 3.9 Hz, 1H), 5.3–5.26 (dd, *J* = 15.5, 8.5 Hz, 1H), 4.96 (s, 1H), 4.89 (s, 1H), 4.68 (d, *J* = 6.8 Hz, 1H), 4.44 (AB, *J*_AB_ = 11.1 Hz, Δυ_AB_ = 20.6 Hz, 2H), 4.4 (d, *J* = 6.8 Hz, 1H), 4.34 (dd, *J* = 12.8, 7.1 Hz, 1H), 4.2 (dd, *J* = 12.8, 3.9 Hz, 1H), 3.94–3.87 (m, 2H), 3.86–3.83 (m, 1H), 3.83 (s, 3H), 3.7–3.66 (m, 1H), 3.4–3.32 (m, 1H), 3.34 (s, 3H), 2.69–2.65 (m, 1H), 2.34–2.28 (m, 2H), 2.25–2.18 (m, 3H), 2.14–2.1 (m, 1H), 1.9–1.83 (m, 2H), 1.74–1.7 (m, 1H), 1.72 (s, 3H), 1.65–1.6 (m, 1H), 1.58–1.53 (m, 1H), 1.49 (s, 3H), 1.4 (s, 3H), 1.21 (s, 3H), 1.08 (d, *J* = 9.7 Hz, 3H), 0.95–0.85 (m, 36H), 0.1–0.04 (m, 18H); ^13^C NMR (125 MHz, CDCl_3_) δ 159, 143.6, 138.0, 134, 131.6, 131.4, 131.1, 130, 129.3, 127.3, 114.3, 113.7, 107.2, 93.1, 82.1, 81.5, 81.2, 79.2, 71.1, 70.8, 70.7, 59.8, 55.4, 55.2, 43.8, 39.7, 39.2, 37.5, 35.7, 35.6, 32.8, 29.7, 28.5, 26.6, 26, 25.9, 20.6, 20.4, 18.7, 18.4, 18, 15.5, 15.4, 15, −4.1, −4.4, −4.5, −4.6, −4.7, −5.1; MS (ESI, *m*/*z*) [M + Na]^+^ 1081.7. HRMS (ESI) [M + Na]^+^ calcd for C_60_H_110_O_9_Si_3_Na 1081.7355, found 1081.7367.

**Diol product (25):** To a stirred solution of PMB ether **24** (204 mg, 0.2 mmol) in DCM (20 mL) and pH 7.0 buffer (1.6 mL), we added DDQ (88 mg, 0.4 mmol) at 0 °C and the reaction mixture was stirred at that temperature for 1 h. The reaction was quenched by sat NaHCO_3_ (5 mL) and the organic layer was separated. The aq layer was extracted with diethyl ether (3 × 20 mL). The combined organic extracts were washed with sat NaHCO_3_, water and brine, dried on anhyd MgSO_4_ and evaporated in vacuo. The crude product was purified by flash chromatography (EtOAc/hexane 1:15) to give the corresponding alcohol as a colorless oil (138 mg, 76% yield). R_f_ value (EtOAc/hexane 1:4): 0.67; [α]_D_^20^ = +30.1 (*c* = 0.55, CHCl_3_); IR (film, cm^−1^) 3429, 2931, 1619, 1252, 1079; ^1^H NMR (500 MHz, CDCl_3_) δ 5.77–5.67 (m, 2H), 5.48 (d, *J* = 15.5 Hz, 1H), 5.41 (t, *J* = 5.2 Hz, 1H), 5.28 (dd, *J* = 15.5, 8.3 Hz, 1H), 4.99 (s, 1H), 4.88 (s, 1H), 4.68 (d, *J* = 6.8 Hz, 1H), 4.4 (d, *J* = 6.8 Hz, 1H), 4.34 (dd, *J* = 12.9, 7.3 Hz, 1H), 4.2 (dd, *J* = 12.9, 4.1 Hz, 1H), 3.92–3.86 (m, 2H), 3.86–3.72 (m, 2H), 3.56–3.52 (m, 1H), 3.34 (s, 3H), 2.7–2.65 (m, 1H), 2.45–2.4 (m, 1H), 2.32–2.28 (m, 2H), 2.28–2.2 (m, 3H), 2.14–2.1 (m, 1H), 1.92–1.88 (m, 1H), 1.71 (s, 3H), 1.68–1.55 (m, 2H), 1.48 (s, 3H), 1.4 (s, 3H), 1.2 (s, 3H), 1.12 (d, *J* = 6.3 Hz, 3H), 1.04–0.87 (m, 36H), 0.11–0.06 (m, 18H); ^13^C NMR (125 MHz, CDCl_3_) δ 143.6, 138.1, 134.1, 131.7, 131.4, 129.9, 127.3, 114.3, 107.2, 93.1, 82.1, 81.5, 79.2, 73.2, 72.3, 70.8, 59.9, 55.4, 43.8, 39.7, 39.2, 38.8, 36.3, 36.0, 35.5, 35.4, 29.7, 28.5, 26.6, 26, 25.9, 20.5, 18.7, 18.4, 18, 15.5, 15.4, −4.4, −4.5, −4.8, −5.1.

To a stirred solution of the above TBS ether (115 mg, 0.12 mmol) in methanol (4 mL), we added ammonium fluoride (125 mg, 3.37 mmol) at rt and the reaction mixture was stirred at that temperature for 8 h. Et_2_O (30 mL) was added to precipitate the ammonium fluoride, which was removed by suction filtration. The crude product was purified by flash chromatography (EtOAc/hexane 1:4) to give the alcohol **25** as a colorless oil (94 mg, 95% yield). R_f_ value (EtOAc/hexane 2:1): 0.36; [α]_D_^20^ = +37.4 (*c =* 0.5, CHCl_3_); IR (film, cm^−1^) 3434, 2956, 2931, 1644, 1374, 1253, 1077; ^1^H NMR (500 MHz, CDCl_3_) δ5.77–5.68 (m, 3H), 5.45 (d, *J* = 15.5 Hz, 1H), 5.24 (dd, *J* = 15.5, 8.4 Hz, 1H), 4.94 (s, 1H), 4.85 (s, 1H), 4.70 (d, *J* = 6.8 Hz, 1H), 4.37 (d, *J* = 6.8 Hz, 1H), 4.23 (dd, *J* = 11.6, 8.4 Hz, 1H), 3.9–3.82 (m, 2H), 3.82–3.75 (m, 2H), 3.51–3.49 (m, 1H), 3.29 (s, 3H), 2.85 (brs, 1H), 2.84–2.80 (m, 1H), 2.4–2.35 (m, 1H), 2.3–2.12 (m, 5H), 2.1–2.05 (m, 1H), 1.9–1.84 (m, 2H), 1.71 (s, 3H), 1.65–1.55 (m, 2H), 1.44 (s, 3H), 1.38 (s, 3H), 1.18 (s, 3H), 1.08 (d, *J* = 6.1 Hz, 3H), 0.94–0.82 (m, 27H), 0.1–0.02 (m, 12H); ^13^C NMR (125 MHz, CDCl_3_) δ 143.4, 142.6, 134.0, 132.8, 130.6, 129.8, 126.1, 114.3, 107.1, 92.5, 82.0, 81.3, 77.8, 73.1, 72.2, 70.6, 65.8, 57.0, 55.5, 43.6, 39.5, 38.7, 38.4, 36.2, 36.0, 35.4, 35.3, 28.4, 26.5, 25.8, 20.4, 18.1, 18.0, 15.5, 15.2, −4.5, −4.7, −4.9, −5.1; MS (ESI, *m*/*z*) [M + Na]^+^ 845.5. HRMS (ESI) [M + Na]^+^ calcd for C_46_H_88_O_8_Si_2_Na 847.5916, found 847.5922.

**Macrolactone(26):** To a stirred solution of allyl alcohol **25** (107 mg, 0.13 mmol) in DCM (10 mL), we added activated MnO_2_ (126 mg, 90%, 1.3 mmol) at rt, and the reaction was stirred at rt for 5 h. Suction filtration gave a crude aldehyde (105 mg) that was used for the next step without further purification. R_f_ value (EtOAc/hexane 1:2): 0.73.

To a stirred solution of the above aldehyde (105 mg, 0.12 mmol) and 2-methyl-2-butene (2 mL) in *tert*-butanol (8 mL), we added a solution of NaH_2_PO_4_^.^H_2_O (200 mg) and NaClO_2_ (200 mg), dropwise, in H_2_O (2 mL) at 0 °C. In addition, the reaction mixture was allowed to warm up to rt and stirred for 30 min. The reaction was poured into water (5 mL) and extracted with ethyl acetate (3 × 20 mL). The combined organic extracts were washed with water and brine, dried over anhyd Na_2_SO_4_ and evaporated in vacuo. The crude product was purified by flash chromatography (MeOH/chloroform 3:100) to give the seco-acid as a colorless oil (97 mg, 89% yield for two steps). R_f_ value (EtOAc/hexane 1:2): 0.68.

To the solution of thus obtained seco-acid in THF (4 mL), we added DIPEA (0.33 mL, 1.91 mmol) and 2,4,6-trichlorobenzoyl chloride (0.2 mL, 1.27 mmol) at rt. The reaction was stirred for 3 h at that temperature, before the THF solvent was removed by vacuo. The the residue toluene (10 mL) was added and the solution was transferred to a stirred solution of DMAP (388 mg, 3.18 mmol) in toluene (150 mL) at rt over 16 h, through a syringe pump. The resulting mixture was stirred at rt for 36 h and poured into sat NaHCO_3_ (20 mL). The organic layer was separated and the aq was extracted with diethyl ether (3 × 20 mL). The combined organic phase was washed with water and brine, dried over anhyd MgSO_4_ and concentrated in vacuo. Column chromatography (EtOAc/hexane 1:40) provided macrolactone **26** as a colorless oil (65 mg, 69%). R_f_ value (EtOAc/hexane 1:10): 0.48; [α]_D_^20^ = −18 (*c =* 0.2, CHCl_3_); IR (film, cm^−1^) 2927, 1711, 1155, 1033; ^1^H NMR (500 MHz, CDCl_3_) δ 5.75 (d, *J* = 0.6 Hz, 1H), 5.62–5.55 (m, 2H), 5.48 (d, *J* = 15.6 Hz, 1H), 5.32 (dd, *J* = 15.6, 8.3 Hz, 1H), 4.99 (s, 1H), 4.89 (s, 1H), 4.86–4.88 (m, 1H), 4.67 (d, *J* = 6.8 Hz, 1H), 4.53 (d, *J* = 6.8 Hz, 1H), 4.15–4.10 (m, 1H), 3.96 (dd, *J* = 7.9, 5.8 Hz, 1H), 3.88 (dd, *J* = 7.9, 4.6 Hz, 1H), 3.84–3.79 (m, 1H), 3.79–3.76 (m, 1H), 3.38 (s, 3H), 2.22–2.05 (m, 8H), 1.91–1.85 (m, 1H), 1.9 (d, *J* = 0.6 Hz, 3H), 1.84–1.72 (m, 2H), 1.62–1.56 (m, 1H), 1.48 (s, 3H), 1.40 (s, 3H), 1.22 (s, 3H), 1.12 (d, *J* = 7.2 Hz, 3H), 1.05 (d, *J* = 6.9 Hz, 3H), 0.93–0.78 (m, 24H), 0.07–0.03 (m, 12H); ^13^C NMR (125 MHz, CDCl_3_) δ 166.2, 160, 142.1, 134.9, 130.8, 130.1, 129.6, 118.3, 114.5, 107.2, 93.7, 81.9, 79.9, 79.8, 75.8, 70.9, 69.8, 55.5, 42.8, 40.6, 38.8, 38.3, 37.3, 36.6, 35.8, 35.6, 34.7, 29.7, 28.5, 26.6, 25.8, 24.7, 23.3, 22.7, 21.8, 20.7, 19.5, 18.1, 15.8, 15.1, 14.0, 7.9, −4, −4.4, −4.5, −4.7. MS (ESI, *m*/*z*) [M + Na]^+^ 843.5. HRMS (ESI) [M + Na]^+^ calcd for C_46_H_84_O_8_Si_2_Na 843.5603, found 843.5611.

**Macrolactone alcohol (27):** To a stirred solution of macrolactone **26** (52 mg, 0.063 mmol) in THF (4 mL), we added pyridine (1 mL) followed by HF^.^pyridine complex (70%, 0.5 mL) at 0 °C, and the reaction was warmed up to rt and stirred for 10 h. The reaction mixture was cooled to 0 °C again and sat NaHCO_3_ (20 mL) was added. The organic layer was separated and the aq layer was extracted with diethyl ether (3 × 10 mL). The combined organic phase was washed with sat NaHCO_3_, water and brine, dried over anhyd MgSO_4_ and concentrated in vacuo. Column chromatography (EtOAc/hexane 1:2) provided diol as a colorless oil (33 mg, 89%). R_f_ value (EtOAc/hexane 2:1): 0.55; [α]_D_^20^ = −12 (*c* = 0.18, CHCl_3_); IR (film, cm^−1^) 3456, 2926, 1707, 1155, 1032; ^1^H NMR (500 MHz, CDCl_3_) δ 5.75 (s, 1H), 5.66–5.55 (m, 2H), 5.53 (d, *J* = 15.6 Hz, 1H), 5.44 (dd, *J* = 15.6, 8.3 Hz, 1H), 5.11 (s, 1H), 4.99–4.95 (m, 1H), 4.96 (s, 1H), 4.69 (d, *J* = 6.8 Hz, 1H), 4.55 (d, *J* = 6.8 Hz, 1H), 4.12–4.08 (m, 1H), 4.07–4.03 (m, 2H), 3.87–3.83 (m, 1H), 3.77–3.72 (m, 1H), 3.4 (s, 3H), 2.4 (brs, 1H), 2.36 (dd, *J* = 14.4, 2.8 Hz, 1H), 2.29–2.25 (m, 2H), 2.23–2.2 (m, 2H), 2.14–2.10 (m, 1H), 2.08–2.04 (m, 1H), 1.92 (d, *J* = 1.1 Hz, 3H), 1.9–1.87 (m, 1H), 1.8–1.72 (m, 2H), 1.56–1.52 (m, 1H), 1.49 (s, 3H), 1.40 (s, 3H), 1.22 (s, 3H), 1.17 (d, *J* = 7.0 Hz, 3H), 1.12 (d, *J* = 6.9 Hz, 3H), 0.93 (d, *J* = 6.8 Hz, 3H), 0.90 (d, *J* = 7.0 Hz, 3H); ^13^C NMR (125 MHz, CDCl_3_) δ 166.5, 160.7, 143.5, 134.5, 131.7, 129.9, 129.2, 118.2, 114.8, 107.3, 93.9, 81.9, 80.3, 79.6, 74.9, 69.5, 69.2, 55.6, 43.1, 40.1, 39.1, 37.4, 36.0, 35.9, 35.4, 32.9, 28.5, 26.7, 21.9, 20.2, 20.0, 15.2, 15.1, 14.9.

A solution of the above acetonide (12 mg, 0.020 mmol) in HOAc (0.8 mL) and water (0.2 mL) was heated at 55 °C for 3 h, before the solvents were removed in vacuo. Column chromatography (MeOH/chloroform 1:20) provided alcohol **27** as a colorless oil (11 mg, 81%). R_f_ value (EtOAc/hexane 4:1): 0.22; [α]_D_^20^ = −8 (*c* = 0.2, CHCl_3_); IR (film, cm^−1^) 3500, 2956, 1712, 1034; ^1^H NMR (500 MHz, CDCl_3_) δ 5.75 (s, 1H), 5.78–5.62 (m, 2H), 5.58–5.5 (m, 2H), 5.07 (s, 1H), 5.01 (s, 1H), 4.97–4.93 (m, 1H), 4.69 (d, *J* = 6.8 Hz, 1H), 4.52 (d, *J* = 6.8 Hz, 1H), 4.08 (t, *J* = 6.4 Hz, 1H), 4.01–3.97 (m, 1H), 3.86–3.83 (m, 1H), 3.82–3.76 (m, 1H), 3.72–3.7 (m, 2H), 3.4 (s, 3H), 2.58 (brs, 1H), 2.55 (d, *J* = 14.1 Hz, 1H), 2.42–2.3 (m, 4H), 2.23–1.98 (m, 1H), 1.95–1.9 (m, 1H), 2.05–2.02 (m, 1H), 2–1.95 (m, 1H), 1.98 (d, *J* = 1.1 Hz, 3H), 1.89–1.85 (m, 1H), 1.81–1.75 (m, 1H), 1.5–1.45 (m, 1H), 1.21 (s, 3H), 1.15 (d, *J* = 6.5 Hz, 3H), 1.1 (d, *J* = 6.4 Hz, 3H), 0.98 (d, *J* = 6.8 Hz, 3H), 0.87 (d, *J* = 7.2 Hz, 3H); ^13^C NMR (125 MHz, CDCl_3_) δ 166.5, 163.7, 144, 138.5, 132.4, 128.9, 125.8, 117.5, 116.3, 94.2, 79.1, 74.8, 73.8, 73.2, 69.2, 68.9, 55.9, 41.8, 40.4, 40.5 37.2, 37, 35.5, 35.2, 32.9, 22.5, 21, 19.9, 16.1, 15, 14.1. MS (ESI, *m*/*z*) [M + Na]^+^ 575.3. HRMS (ESI) [M + Na]^+^ calcd for C_31_H_52_O_8_Na 575.3560, found 575.3555.

**TES ether derivative (28):** To a stirred solution of the above diol **27** (7 mg, 0.013 mmol) in DCM (2 mL), we added bromocatechol borane (0.65 mL, 0.1 M soln in DCM, 0.065 mmol) at −78 °C, and the mixture was stirred at that temperature for 1h, before it was quenched with sat NaHCO_3_ (5 mL). The organic layer was separated and the aq layer was extracted with ethyl acetate. The combined organic phase was washed with water and brine, dried over anhyd Na_2_SO_4_ and concentrated in vacuo. Column chromatography (MeOH/chloroform 1:20) provided the corresponding alcohol as a colorless oil (4.8 mg, 72%). R_f_ value (EtOAc/hexane/MeOH 8:2:1): 0.33; [α]_D_^20^ = −21 (*c* = 0.074, CHCl_3_); IR (film, cm^−1^) 3430, 2956, 1706; ^1^H NMR (500 MHz, CDCl_3_) δ 5.9–5.85 (m, 1H), 5.84–5.78 (m, 1H), 5.82 (s, 1H), 5.75–5.69 (m, 1H), 5.58 (d, *J* = 15.8 Hz, 1H), 5.05 (s, 1H), 5.05–5.02 (m, 1H), 5.02 (s, 1H), 3.96–3.92 (m, 1H), 3.81–3.73 (m, 2H), 3.62–3.58 (m, 2H), 2.65–2.55 (m, 2H), 2.5–2.44 (m, 1H), 2.42–2.38 (m, 2H), 2.18–2.1 (m, 2H), 2.02–1.98 (m, 1H), 1.92 (s, 3H), 1.7–1.64 (m, 2H), 1.43–1.40 (m, 1H), 1.19 (s, 3H), 1.07 (d, *J* = 6.9 Hz, 3H), 1.05 (d, *J* = 7.1 Hz, 3H), 0.95 (d, *J* = 6.6 Hz, 3H), 0.88 (d, *J* = 6.9 Hz, 3H).

To a stirred solution of the above alcohol (3.2 mg, 0.006 mmol) and DMAP (18 mg, 0.14 mmol) in DCM (2 mL), we added TESCl (16 µL, 0.1 mmol) at 0 °C, and the reaction was stirred at 0 °C for 30 min, before sat NaHCO_3_ (5 mL) was added. The organic layer was separated and the aq layer was extracted with diethyl ether (3 × 10 mL). The combined organic phase was washed with water and brine, dried over anhyd MgSO_4_ and concentrated in vacuo. Column chromatography (EtOAc/hexane 1:20) provided *tris*-TES ether **28** as a colorless oil (4.9 mg, 92%). R_f_ value (EtOAc/hexane 1:4): 0.5; [α]_D_^20^ = +11 (*c* = 0.22, CHCl_3_); IR (film, cm^−1^) 3430, 2896, 1079; ^1^H NMR (500 MHz, CDCl_3_) δ 5.84-5.78 (m, 1H), 5.64 -5.60 (m, 3H), 5.42 (d, *J* = 15.5 Hz, 1H), 4.91 (brs, 2H), 4.76 (dt, *J* = 5.0, 2.5 Hz, 1H), 4.1–4.06 (m, 1H), 4.02–3.98 (m, 1H), 3.88–3.84 (m, 1H), 3.75–3.70 (m, 1H), 3.6–3.56 (m, 1H), 3.52 (brs, 1H), 2.53–2.49 (m, 1H), 2.4–2.36 (m, 2H), 2.3–2.25 (m, 1H), 2.18–2.08 (m, 2H), 2.07–2.01 (m, 4H), 1.85 (brs, 3H), 1.85–1.8 (m, 1H), 1.4–1.35 (m, 1H), 1.18 (s, 3H), 1.11 (d, *J* = 6.4 Hz, 3H), 0.96–0.9 (m, 33H), 0.8 (d, *J* = 6.9 Hz, 3H), 0.6–0.53 (m, 18H); MS (ESI, *m*/*z*) [M+Na]^+^ 873.33. HRMS (ESI) [M + Na]^+^ calcd for C_47_H_90_O_7_Si_3_Na 873.5892, found 873.5888.

**Iriomoteolide-1a (1)**: To a stirred solution of diol **28** (3 mg, 3.5 μmol) in DCM (1 mL), we added Dess–Martin periodinane (0.12 mL, 0.3 M soln in DCM, 0.035 mmol) at rt, and the reaction was stirred at rt for 1 h. Direct column chromatography (EtOAc/hexane 1:15) provided the corresponding ketone as a colorless oil (1.9 mg, 65%, 90% BRSM), along with the recovered starting material (1 mg). R_f_ value (EtOAc/hexane 1:4): 0.75; ^1^H NMR (500 MHz, CDCl_3_) δ 5.85–5.80 (m, 1H), 5.63 (s, 1H), 5.61–5.55 (m, 1H), 5.46 (d, *J* = 15.6 Hz, 1H), 5.37 (dd, *J* = 15.6, 8.0 Hz, 1H), 5.01 (s, 1H), 4.91–4.87 (m, 1H), 4.85 (s, 1H), 4.18–4.13 (m, 2H), 4.08 (s, 1H), 3.79–3.75 (m, 1H), 3.69–3.66 (m, 1H), 3.42 (d, *J* = 15.0 Hz, 1H), 3.12 (d, *J* = 15.0 Hz, 1H), 2.2–2.16 (m, 2H), 2.1–2 (m, 4H), 1.97–1.92 (m, 2H), 1.87 (brs, 3H), 1.85–1.81 (m, 1H), 1.46 (s, 3H), 1.1 (d, *J* = 6.4 Hz, 3H), 1.04 (d, *J* = 6.6 Hz, 3H), 0.98–0.92 (m, 30H), 0.85 (d, *J* = 7.0 Hz, 3H), 0.61–0.54 (m, 18H). To a stirred solution of the above ketone (3.0 mg, 0.0035 mmol) in THF (0.6 mL), we added a HF·Py solution (0.1 mL containing 1 mL 70% HF·Py: 1.1 mL pyridine: 2.4 mL THF) at rt. After 1 h, the reaction was quenched with sat NaHCO_3_ and extracted with diethyl ether (3 × 10 mL). The combined organic layer was washed with water and brine, dried over MgSO_4_ and concentrated under reduced pressure. The resulting residue was purified by flash chromatography (MeOH/chloroform 1:25) to give iriomoteolide-1a (**1**) (1.0 mg, 56%) as a colorless oil, along with the isomerized product, iriomoteolide-1b (**2**) (0.3 mg, 17%), as a colorless oil.

**Iriomoteolide-1a (1)** R_f_ value (EtOAc/hexane 2:1): 0.4. [α]_D_^20^ = −12.0 (*c =* 0.10, CHCl_3_); IR (film, cm^−1^) 3456, 2926, 1707, 1032; ^1^H NMR (500 MHz, CDCl_3_) δ5.9–5.88 (m, 1H), 5.88–5.83 (m, 1H), 5.83–5.8 (m, 1H), 5.78 (s, 1H), 5.68 (dd, *J* = 15.4, 6.8 Hz, 1H), 5.05–4.99 (m, 1H), 4.88 (d, *J* = 1.6 Hz, 1H), 4.86 (d, *J* = 1.4 Hz, 1H), 4.07 (dd, *J*_1_ = *J*_2_ = 6.8 Hz, 1H), 4.03–3.97 (m, 1H), 4–3.95 (m, 1H), 3.89–3.83 (m, 1H), 3.29 (brs, 1H), 2.67 (brs, 1H), 2.37–2.3 (m, 1H), 2.32–2.28 (m, 1H), 2.25–2.2 (m, 3H), 2.18–2.13 (m, 2H), 2.05–2 (m, 1H), 1.96 (s, 3H), 2–1.92 (m, 1H), 1.84–1.78 (m, 1H), 1.56–1.5 (m, 1H), 1.4–1.35 (m, 1H), 1.33 (s, 3H), 1.12 (d, *J* = 6.4 Hz, 3H), 1.1 (d, *J* = 7 Hz, 3H), 1.01 (d, *J* = 7 Hz, 3H), 0.9 (d, *J* = 6.9 Hz, 3H); ^13^C NMR (125 MHz, CDCl_3_) δ 166.3, 160, 141.7, 134.8, 132.7, 129.6, 127.2, 118.8, 110.8, 99.1, 77.2, 74.9, 74.4, 70.3, 69.3, 40.6, 39.7, 37.8, 37.3, 36.7, 35.5, 34.2, 20.8, 20.6, 20, 15.8, 15.7, 14.9; MS (ESI, *m*/*z*) [M + Na]^+^ 529.24. HRMS (ESI) [M + Na]^+^ calcd for C_29_H_46_O_7_Na 529.3141, found 529.3139.

**Iriomoteolide-1b (2)** R_f_ value (EtOAc/hexane 2:1): 0.32; [α]_D_^20^ = −78 (*c =* 0.03, CHCl_3_); IR (film, cm^−1^) 3703, 2965, 1810, 1694; ^1^H NMR (500 MHz, CDCl_3_) δ 6.32 (s, 1H), 5.82 (s, 1H), 5.83–5.78 (m, 1H), 5.73–5.68 (m, 2H), 5.54 (d, *J* = 15.7 Hz, 1H), 4.98 (dt, *J* = 7.5, 3.0 Hz, 1H), 4.55 (s, 1H), 4.17–4.1 (m, 1H), 3.90 (m, 1H), 3.82–3.75 (m, 1H), 3.75–3.7 (m, 1H), 2.35–2.3 (m, 2H), 2.3–2.22 (m, 2H), 2.27 (s, 3H), 2.12–2.08 (m, 2H), 1.99 (s, 3H), 1.98–1.9 (m, 2H), 1.66–1.6 (m, 1H), 1.48 (s, 3H), 1.42–1.35 (m, 1H), 1.18 (d, *J* = 7.1 Hz, 3H), 1.12 (d, *J* = 6.8 Hz, 3H), 0.92 (d, *J* = 7.0 Hz, 3H), 0.89 (d, *J* = 6.4 Hz, 3H); ^13^C NMR (125 MHz, CDCl_3_) δ 200.7, 167.2, 160.1, 159.3, 136, 132.9, 129.8, 126.5, 120.5, 118.6, 77.6, 76.9, 75.5, 69.4, 68.2, 48.3, 40.9, 40.4, 36.2, 35.6, 31.5, 30.9, 22.6, 21.6, 20.3, 20.1, 15.7, 15, 13.7; MS (ESI, *m*/*z*) [M + Na]^+^ 529.33. HRMS (ESI) [M + Na]^+^ calcd for C_29_H_46_O_7_Na 529.3141, found 529.3135.

**Alcohol (32):** To a suspension of CuI (2.18 g, 11.4 mmol) in THF (30 mL), we added MeLi (14.3 mL, 22.8 mmol) at −60 °C. The mixture was slowly warmed up to 0 °C to obtain a clear solution and cooled back to −60 °C. TMSCl (1.5 mL, 11.4 mmol) was added dropwise and stirred at this temperature for 5 min. A soln of alkynyl ester **21** (1.2 g, 5.7 mmol) in THF (5 mL) was added dropwise at −60 °C. After addtion, the reaction mixture was warmed up to 0 °C slowly and stirred for 30 min. Then, the reaction mixture was poured into sat NH_4_Cl (30 mL) and crushed ice. The organic layer was separated and the aqueous layer was extracted by Et_2_O (3 × 20 mL). The combined organic extracts were washed with water and brine, dried over anhydrous MgSO_4_, filtered, and concentrated in vacuo. Flash chromatography on silica gel (5% EtOAc/hexanes) produced an inseparable mixture of *E*-enoate and *Z*-isomer (1.2 g, 92%) as a colorless oil. R_f_ value (EtOAc/hexane 1:10) 0.45.

To the above stirred enoate (1.15 g, 5.04 mmol) in DCM (50 mL), we added DIBAL-H (20 mL, 20 mmol) at −78 °C. The reaction mixture was stirred 1h before addition of 10 mL saturated NH_4_Cl (aq) and 20 mL 25% Rochelle salt solution. The mixture was stirred at rt, until a clear soln was obtained. The organic phase was separated and the aq phase was extracted with Et_2_O (3 × 30 mL). The combined organic extracts were washed with water and brine, dried over anhydrous Na_2_SO_4_, filtered, and concentrated in vacuo. Flash chromatography on silica gel (15% Et_2_O/hexanes) afforded the *E*-allyl alcohol **32** (0.71 g, 68%) and *Z*-isomer (0.3 g, 29%). R_f_ value (EtOAc/hexane 1:4) 0.2; [α]_D_^20^ = +27 (*c =* 0.2, CHCl_3_); IR (film, cm^−1^): 3350, 2965, 1624, 1456, 1210, 1008, 914; ^1^H NMR (400 MHz, CDCl_3_) δ 5.55 (ddd, *J* = 17.2, 10.3, 8.1 Hz, 1H), 5.44 (t, *J* = 6.7 Hz, 1H), 5.26–5.09 (m, 2H), 4.62 (d, *J* = 6.9 Hz, 1H), 4.41 (d, *J* = 6.9 Hz, 1H), 4.11 (d, *J* = 6.7 Hz, 2H), 3.86 (t, *J* = 8.2 Hz, 1H), 3.27 (s, 3H), 2.35–2.16 (m, 1H), 2.10 (s, 1H), 1.63 (s, 3H), 0.92 (d, *J* = 7.1 Hz, 3H). ^13^C NMR (100 MHz, CDCl_3_) δ 140.28, 136.49, 125.11, 118.63, 93.38, 79.84, 77.19 58.99, 55.37, 46.73, 15.5, 13.36. MS (ESI, *m*/*z*) [M+Na]^+^ 223.1.

**Aldehyde (33):** To a stirred solution of the above alcohol (0.7 g, 3.5 mmol) (1.45 g, 7.24 mmol) in DCM (30 mL), we added imidazole (357 mg, 5.26 mmol) and TBSCl (0.58 g, 3.82 mmol) at 0 °C. The reaction was warmed up to rt and stirred for 1 h, before pouring into a mixture of sat NaHCO_3_ (50 mL) and crushed ice. The mixture was extracted with ethers (3 × 30 mL) and the organic layer was washed with water and brine, dried over anhyd MgSO_4_ and evaporated in vacuo to give the crude TBS ether as a clear oil. The resulting crude product was used for the next step without further purification. Flash chromatography on silica gel (25% EtOAc/hexanes) afforded the silyl ether (1.1 g, 100%). R_f_ value (EtOAc/hexane 1:4) 0.8; [α]_D_^20^ = +12 (*c =* 0.55, CHCl_3_); IR (film, cm^−1^): 2970, 2811, 1610, 1230, 1155, 925; 1H NMR (400 MHz, CDCl3) δ 5.59 (ddd, *J* = 17.2, 10.4, 8.0 Hz, 1H), 5.39 (t, *J* = 6.0 Hz, 1H), 5.26–5.10 (m, 2H), 4.65 (d, *J* = 6.8 Hz, 1H), 4.44 (d, *J* = 6.8 Hz, 1H), 4.20 (d, *J* = 6.1 Hz, 2H), 3.89 (t, *J* = 8.1 Hz, 1H), 3.30 (s, 3H), 2.36–2.16 (m, 1H), 1.62 (d, *J* = 0.7 Hz, 3H), 0.95 (d, *J* = 7.1 Hz, 3H), 0.88 (s, 9H), 0.04 (s, 6H). ^13^C NMR (101 MHz, CDCl_3_) δ 137.81, 136.65, 126.04, 118.29, 93.45, 79.81, 60.09, 55.33, 46.64, 25.86, 18.24, 15.29, 13.66, −5.20.

To a stirred solution of the above olefin (1.05 g, 3.34 mmol) (1.09 g, 3.47 mmol) in dioxane (20 mL) and water (7 mL), we added 2,6-lutidine (1.9 mL, 16.7 mmol), OsO_4_ (2.5% in *t*-BuOH, 1.6 mL, 0.13 mmol) and NaIO_4_ (2.85 g, 12.4 mmol) at 0 °C. The mixture was stirred at 0 °C for 16 h before saturated NaHCO_3_ (10 mL) and Na_2_S_2_O_3_ (10 mL) were added. The mixture was stirred for another 30 min, extracted by EtOAc. The combined organic extracts were dried over anhydrous Na_2_SO_4_, filtered, and concentrated in vacuo. Flash chromatography on silica gel (25% EtOAc/hexanes) produced the aldehyde **33** (655 mg, 62%), which was used for the next step quickly.

**Aldehyde (34):** To a stirred solution of sulfone **5** (628 mg, 0.94 mmol) in DME (30 mL), we added KHMDS (1.85 mL, 0.5 M soln in toluene, 0.93 mmol) at −78 °C. The reaction mixture was stirred for 30 min, before a soln of aldehyde **33** (357 mg, 1.13 mmol) in DME (5 mL) was transferred in. The reaction mixture was stirred for another 30 min, before it was warmed up to rt. The reaction was quenched by sat NH_4_Cl (10 mL) at −78 °C. The organic layer was separated and the aq layer was extracted with diethyl ether (3 × 20 mL). The combined organic extracts were washed with water and brine, dried on anhyd MgSO_4_ and evaporated in vacuo. The crude product was purified by flash chromatography (EtOAc/hexane 1:30) to give olefin as a colorless oil (544 mg, 76% yield). R_f_ value (EtOAc/hexane 1:10): 0.5; [α]_D_^20^ = +14 (*c* = 0.6, CHCl_3_); IR (film, cm^−1^) 2928, 1455, 1248, 1108; ^1^H NMR (400 MHz, CDCl_3_) δ 5.73–5.54 (m, 1H), 5.40 (t, *J* = 6.1 Hz, 1H), 5.23 (dd, *J* = 15.4, 8.5 Hz, 1H), 4.88 (d, *J* = 32.2 Hz, 2H), 4.68 (d, *J* = 6.8 Hz, 1H), 4.56 (d, *J* = 5.1 Hz, 2H), 4.41 (d, *J* = 6.8 Hz, 1H), 4.21 (d, *J* = 6.1 Hz, 3H), 4.14 (dd, *J* = 8.7, 3.7 Hz, 1H), 3.86 (t, *J* = 8.3 Hz, 2H), 3.41 (q, *J* = 9.8 Hz, 2H), 3.30 (s, 3H), 2.35–2.09 (m, 8H), 1.63 (s, 3H), 1.44 (s, 3H), 1.35 (s, 3H), 1.13 (s, 3H), 0.95 (d, *J* = 7.0 Hz, 3H), 0.9 (s, 9H), 0.87 (s, 9H), 0.12–−0.07 (m, 12H); ^13^C NMR (100 MHz, CDCl_3_) δ 143.50, 138.19, 131.28, 130.98, 128.21, 127.41, 127.29, 125.83, 114.25, 107.15, 93.11, 81.53, 79.29, 78.34, 74.98, 73.45, 70.52, 60.11, 55.31, 46.97, 43.68, 39.56, 36.49, 28.56, 26.44, 25.90, 25.80, 19.16, 18.27, 17.97, 15.76, 13.52, −4.59, −4.65, −5.15.

To a stirred solution of the above benzyl ether (520 mg, 0.68 mmol) in THF (20 mL) and allyl ethyl ether (1 mL), we transferred a soln of lithium metal (100 mg, 14.4 mmol) in liquid ammonia (30 mL) in portions at −78 °C. The reaction was carefully monitored by TLC and stopped immediately after the solution became slightly blue. Ammonium chloride (3 g) was added to quench the reaction. The mixture was allowed to warm up to rt to evaporate ammonia, before water (10 mL) was added. The organic layer was separated and the aq layer was extracted with diethyl ether (3 × 20 mL). The combined organic extracts were washed with water and brine, dried over anhyd MgSO_4_ and evaporated in vacuo. The crude product was purified by flash chromatography (EtOAc/hexane 1:10) to give the alcohol as a colorless oil (356 mg, 78% yield), along with the recovered starting material. R_f_ value (EtOAc/hexane 1:4): 0.4; [α]_D_^20^ = +12 (*c =* 1, CHCl_3_); IR (film, cm^−1^) 3430, 2965, 1255, 1087; ^1^H NMR (400 MHz, CDCl_3_) δ 5.70–5.55 (m, 1H), 5.38 (t, *J* = 6.0 Hz, 1H), 5.23 (dd, *J* = 15.5, 8.4 Hz, 1H), 4.93 (s, 1H), 4.85 (s, 1H), 4.67 (d, *J* = 6.8 Hz, 1H), 4.40 (d, *J* = 6.8 Hz, 1H), 4.19 (d, *J* = 5.6 Hz, 3H), 3.85 (dd, *J* = 11.1, 5.3 Hz, 2H), 3.51 (dd, *J* = 11.8, 3.6 Hz, 1H), 3.34 (dd, *J* = 11.5, 9.5 Hz, 1H), 3.28 (s, 3H), 2.37–2.03 (m, 10H), 1.60 (s, 3H), 1.43 (s, 3H), 1.34 (s, 3H), 1.04 (s, 3H), 0.97–0.91 (m, 3H), 0.87 (s, 9H), 0.86 (s, 9H), 0.04 (s, 12H); ^13^C NMR (100 MHz, CDCl_3_) δ 143, 138.15, 131.21, 131.01, 125.82, 114.54, 106.99, 93.07, 82.56, 79.24, 75.94, 70.70, 65.40, 60.10, 55.29, 46.92, 43.44, 39.63, 36.07, 28.59, 26.60, 25.88, 25.78, 18.60, 18.25, 17.95, 15.70, 13.52, −4.62, −4.66, −5.17.

To a suspension of the above alcohol (139 mg, 0.21 mmol) and sodium bicarbonate (106 mg, 1.26 mmol) in DCM (15 mL), we added Dess–Martin periodinane (176 mg, 0.42 mmol) at rt. The reaction mixture was stirred for 1 h, before it was poured into a mixture of sat NaHCO_3_ (10 mL) and sodium thiosulfate (10 mL). The organic layer was separated and the aq layer was extracted with diethyl ether (3 × 20 mL). The combined organic extracts were washed with water and brine, dried on anhyd MgSO_4_ and evaporated in vacuo to give the crude aldehyde **34** (138 mg, quantitative), which was used directly in the next step without further purification.

**Alcohol (36)**: To a stirred mixture of potassium *tert*-butoxide (13.9 mL, 1.0 M soln in THF, 13.9 mmol) and *trans*-2-butene (2.2 mL, 23.2 mmol) in THF (40 mL), we added *n*-butyllithium (8.8 mL, 1.6 M soln in THF, 13.9 mmol) at −78 °C. After complete addition of *n*-butyllithium, the mixture was stirred at −45 °C for 10 min. The resulting orange solution was cooled to −78 °C again and a solution of (−)-Ipc_2_BOMe (5.3 g, 16.6 mmol) in THF (10 mL) was added dropwise. After 30 min of stirring, boron trifluoride etherate (2.4 mL, 18.6 mmol) was added dropwise. Then, the aldehyde **35** (2.14 g, 9.3 mmol) in THF (5 mL) was transferred in. The mixture was stirred at −78 °C for 3 h, before NaOH (11 mL, 3 M soln) and H_2_O_2_ (7.5 mL, 70% soln) were added. The contents were refluxed for 1 h. The organic layer was separated and the aq layer was extracted with diethyl ether (3 × 30 mL). The combined organic extracts were washed with water and brine, dried over anhyd MgSO_4_ and evaporated in vacuo. The crude product was purified by flash chromatography (EtOAc/hexane 1:20) to give the alcohol **36**-favored desired *syn* product (8:1 dr) as a colorless oil (1.92 g, 72% yield). R_f_ value (EtOAc/hexane 1:10): 0.6; [α]_D_^20^ = +1.5 (*c* = 0.33, CHCl_3_); IR (film, cm^−1^) 3410, 2965, 1456, 1044; ^1^H NMR (500 MHz, CDCl_3_) δ 5.81 (ddd, *J* = 17.8, 10.4, 7.6 Hz, 1H), 5.07–5.05 (m, 1H), 5.04–5.01 (m, 1H), 5.02–5.00 (m, 1H), 3.76 (qd, *J* = 6.5, 3.5 Hz, 1H), 3.46 (dd, *J* = 8.5, 3.6 Hz, 1H), 3.24 (d, *J* = 3.6 Hz, 1H), 2.30–2.21 (m, 1H), 1.80–1.69 (m, 1H), 1.48–1.30 (m, 3H), 1.08 (d, *J* = 6.3 Hz, 3H), 1.03 (d, *J* = 6.9 Hz, 3H), 0.88 (s, 9H), 0.84 (d, *J* = 7.0 Hz, 3H), 0.06 (s, 6H); ^13^C NMR (125 MHz, CDCl_3_) δ 141.36, 114.43, 73.75, 72.83, 44.16, 38.41, 36.93, 18.30, 17.99, 16.98, 15.22, −4.72, −5.09; MS (ESI, *m*/*z*) [M + Na]^+^ 309.

**Alcohol (37)**: To a stirred solution of alcohol **36** (1.78 g, 6.25 mmol) in DMF (25 mL), we added NaH (60%, 375 mg, 9.25 mmol) at 0 °C. The mixture was stirred for 30 min before PMBCl (1.25 mL, 9.25 mmol) was added at 0 °C. After stirring at rt over night, water (10 mL) and Et_2_NH (5 mL) were added and the mixture was stirred for 1h, before it was poured into sat NaHCO_3_ (aq). The mixture was extracted with diethyl ether (3 × 30 mL). The combined organic extracts were washed with water and brine, dried over anhyd MgSO_4_ and evaporated in vacuo. The crude product was purified by flash chromatography (EtOAc/hexane 1:30) to give the PMB ether as a colorless oil (2.11 g, 83% yield). R_f_ value (EtOAc/hexane 1:10): 0.7; [α]_D_^20^ = +3.2 (*c =* 0.35, CHCl_3_); IR (film, cm^−1^) 2952, 1654, 1062; ^1^H NMR (500 MHz, CDCl_3_) δ 7.27 (d, *J* = 8.6 Hz, 1H), 6.87 (d, *J* = 8.6 Hz, 1H), 5.94–5.83 (m, 1H), 5.09–4.99 (m, 1H), 4.56 (d, *J* = 11.0 Hz, 1H), 4.39 (d, *J* = 11.0 Hz, 1H), 3.81 (s, 1H), 3.67 (tt, *J* = 9.7, 4.9 Hz, 1H), 3.37 (ddd, *J* = 10.2, 5.0, 2.4 Hz, 1H), 2.60–2.52 (m, 1H), 1.65 (tt, *J* = 13.5, 5.0 Hz, 1H), 1.51 (ddd, *J* = 13.4, 10.3, 2.8 Hz, 1H), 1.27 (ddd, *J* = 13.7, 10.9, 2.6 Hz, 1H), 1.07 (d, *J* = 6.2 Hz, 3H), 1.03 (d, *J* = 6.9 Hz, 3H), 0.88 (brs, 9H), 0.79 (d, *J* = 6.7 Hz, 3H), 0.02 (m, 6H); ^13^C NMR (125 MHz, CDCl_3_) δ 158.93, 140.66, 131.08, 129.18, 114.21, 113.57, 80.37, 72.47, 71.02, 55.16, 40.42, 36.03, 35.42, 25.79, 20.94, 18.01, 15.45, 12.96, −4.35, −4.91.

To a stirred solution of the above olefin (1.89 g, 4.68 mmol) in THF (30 mL), we added 9-BBN (18.5 mL, 0.5 M soln in THF, 9.36 mmol) at 0 °C. The reaction mixture was allowed to warm up to rt and stirred for 3 h. NaOH (1 mL) and H_2_O_2_ (5.5 mL) were added and the mixture was refluxed for 1 h. The organic layer was separated and the aq layer was extracted with Et_2_O; the combined organic layer was washed with water and brine, dried over Na_2_SO_4_ and concentrated in vacuo. Column chromatography provided alcohol **37** as a colorless oil (1.55 g, 77%). R_f_ value (EtOAc/hexane 1:1): 0.65; [α]_D_^20^ = +1.5 (*c =* 0.2, CHCl_3_); IR (film, cm^−1^) 3411, 2926, 1609, 1038; ^1^H NMR (400 MHz, CDCl_3_) δ 7.24 (d, *J* = 8.5 Hz, 1H), 6.86 (d, *J* = 8.5 Hz, 1H), 4.56 (d, *J* = 11.0 Hz, 1H), 4.38 (d, *J* = 11.0 Hz, 1H), 3.78 (d, *J* = 2.1 Hz, 2H), 3.73–3.63 (m, 1H), 3.59 (d, *J* = 12.3 Hz, 1H), 3.36 (d, *J* = 10.1 Hz, 1H), 2.93 (s, 1H), 2.05 (dd, *J* = 7.9, 5.6 Hz, 1H), 1.76 (dt, *J* = 21.2, 7.1 Hz, 1H), 1.69–1.51 (m, 1H), 1.45–1.31 (m, 1H), 1.18 (dd, *J* = 13.4, 10.9 Hz, 1H), 1.07 (d, *J* = 6.2 Hz, 3H), 0.88 (d, *J* = 8.1 Hz, 3H), 0.87 (s, 9H), 0.76 (d, *J* = 6.7 Hz, 3H), 0.02 (s, 3H), 0.01 (s, 3H); ^13^C NMR (100 MHz, CDCl_3_) δ 159.05, 130.55, 129.32, 113.63, 80.86, 72.38, 71.19, 61.80, 55.12, 36.18, 34.81, 33.45, 33.11, 25.80, 20.75, 18.01, 17.32, 13.21, −4.34, −4.86. MS (ESI, *m*/*z*) [M + Na]^+^ 447.4.

**Sulfone (38)**: To a stirred solution of alcohol **37** (1.5 g, 3.57 mmol), 1-(4-Hydroxyphenyl)-1*H*-tetrazole-5-thiol (1.26 g, 6.97 mmol) and triphenylphosphine (1.38 g, 5.29 mmol) in THF (30 mL), we added DIAD (1.2 mL, 6.29 mmol) at 0 °C. The reaction mixture was warmed up to rt and stirred overnight, before it was poured into sat NaHCO_3_ (30 mL). The organic layer was separated and the aq layer was extracted with Et_2_O; the combined organic layer was washed with water and brine, dried over MgSO_4_ and concentrated in vacuo. Column chromatography provided the sulfide as a colorless oil (1.7 g, 83%). R_f_ value (EtOAc/hexane 1:4): 0.5; [α]_D_^20^ = +2.2 (*c =* 0.25, CHCl_3_); IR (film, cm^−1^) 2962, 2878, 1616, 1069; ^1^H NMR (500 MHz, CDCl_3_) δ 7.58–7.51 (m, 1H), 7.22 (d, *J* = 8.7 Hz, 1H), 6.84 (d, *J* = 8.7 Hz, 1H), 4.50 (d, *J* = 11.1 Hz, 1H), 4.36 (d, *J* = 11.1 Hz, 1H), 3.77 (s, 1H), 3.68 (qd, *J* = 6.1, 3.8 Hz, 1H), 3.53 (ddd, *J* = 12.9, 8.9, 5.6 Hz, 1H), 3.41–3.32 (m, 1H), 2.09–2.00 (m, 1H), 1.63 (ddt, *J* = 10.5, 8.9, 4.4 Hz, 1H), 1.54 (ddd, *J* = 13.3, 10.0, 3.0 Hz, 1H), 1.19 (ddd, *J* = 13.6, 10.5, 2.8 Hz, 1H), 1.06 (d, *J* = 6.2 Hz, 3H), 0.93 (d, *J* = 6.8 Hz, 3H), 0.87 (s, 9H), 0.78 (d, *J* = 6.7 Hz, 3H), 0.01 (s, *J* = 8.2 Hz, 6H); ^13^C NMR (125 MHz, CDCl_3_) δ 158.90, 154.35, 133.63, 130.92, 129.92, 129.64, 129.09, 123.71, 113.57, 80.20, 72.31, 71.08, 55.15, 36.20, 34.40, 33.69, 31.92, 30.96, 25.80, 20.68, 18.02, 15.52, 13.39, −4.32, −4.84.

To a stirred solution of thus obtained sulfide (1.41 g, 2.28 mmol) in ethanol (35 mL), we added a solution of ammonium molybdate (1.28 g, 1.04 mmol) in hydrogen peroxide (6.5 mL) and water (3 mL) at rt. The reaction mixture was stirred for 12 h and poured into a mixture of sat NaHCO_3_ (10 mL) and sodium thiosulfate (10 mL). The organic layer was separated and the aq layer was extracted with diethyl ether (3 × 20 mL). The combined organic extracts were washed with water and brine, dried on anhyd MgSO_4_ and evaporated in vacuo. The crude product was purified by flash chromatography (EtOAc/hexane 1:10) to give sulfone **38** as a colorless oil (1.4 g, 94% yield). R_f_ value (EtOAc/hexane 1:4): 0.5; [α]_D_^20^ = +0.6 (*c =* 0.3, CHCl_3_); IR (film, cm^−1^) 2928, 2860, 1609, 1511, 1252, 1068; ^1^H NMR (500 MHz, CDCl_3_) δ 7.70–7.65 (m, 1H), 7.63–7.56 (m, 2H), 7.24 (d, *J* = 8.5 Hz, 1H), 6.87 (d, *J* = 8.4 Hz, 1H), 4.49 (d, *J* = 11.1 Hz, 1H), 4.39 (d, *J* = 11.1 Hz, 1H), 3.80 (s, 3H), 3.68 (tt, *J* = 6.3, 3.1 Hz, 1H), 3.39 (dt, *J* = 9.8, 3.0 Hz, 1H), 2.21–2.11 (m, 1H), 2.07 (ddd, *J* = 9.7, 7.4, 3.2 Hz, 1H), 1.74 (ddd, *J* = 12.0, 9.2, 7.3 Hz, 1H), 1.63 (dtd, *J* = 9.7, 6.5, 3.1 Hz, 1H), 1.56 (ddd, *J* = 13.2, 9.9, 3.0 Hz, 1H), 1.18 (ddd, *J* = 13.5, 10.6, 3.0 Hz, 1H), 1.07 (d, *J* = 6.2 Hz, 3H), 0.93 (d, *J* = 6.9 Hz, 3H), 0.87 (s, 9H), 0.78 (d, *J* = 6.7 Hz, 3H), 0.03 (s, 3H), 0.01 (s, 3H); ^13^C NMR (125 MHz, CDCl_3_) δ 159.01, 153.29, 132.95, 131.29, 130.62, 129.57, 129.26, 124.99, 113.67, 79.96, 72.23, 71.19, 55.18, 54.71, 36.20, 34.18, 33.41, 25.78, 24.00, 20.63, 18.01, 15.65, 13.43, −4.33, −4.86.

**Coupling product (39):** To a stirred solution of the above solfone (194 mg, 0.32 mmol) and aldehyde **34** (253 mg, 0.38 mmol) in DME (15 mL), we added KHMDS (1 mL, 0.5 M soln in toluene, 0.5 mmol) at −65 °C. The reaction mixture was stirred for 1 h at −65 °C before it was warmed up to rt. The reaction was quenched by sat NH_4_Cl (5 mL) at −65 °C and the organic layer was separated and the aq layer was extracted with diethyl ether (3 × 20 mL). The combined organic extracts were washed with water and brine, dried on anhyd MgSO_4_ and evaporated in vacuo. The crude product was purified by flash chromatography (EtOAc/hexane 1:20) to give the pure *E*-olefin **39** as a colorless oil (180 mg, 54% yield) and corresponding *Z*-isomer (21 mg, 6%). R_f_ value (EtOAc/hexane 1:10): 0.45; [α]_D_^20^ = +11 (*c =* 0.35, CHCl_3_); IR (film, cm^−1^) 2861, 1735, 1622, 1515, 1263, 1080; ^1^H NMR (500 MHz, CDCl_3_) δ 7.78 (d, *J* = 8.3 Hz, 1H), 7.39 (d, *J* = 8.4 Hz, 1H), 6.24–6.13 (m, 1H), 5.97 (d, *J* = 15.6 Hz, 1H), 5.93 (t, *J* = 6.1 Hz, 1H), 5.78 (dd, *J* = 15.4, 8.5 Hz, 1H), 5.42 (d, *J* = 39.9 Hz, 1H), 5.22 (d, *J* = 6.8 Hz, 1H), 5.02 (d, *J* = 11.1 Hz, 1H), 4.93 (dd, *J* = 20.6, 9.0 Hz, 1H), 4.74 (d, *J* = 6.0 Hz, 1H), 4.40 (t, *J* = 8.5 Hz, 2H), 4.33 (s, 3H), 4.25–4.17 (m, 1H), 3.88 (d, *J* = 5.5 Hz, 1H), 3.84 (s, 3H), 2.91–2.75 (m, 2H), 2.72 (dd, *J* = 12.4, 6.9 Hz, 2H), 2.63 (d, *J* = 12.9 Hz, 1H), 2.42–2.31 (m, 1H), 2.16 (s, 3H), 1.99 (s, 3H), 1.90 (s, 3H), 1.60 (d, *J* = 6.2 Hz, 3H), 1.49 (d, *J* = 7.0 Hz, 3H), 1.42 (m, 27H), 1.32 (d, *J* = 6.6 Hz, 3H), 0.58–0.53 (m, 18H).

To a stirred solution of the above PMB ether (163 mg, 0.16 mmol) in DCM (10 mL) and pH 7.0 buffer (0.8 mL), we added DDQ (70 mg, 0.32 mmol) at 0 °C and the reaction mixture was stirred at that temperature for 1 h. The reaction was quenched by sat NaHCO_3_ (5 mL) and the organic layer was separated. The aq layer was extracted with diethyl ether (3 × 20 mL). The combined organic extracts were washed with sat NaHCO_3_, water and brine, dried on anhyd MgSO_4_ and evaporated in vacuo. The crude product was purified by flash chromatography (EtOAc/hexane 1:15) to give the corresponding alcohol as a colorless oil (119 mg, 79% yield). R_f_ value (EtOAc/hexane 1:4): 0.6; [α]_D_^20^ = +21 (*c* = 0.45, CHCl_3_); IR (film, cm^−1^) 3440, 2926, 1621, 1245, 1088; ^1^H NMR (400 MHz, CDCl_3_) δ 5.72–5.60 (m, 1H), 5.51–5.36 (m, 3H), 5.25 (dd, *J* = 15.4, 8.4 Hz, 1H), 4.97 (s, 1H), 4.88 (s, 1H), 4.68 (d, *J* = 6.8 Hz, 1H), 4.42 (d, *J* = 6.8 Hz, 1H), 4.21 (d, *J* = 6.1 Hz, 2H), 3.94 (dd, *J* = 9.2, 2.9 Hz, 1H), 3.91–3.84 (m, 2H), 3.74 (dd, *J* = 6.2, 3.4 Hz, 1H), 3.62 (d, *J* = 7.4 Hz, 1H), 3.44 (d, *J* = 3.3 Hz, 1H), 3.30 (s, 3H), 2.62–2.49 (m, 1H), 2.38–2.09 (m, 8H), 2.06 (dd, *J* = 5.3, 3.1 Hz, 1H), 2.04–2.00 (m, 1H), 1.62 (s, 3H), 1.45 (s, 3H), 1.35 (s, 3H), 1.25 (s, 3H), 1.09 (d, *J* = 6.2 Hz, 3H), 0.89 (s, 12H), 0.88 (s, 15H), 0.82 (d, *J* = 6.8 Hz, 3H), 0.13–−0.03 (m, 18H). To a stirred solution of the above TBS ether (114 mg, 0.12 mmol) in methanol (4 mL), we added ammonium fluoride (124 mg, 3.37 mmol) at rt and the reaction mixture was stirred at that temperature for 8 h. Et_2_O (30 mL) was added to precipitate the ammonium fluoride, which was removed by suction filtration. The crude product was purified by flash chromatography (EtOAc/hexane 1:4) to give the alcohol **39** as a colorless oil (90 mg, 90% yield). R_f_ value (EtOAc/hexane 2:1): 0.35; [α]_D_^20^ = +24 (*c =* 0.3, CHCl_3_); IR (film, cm^−1^) 3445, 2966, 2928, 1652, 1376, 1251, 1071; ^1^H NMR (400 MHz, CDCl_3_) δ 5.80–5.57 (m, 1H), 5.49 (t, *J* = 7.0 Hz, 1H), 5.45 (d, *J* = 15.6 Hz, 1H), 5.23 (dd, *J* = 15.5, 8.4 Hz, 1H), 4.92 (s, 1H), 4.84 (s, 1H), 4.68 (d, *J* = 6.8 Hz, 1H), 4.42 (d, *J* = 6.8 Hz, 1H), 4.16 (d, *J* = 6.7 Hz, 2H), 3.91–3.86 (m, 1H), 3.85 (dd, *J* = 8.9, 3.2 Hz, 1H), 3.77 (dd, *J* = 6.3, 3.2 Hz, 1H), 3.52 (d, *J* = 9.4 Hz, 1H), 3.30 (s, 3H), 2.35–2.11 (m, 6H), 2.08 (dd, *J* = 15.0, 3.1 Hz, 1H), 1.96–1.82 (m, 1H), 1.74 (d, *J* = 8.0 Hz, 1H), 1.68 (s, 3H), 1.59–1.48 (m, 1H), 1.44 (s, 3H), 1.35 (s, 3H), 1.17 (s, 3H), 1.09 (d, *J* = 6.3 Hz, 3H), 0.96 (d, *J* = 7.1 Hz, 3H), 0.89 (s, 9H), 0.87 (s, 9H), 0.84 (d, *J* = 7.0 Hz, 3H), 0.09–0.00 (m, 12H). ^13^C NMR (100 MHz, CDCl_3_) δ 143.35, 140.99, 133.85, 131.41, 130.87, 129.89, 124.82, 114.29, 107.06, 93.20, 81.99, 81.24, 79.35, 77.12, 73.18, 72.90, 70.61, 59.19, 55.36, 47.04, 43.54, 39.49, 39.41, 38.88, 37.16, 36.16, 35.43, 28.40, 26.51, 25.79, 25.71, 20.44, 17.97, 17.65, 15.82, 13.92, 13.49, −4.57, −4.66, −4.77, −5.07. MS (ESI, *m*/*z*) [M + Na]^+^ 845.5.

**Macrolactone (40)**: To a stirred solution of allyl alcohol **39** (85 mg, 0.104 mmol) in DCM (10 mL), we added activated MnO_2_ (36 mg, 90%, 0.416 mmol) at rt, and the reaction was stirred at rt for 3 h. Another portion of MnO_2_ (36 mg, 90%, 0.416 mmol) was added and was stirred for two more hours. Suction filtration gave a crude aldehyde (83 mg) that was used for the next step without further purification. R_f_ value (EtOAc/hexane 1:2): 0.7. To a stirred solution of thus obtained aldehyde (83 mg, 0.1 mmol) and 2-methyl-2-butene (2 mL) in *tert*-butanol (8 mL), we added a solution of NaH_2_PO_4_^.^H_2_O (200 mg) and NaClO_2_ (200 mg), dropwise, in H_2_O (2 mL) at 0 °C. In addition, the reaction mixture was allowed to warm up to rt and stirred for 20 min. The reaction was poured into water (5 mL) and extracted with ethyl acetate (3 × 20 mL). The combined organic extracts were washed with water and brine, dried over anhyd Na_2_SO_4_ and evaporated in vacuo. The crude product was purified by flash chromatography (MeOH/chloroform 3:100) to give the seco-acid as a colorless oil (77 mg, 88% yield for two steps). R_f_ value (EtOAc/hexane 1:2): 0.6.

To the solution of thus obtained seco-acid in THF (5 mL), we added DIPEA (0.24 mL, 1.38 mmol) and 2,4,6-trichlorobenzoyl chloride (0.14 mL, 0.92 mmol) at rt. The reaction was stirred for 3 h at that temperature, before the THF solvent was removed by vacuo. The the residue toluene (10 mL) was added and the solution was transferred to a stirred solution of DMAP (280 mg, 2.3 mmol) in toluene (180 mL) at rt over 16 h, through a syringe pump. The resulting mixture was stirred at rt for 48 h and poured into sat NaHCO_3_ (20 mL). The organic layer was separated and the aq was extracted with diethyl ether (3 × 20 mL). The combined organic phase was washed with water and brine, dried over anhyd MgSO_4_ and concentrated in vacuo. Column chromatography (EtOAc/hexane 1:40) provided macrolactone **40** as a colorless oil (56 mg, 75%). R_f_ value (EtOAc/hexane 1:10): 0.5; [α]_D_^20^ = +16 (*c =* 0.3, CHCl_3_); IR (film, cm^−1^) 2930, 1715, 1166, 1029; ^1^H NMR (400 MHz, CDCl_3_) δ 5.70–5.56 (m, 3H), 5.37 (d, *J* = 15.7 Hz, 1H), 5.28 (dd, *J* = 15.6, 7.5 Hz, 1H), 4.99 (t, *J* = 6.1 Hz, 1H), 4.96 (s, 1H), 4.84 (s, 1H), 4.66 (d, *J* = 6.7 Hz, 1H), 4.53 (d, *J* = 6.7 Hz, 1H), 4.11 (dd, *J* = 7.5, 3.4 Hz, 1H), 3.96–3.88 (m, 1H), 3.79–3.71 (m, 1H), 3.63 (dt, *J* = 9.1, 5.9 Hz, 1H), 3.47 (dd, *J* = 14.8, 7.7 Hz, 1H), 3.38 (s, 3H), 2.52 (dd, *J* = 7.0, 3.6 Hz, 1H), 2.36–2.23 (m, 1H), 2.19 (s, 3H), 2.18–2.11 (m, 2H), 2.12–2.05 (m, 2H), 1.93–1.77 (m, 1H), 1.68–1.58 (m, 1H), 1.45 (s, 3H), 1.37 (s, 3H), 1.22 (s, 3H), 1.18 (d, *J* = 7.0 Hz, 3H), 1.03 (d, *J* = 6.1 Hz, 3H), 0.88 (s, 9H), 0.87 (s, 9H), 0.84 (d, *J* = 6.6 Hz, 3H), 0.06–0 (m, 12H); ^13^C NMR (100 MHz, CDCl_3_) δ 166.21, 160.37, 141.98, 133.78, 130.36, 129.80, 129.66, 128.10, 117.71, 114.67, 107.00, 93.88, 81.80, 79.80, 78.93, 77.12, 73.77, 72.17, 71.36, 55.45, 48.73, 43.06, 39.77, 37.12, 36.09, 35.47, 34.96, 33.04, 28.34, 26.59, 25.77, 20.44, 19.81, 18.00, 17.43, 15.70, 13.68, 13.06, −4.34, −4.58, −4.70, −4.90. MS (ESI, m/z) [M + Na]^+^ 843.5.

**Diastereomer(2*E*, 19*S*)** (**29)**: To a stirred solution of TBS ether **40** (56 mg, 0.068 mmol) in THF (4 mL), we added pyridine (1 mL) followed by HF^.^pyridine complex (70%, 0.5 mL) at 0 °C, and the reaction was warmed up to rt and stirred for 16 h. The reaction mixture was cooled to 0 °C again and sat NaHCO_3_ was added until the bubbles disappeared. The organic layer was separated and the aq layer was extracted with diethyl ether (3 × 20 mL). The combined organic phase was washed with sat NaHCO_3_, water and brine, dried over anhyd MgSO_4_ and concentrated in vacuo. Column chromatography (EtOAc/hexane 1:2) provided diol as a colorless oil (31 mg, 76%). R_f_ value (EtOAc/hexane 2:1): 0.5. A solution of the above acetonide (31 mg, 0.052 mmol) in HOAc (2.4 mL) and water (0.6 mL) was heated at 55 °C for 3 h before the solvents were removed in vacuo. Column chromatography (MeOH/chloroform 1:20) provided the vicinal alcohol as a colorless oil (18 mg, 64%). R_f_ value (EtOAc/hexane 4:1): 0.2. [α]_D_^20^ = −9 (*c* = 0.14, CHCl_3_); IR (film, cm^−1^) 3442, 2962, 1715, 1639, 1431, 1016; ^1^H NMR (400 MHz, CDCl_3_) δ 5.76–5.65 (m, 1H), 5.60 (s, 1H), 5.54 (t, *J* = 7.8 Hz, 1H), 5.51 (d, *J* = 7.6 Hz, 1H), 5.38 (dd, *J* = 15.6, 7.3 Hz, 1H), 5.03 (s, 1H), 5.05-5 (m, 1H), 4.97 (s, 1H), 4.68 (d, *J* = 6.7 Hz, 1H), 4.57 (d, *J* = 6.8 Hz, 1H), 4.12 (dd, *J* = 7.3, 3.5 Hz, 1H), 3.74 (dd, *J* = 6.3, 3.7 Hz, 1H), 3.63 (d, *J* = 6.2 Hz, 1H), 3.58 (d, *J* = 10.3 Hz, 1H), 3.39 (s, 3H), 2.56 (dd, *J* = 6.9, 3.5 Hz, 2H), 2.36 (d, *J* = 14.5 Hz, 2H), 2.28 (d, *J* = 13.0 Hz, 1H), 2.17 (s, 3H), 2.13–1.94 (m, 4H), 1.8–1.65 (m, 1H), 1.6–1.40 (m, 2H), 1.47 (brs, 1H), 1.16 (d, *J* = 7.0 Hz, 3H), 1.13 (d, *J* = 6.4 Hz, 3H), 0.98 (d, *J* = 6.9 Hz, 3H), 0.89 (d, *J* = 6.7 Hz, 3H); ^13^C NMR (100 MHz, CDCl_3_) δ 160.79, 143.10, 135.04, 130.43, 129.83, 129.61, 117.55, 115.48, 94.16, 78.74, 77.12, 74.58, 73.59, 70.74, 68.04, 55.46, 48.29, 42.94, 39.72, 37.99, 36.48, 35.89, 35.82, 32.90, 19.87, 19.29, 18.46, 15.34, 14.09, 13.2.

To a stirred solution of MOM ether (11 mg, 0.02 mmol) in DCM (2 mL), we added B-bromocatechol borane (0.5 mL, 0.2 M soln in DCM, 0.1 mmol) at −78 °C, and the mixture was stirred at that temperature for 1h, before it was quenched with sat NaHCO_3_ (5 mL). The organic layer was separated and the aq layer was extracted with ethyl acetate. The combined organic phase was washed with water and brine, dried over anhyd Na_2_SO_4_ and concentrated in vacuo. Column chromatography (MeOH/chloroform 1:20) provided the corresponding alcohol as a colorless oil (6.7 mg, 66%). R_f_ value (EtOAc/hexane/MeOH 8:2:1): 0.3.

To a stirred solution of thus obtained alcohol (3 mg, 0.0059 mmol) and DMAP (9 mg, 0.074 mmol) in DCM (2 mL), we added TESCl (0.01 mL, 0.059 mmol) at 0 °C, and the reaction was stirred at 0 °C for 30 min before sat NaHCO_3_ (5 mL) was added. The organic layer was separated and the aq layer was extracted with diethyl ether (3 × 10 mL). The combined organic phase was washed with water and brine, dried over anhyd MgSO_4_ and concentrated in vacuo. Column chromatography (EtOAc/hexane 1:20) provided *tris*-TES ether as a colorless oil (4.4 mg, 88%). R_f_ value (EtOAc/hexane 1:4): 0.5.

To a stirred solution of thus obtained diol (2.4 mg, 2.8 μmol) in DCM (1 mL), we added DMSO (40 μL, 0.141 mmol), DIPEA (49 μL, 0.282 mmol) and SO_3_^.^Py (22.4 mg, 0.141 mmol) at 0 °C. The mixture was stirred at rt for 30 min. The solvent was removed by high vacuum and THF (1 mL) was added, followed by a HF·Py solution (0.3 mL containing 1 mL 70% HF·Py: 1.1 mL pyridine: 2.4 mL THF) at rt. The reaction mixture was stirred for 1h, before it was quenched with sat NaHCO_3_ and extracted with diethyl ether (3 × 10 mL). The combined organic layer was washed with water and brine, dried over MgSO_4_ and concentrated under reduced pressure. The resulting residue was purified by flash chromatography (MeOH/chloroform 1:25) to give diastereomer **29** (1 mg, 71%) as a colorless oil. R_f_ value (EtOAc/hexane 2:1): 0.3. [α]_D_^20^ = +35.7 (*c =* 0.042, CH_2_Cl_2_); IR (film, cm^−1^) 3465, 2980, 2360, 1695, 1452, 1369, 1222, 1004; ^1^H NMR (500 MHz, CDCl_3_) δ 5.77–5.70 (m, 1H), 5.67 (d, *J* = 18.6 Hz, 2H), 5.58–5.51 (m, 1H), 5.47 (dd, *J* = 16.0, 7.4 Hz, 1H), 4.86 (s, 1H), 4.84 (s, 1H), 4.83–4.79 (m, 1H), 4.21 (dd, *J* = 8.2, 3.8 Hz, 1H), 4.07–3.95 (m, 1H), 3.80 (dd, *J* = 6.3, 3.4 Hz, 1H), 3.35 (s, 1H), 3.06 (brs, 1H), 2.67 (dd, *J* = 6.8, 3.9 Hz, 1H), 2.50 (d, *J* = 14.4 Hz, 1H), 2.33 (m, 1H), 2.20 (m, 2H), 2.18 (d, *J* = 0.8 Hz, 3H), 2.15–2.08 (m, 2H), 2.07–1.96 (m, 2H), 1.94–1.81 (m, 1H), 1.78–1.69 (m, 1H), 1.51–1.45 (m, 1H), 1.45–1.37 (m, 1H), 1.30 (s, 3H), 1.15 (d, *J* = 6.4 Hz, 3H), 1.13 (d, *J* = 7.1 Hz, 3H), 1.02 (d, *J* = 6.9 Hz, 3H), 0.89 (d, *J* = 6.8 Hz, 3H); ^13^C NMR (126 MHz, CDCl_3_) δ 159.2, 141.4, 134.1, 130.5, 130.3, 129.6, 118.2, 110.9, 99.2, 73.1, 69.7, 48.6, 38.3, 37.85, 37.1, 36.5, 35.7, 35.3, 33.6, 24.6, 23.2, 19.5, 18.7, 14.8, 14.3, 10.5. MS (ESI, *m*/*z*) [M + Na]^+^ 529.31.

**Diastereomer (2*E*, 9*R***) **(30):** [α]_D_^20^ = +18.8 (*c =* 0.07, CH_2_Cl_2_); IR (film, cm^−1^) 3380, 2924, 1690, 1684,1541, 1508, 1458, 1212; ^1^H NMR (500 MHz, CDCl_3_) δ 9.42 (s, 1H), 6.22 (d, *J* = 11.0 Hz, 1H), 5.73–5.59 (m, 3H), 5.52 (dd, *J* = 15.6, 8.1 Hz, 1H), 5.02–4.91 (m, 2H), 4.89 (s, 1H), 4.42 (dd, *J* = 8.0, 3.6 Hz, 1H), 3.85 (dd, *J* = 6.3, 3.4 Hz, 1H), 3.56–3.50 (m, 1H), 3.46–3.38 (m, 1H), 2.63 (dd, *J* = 6.8, 4.0 Hz, 1H), 2.31–2.10 (m, 6H), 2.19 (d, *J* = 0.9 Hz, 3H), 2.04 (dd, *J* = 13.8, 9.3 Hz, 1H), 1.94 (dd, *J* = 14.2, 9.1 Hz, 1H), 1.80 (s, 3H), 1.54–1.45 (m, 1H), 1.38–1.28 (m, 1H), 1.16 (d, *J* = 6.9 Hz, 3H), 1.11 (d, *J* = 6.4 Hz, 3H), 0.90–0.80 (m, 6H); ^13^C NMR (126 MHz, CDCl_3_) δ 191.2, 167.0, 161.0, 153.3, 143.0, 135.5, 130.7, 130.5, 115.6, 115.3, 75.8, 73.7, 69.5, 48.5, 45.6, 42.7, 40.2, 35.9, 35.9, 35.7, 35.1, 32.1, 20.5, 19.9, 16.7, 16.5, 14.9. MS (ESI, *m*/*z*) [M + Na]^+^ 529.33.

**Diastereomer(4*S*, 5*R*) (31):** [α]_D_^20^ = −15 (*c =* 0.03, CH_2_Cl_2_); IR (film, cm^−1^) 3470, 2987, 2975, 2360, 1684, 1255, 1005; ^1^H NMR (500 MHz, CDCl_3_) δ 5.87 (s, 1H), 5.86–5.74 (m, 3H), 5.57 (dd, *J* = 15.7, 7.2 Hz, 1H), 5.12 (dd, *J* = 11.5, 5.8 Hz, 1H), 4.88 (s, 1H), 4.85 (s, 1H), 4.16–4.07 (m, 1H), 3.94–3.85 (m, 1H), 3.80 (dt, *J* = 10, 6.2 Hz, 1H), 3.42 (dq, *J* = 13.9, 6.9 Hz, 1H), 2.76 (brs, 1H), 2.45–2.32 (m, 3H), 2.33–2.22 (m, 3H), 2.18 (d, *J* = 11.2 Hz, 1H), 2.08–1.93 (m, 4H), 1.89 (s, 3H), 1.79–1.7 (m, 1H), 1.5–1.41 (m, 1H), 1.13 (d, *J* = 6.3 Hz, 3H), 1.06 (d, *J* = 6.9 Hz, 3H), 0.95 (d, *J* = 6.6 Hz, 3H), 0.91 (d, *J* = 6.8 Hz, 3H); ^13^C NMR (126 MHz, CDCl_3_) δ 167.3, 155.5, 141.5, 134.66, 132.0, 130.0, 128.4, 120.4, 111.1, 99.4, 75.1, 70.3, 69.7, 53.3, 42.6, 39.6, 36.8, 36.8, 35.8, 34.8, 34.1, 32.8, 21.9, 19.7, 19.0, 16.7, 15.8, 14.8. MS (ESI, *m*/*z*) [M + Na]^+^ 529.33.

## 4. Conclusions

In summary, we completed an efficient synthesis of the proposed structures of iriomoteolide-1a and iriomoteolide-1b. In an effort toward the assignment of correct structures, we rationally designed and synthesized three diastereomers of iriomoteolide-1a. The synthesis of the C1–C15 fragment features an ene reaction and Julia–Kocienski olefination. The trisubstituted *Z*-enoate or *E*-enoate of fragment C1–C6 was constructed from a carbocupration of the corresponding acetylene ester by utilizing a Gilman reagent. Other key reactions involved Seebach–Fráter asymmetric alkylation and enzyme kinetic resolution. Of particular interest, spectral data of synthetic iriomoteolide-1a and iriomoteolide-1b did not correlate with those reported for natural iriomoteolide-1a and -1b. Therefore, the structures of both natural iriomoteolide-1a and iriomoteolide-1b were assigned incorrectly. Our convergent synthetic routes to iriomoteolides provided efficient access to a variety of diastereomers and structural derivatives. We synthesized three rationally designed diastereomers of iriomoteolide-1a for the assignment of structures and evaluation of bioactivity. While the NMR data of these diastereomeric compounds are closer to those reported for natural iriomoteolide-1a, they did not completely match the natural product NMRs. We evaluated our synthetic iriomoteolide-1a, iriomoteolide -1b, and three diastereomers. However, none of these iriomoteolide derivatives showed any appreciable cytotoxicity in cancer cell lines. Further work towards the assignment of iriomoteolide structures is in progress.

## Data Availability

The data presented in this study are available in the manuscript and in the Supplementary Materials.

## Supplementary Materials

The following supporting information can be downloaded at: https://www.mdpi.com/article/10.3390/md20100587/s1. Table S1: 1H and 13C NMR (CDCl3) data for natural and synthetic iriomoteolide -1a (1). Table S2: 1H and 13C NMR (CDCl3) data for natural and synthetic iriomoteolide -1b (2). Figures S1–S25: Characterization data, 1H-NMR, and 13C NMR spectra.
