# Supplementary material for: Total Syntheses of the Proposed Structure of Iriomoteolide-1a, -1b and Synthesis of Three Derivatives for Structural Studies
Source: Mar Drugs. 2022 Sep 20;20(10):587. doi: 10.3390/md20100587 (PMC9605196; doi:10.3390/md20100587)
Supplement: Supplementary file 1 [file marinedrugs-20-00587-s001.zip › marinedrugs-1917426-supplementary.pdf]

Supporting Information

Total Syntheses of the Proposed Structure of Iriomoteolide-1a, -1b and Synthesis of Three derivatives for Structural Studies

Arun K. Ghosh,[1,*] and Hao Yuan[1]

[1]Department of Chemistry and Department of Medicinal Chemistry, Purdue University, West Lafayette, IN 47907, USA.

*Correspondence: akghosh@purdue.edu

Table of Contents

General Methods..........1

Tables S1 and S2..........2-3

Figure S1–Figure S25: ^{1}H- and ^{13}C-NMR Spectra..........4-26

General Methods.

All reactions were carried out under an argon atmosphere in either flame or oven-dried (120 °C) glassware. All reagents and chemicals were purchased from commercial suppliers and used without further purification unless otherwise noted. Anhydrous solvents were obtained as follows: Dichloromethane from calcium hydride, methanol and ethanol from activated magnesium under argon. All purification procedures were carried out with reagent grade solvents (purchased form VWR) in air. TLC analysis was conducted using glass-backed Thin-Layer Silica Gel Chromatography Plates (60 Å, 250 μm thickness, F-254 indicator). Column chromatography was performed using 230-400 mesh, 60 Å pore diameter silica gel. ^{1}H, ^{13}C NMR spectra were recorded at room temperature on a Bruker ARX-400, DRX-500, and Bruker AV800. Chemical shifts (δ values) are reported in parts per million, and are referenced to the deuterated residual solvent peak. NMR data is reported as: δ value (chemical shift, J-value (Hz), integration, where s = singlet, d = doublet, t = triplet, q = quartet, brs = broad singlet). LRMS and HRMS spectra were recorded at the Purdue University Department of Chemistry Mass Spectrometry Center. HPLC analysis and purification was done an on Agilent 1260 series instrument using a YMC Pack ODS-A column of 4.6 mm ID for analysis and either 10 mm ID or 20 mm ID for purification. The purity of all test compounds was determined by HPLC analysis to be ≥90% pure.

Table S1. ^{1}H and ^{13}C NMR ($CDCl_3$) data for natural and synthetic iriomoteolide -1a (1)

Position	^{1}H Natural	$J_{natural}$	^{1}H Synthetic	$J_{synthetic}$	^{13}C Natural	^{13}C synthetic
1					167.4	166.3
2	5.72	brs	5.78	s	115.8	118.8
3					162.0	160.0
4	2.46	dq, 2.9, 7.3	2.12-2.38	m	47.9	40.6
5	4.28	m	4.07	m	72.3	74.9
6	5.57	dd, 4.1, 15.7	5.70	dd, 8.0, 15.0	132.0	132.7
7	5.68	m	5.80-5.90	m	126.8	127.2
8a	2.18	m	2.12-2.38	m	39.5	37.8
8b	2.00		2.12-2.38	m		
9	3.81	brt, 11.5	3.98-4.03	m	71.8	70.3
10a	2.21	brd, 12.7	2.12-2.38	m	40.7	39.7
10b	1.90	brt, 12.3	2.12-2.38	m		
11					141.7	141.7
12a	2.40	d, 13.6	2.12-2.38	m	36.9	37.3
12b	2.26	brd, 13.6	2.12-2.38	m		
13	3.52	brd, 1.69	3.28	brs, -OH	99.7	99.1
14					77.2	77.2
15	5.68	brd, 15.5	5.80–5.90	m	134.69	134.8
16	5.76	ddd, 3.1, 10.8, 15.5	5.80–5.90	m	128.8	129.6
17a	2.15	m	2.12-2.38	m	38.2	34.2
17b	1.96	dt, 14.1, 11.6				
18	1.82	m	1.9-2.0	m	36.9	36.7
19	5.11	m	4.99-5.05	m	70.8	74.4
20a	1.80	ddd, 4.4, 8.7, 13.8	1.78-1.84	m	36.5	34.2
20b	1.15	ddd, 4.4, 8.8, 13.8	1.10-1.20	m		
21	1.40	m	1.30-1.40	m	36.5	35.5
22	3.58	quint, 6.3	3.83-3.89	m	72.2	69.3
23	1.11	d, 6.3	1.10	d, 6.4	19.8	20.0
24	2.12	s	1.96	brs	23.8	20.8
25	1.24	d, 7.3	1.12	d, 6.6	15.6	15.7
26a	4.82	brs	4.88	s	110.6	110.8
26b			4.86	s		
27	1.25	s	1.33	s	23.1	20.6
28	0.99	d, 6.8	1.01	d, 7.0	14.2	15.8
29	0.91	d, 6.7	0.89	d, 6.9	15.5	14.69

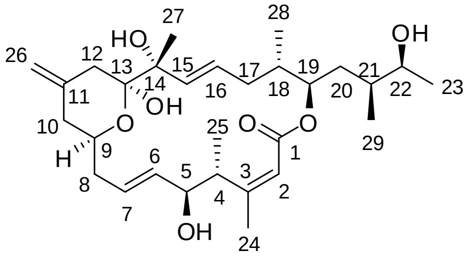

Iriomoteolide-1a (**1**)

Table S2. ^{1}H and ^{13}C NMR ($CDCl_3$) data for natural and synthetic iriomoteolide -1b (2)

Position	^{1}H Natural	$J_{natural}$	^{1}H Synthetic	$J_{synthetic}$	^{13}C Natural	^{13}C synthetic
1					166.7	167.2
2	5.63	brs	5.82	brs	116.2	118.6
3					160.8	159.3
4	2.62	m	2.15-2.35		48.7	48.3
5	4.46	m	4.15	m	72.8	75.5
6	5.56	m	5.70-5.75	m	132.4	132.9
7	5.56	m	5.70-5.75	m	129.6	126.5
8a	2.30	m	2.15-2.35	m	40.8	40.4
8b	2.08	m	2.15-2.35	m		
9	3.72	m	3.90	m	68.3	68.2
10a	2.28	m	2.15-2.35		48.6	40.9
10b	1.23	m	2.15-2.35			
11					157.5	160.1
12	6.18	s	6.32	s	121.8	120.5
13					200.8	200.7
14	4.33	brs, -OH	4.55	s, -OH	77.9	77.6
15	5.58	d, 15.6	5.54	d, 15.7	133.2	136.0
16	5.74	ddd, 4, 10, 15.6	5.80	m	130.4	129.8
17a	2.24	m	2.15-2.35	m	34.7	31.5
17b	1.75	m	2.15-2.35	m		
18	1.72	m	1.65	m	37.2	36.2
19	4.92	dt, 8.2, 3.3	4.98	dt, 7.5, 3.0	74.7	76.9
20a	1.76	m	1.92-1.96	m	32.4	30.9
20b	1.13	m	1.92-1.96	m		
21	1.45	m	1.35-1.42	m	36.5	35.6
22	3.61	m	3.80	m	72.2	69.4
23	1.13	d, 6.3	1.12	d, 6.8	19.7	15.7
24	2.20	s	1.99	brs	20.4	21.6
25	1.18	d, 7.3	1.20	d, 7.1	11.2	13.7
26a	2.17	s	2.27	s	20.0	20.1
27	1.44	s	1.33	s	25.2	22.6
28	0.90	d, 6.8	0.90	d, 7.0	14.6	14.0
29	0.87	d, 6.7	0.88	d, 6.4	14.8	15.0

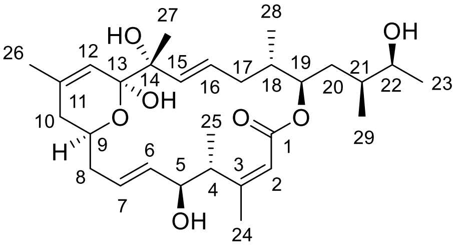

Iriomoteolide-1b (**2**)

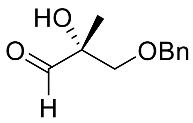

7

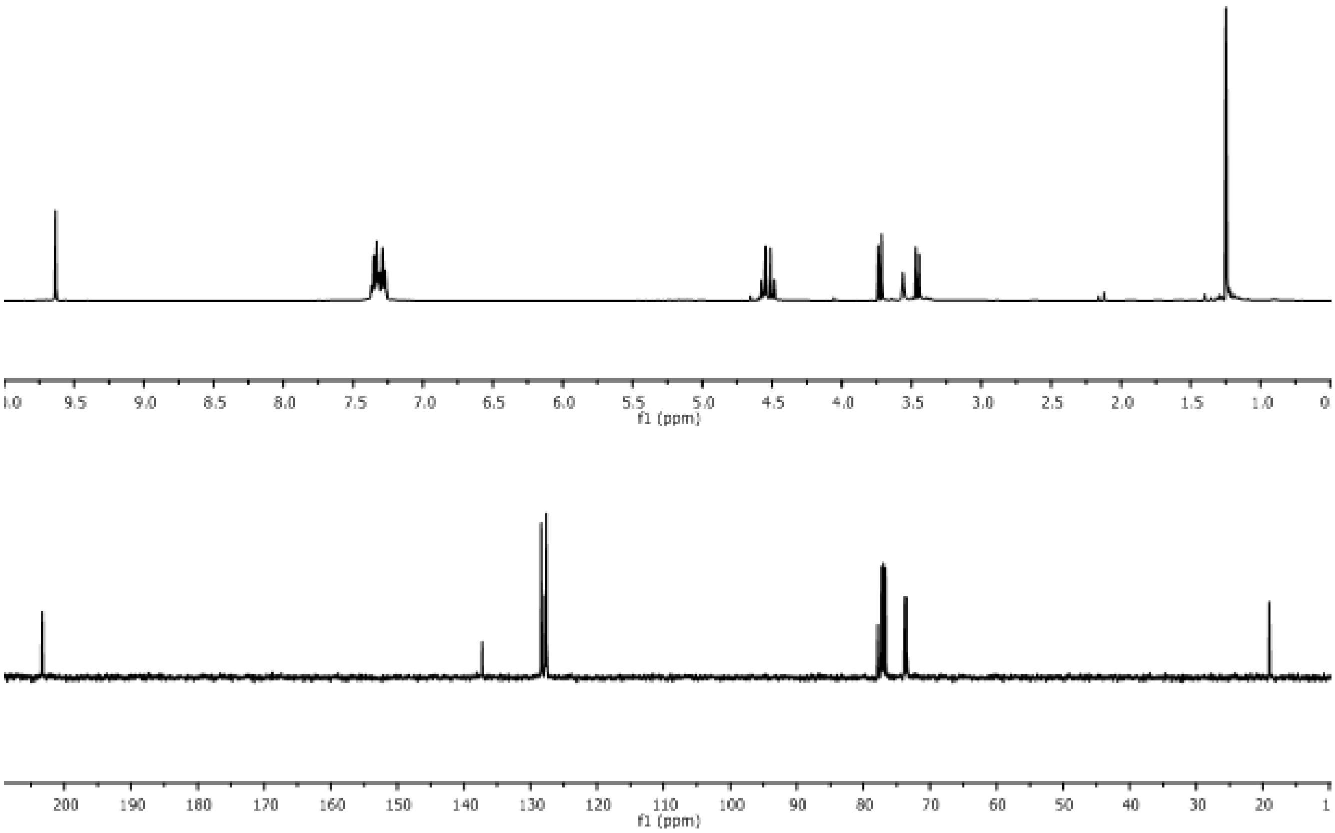

Figure S1 ^{1}H NMR (400 MHz, $CDCl_3$) and ^{13}C NMR (100 MHz, $CDCl_3$) of **7**

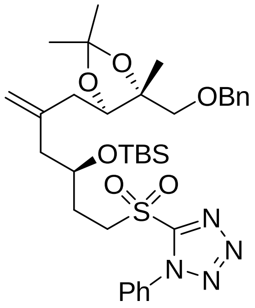

5

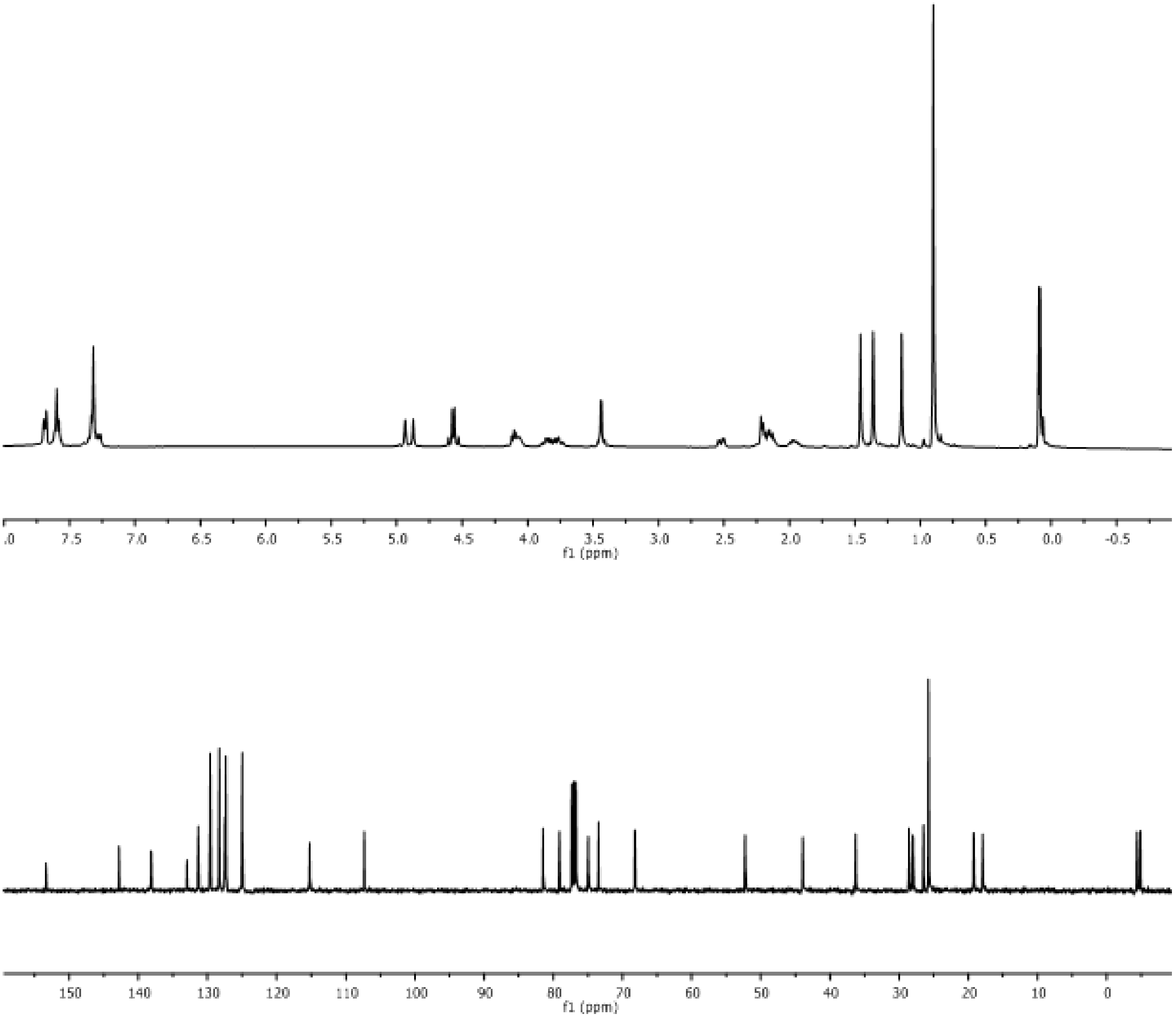

Figure S2 ¹H NMR (400 MHz, $CDCl_3$) and ¹³C NMR (100 MHz, $CDCl_3$) of **5**

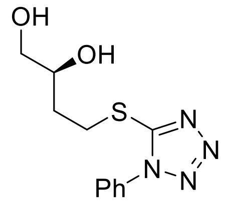

13

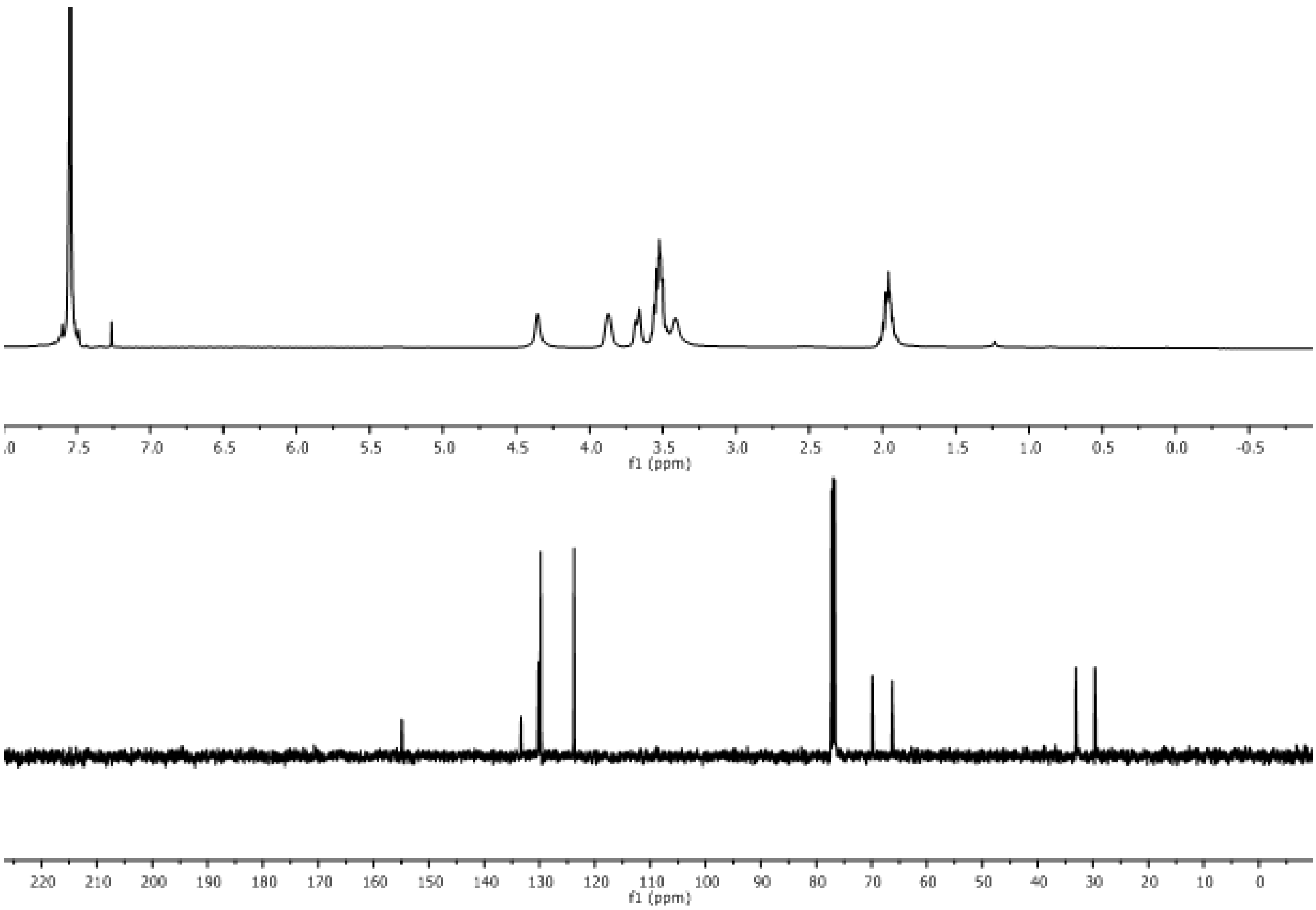

Figure S3 ^{1}H NMR (400 MHz, $CDCl_3$) and ^{13}C NMR (100 MHz, $CDCl_3$) of **13**

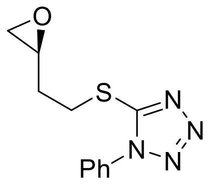

14

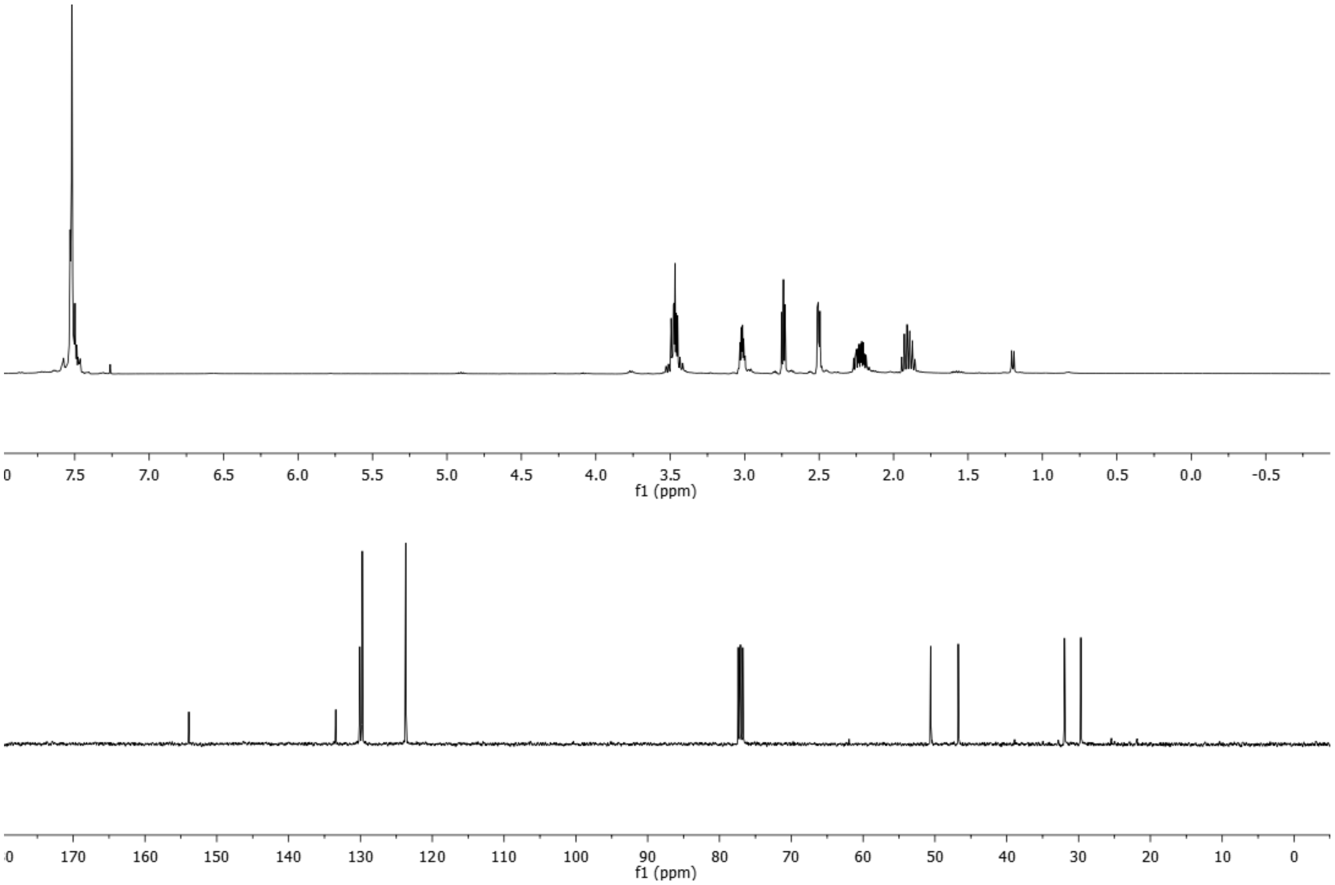

Figure S4 ^{1}H NMR (400 MHz, $CDCl_3$) and ^{13}C NMR (100 MHz, $CDCl_3$) of **14**

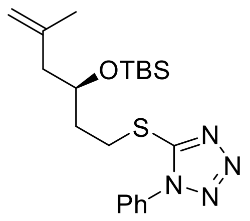

8

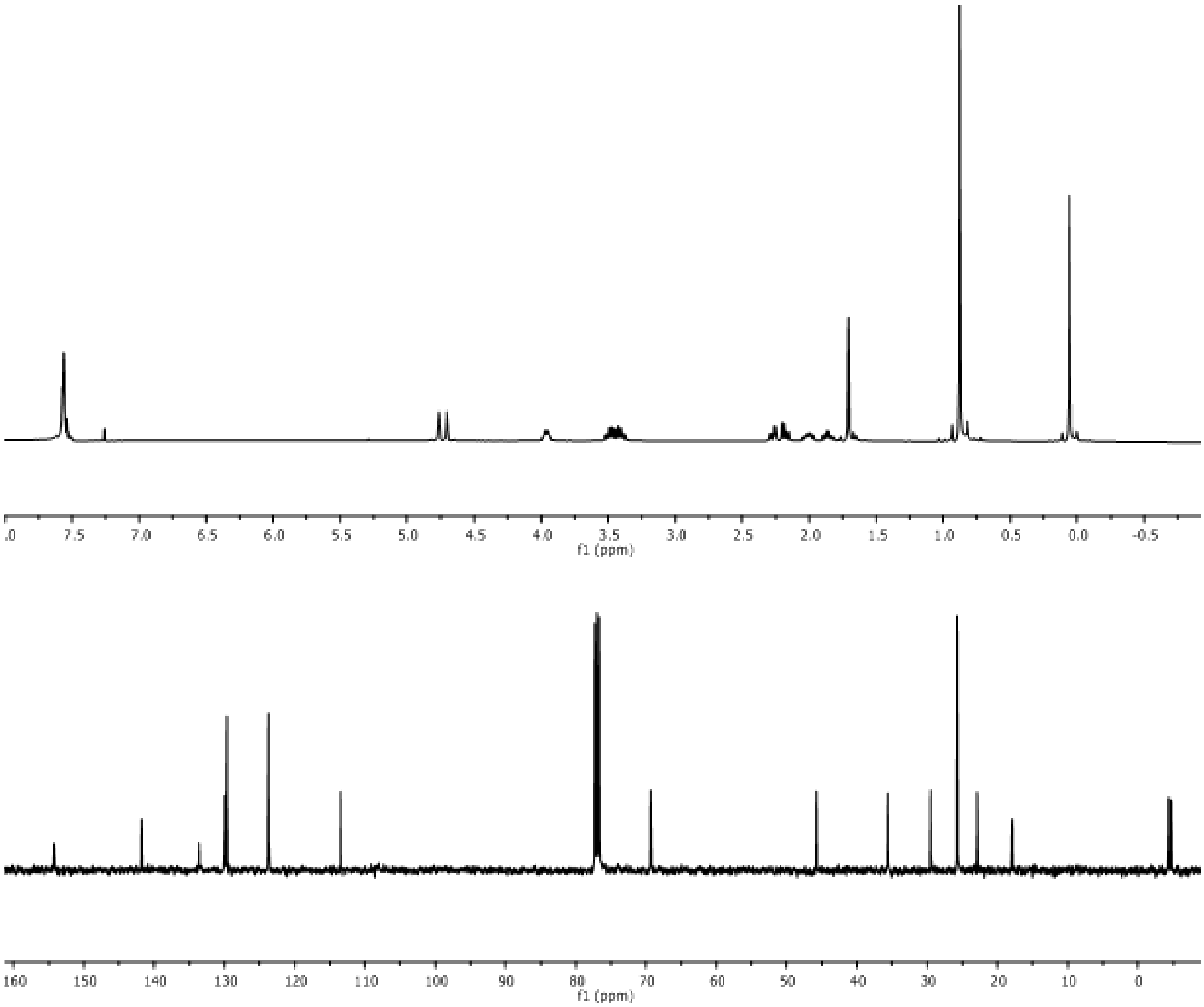

Figure S5 ^{1}H NMR (400 MHz, $CDCl_3$) and ^{13}C NMR (100 MHz, $CDCl_3$) of **8**

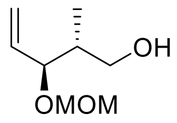

20

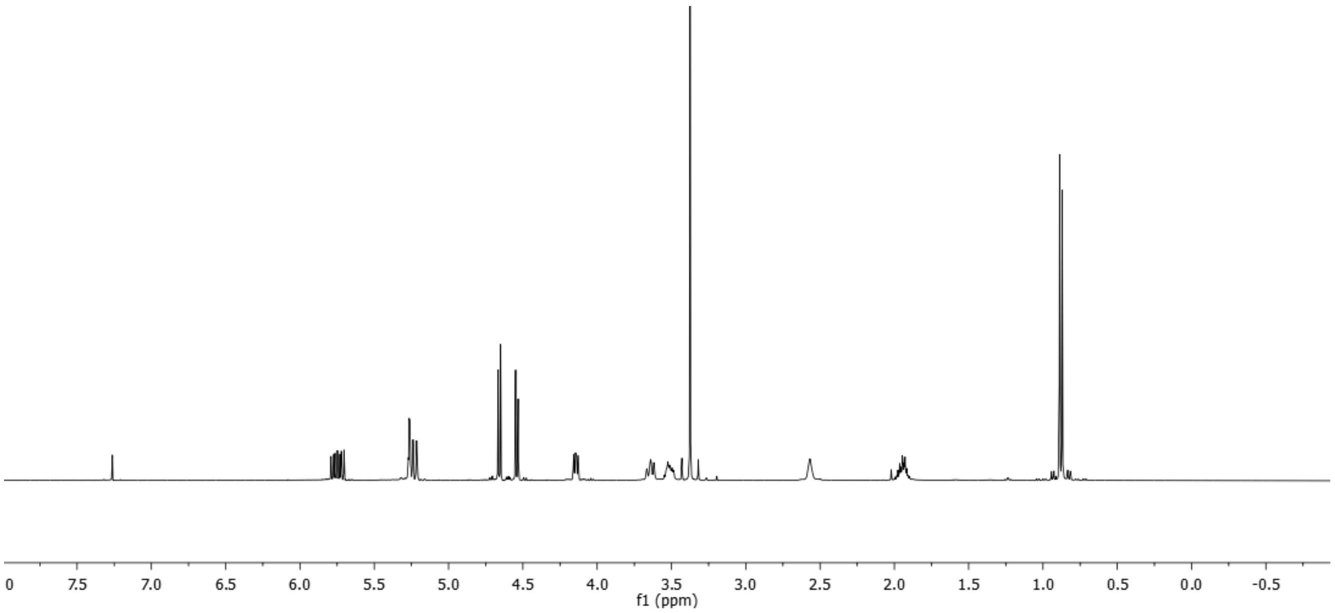

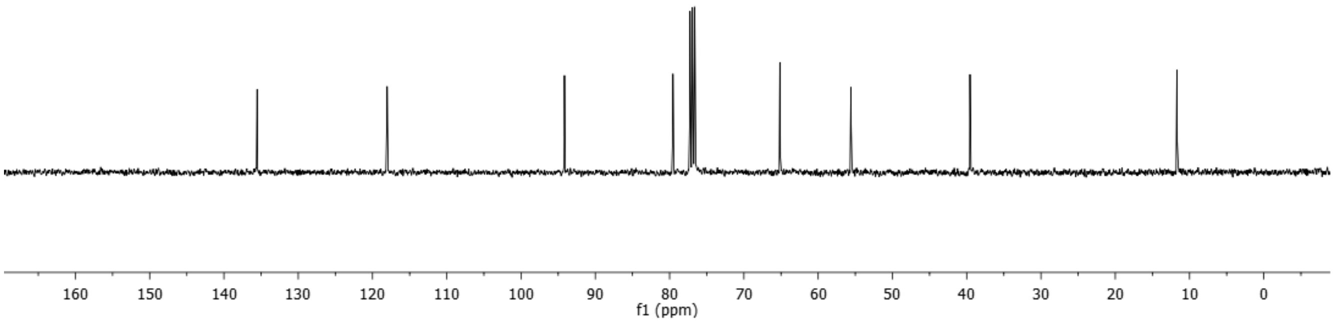

Figure S6 ^{1}H NMR (400 MHz, $CDCl_3$) and ^{13}C NMR (100 MHz, $CDCl_3$) of **20**

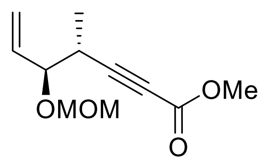

21

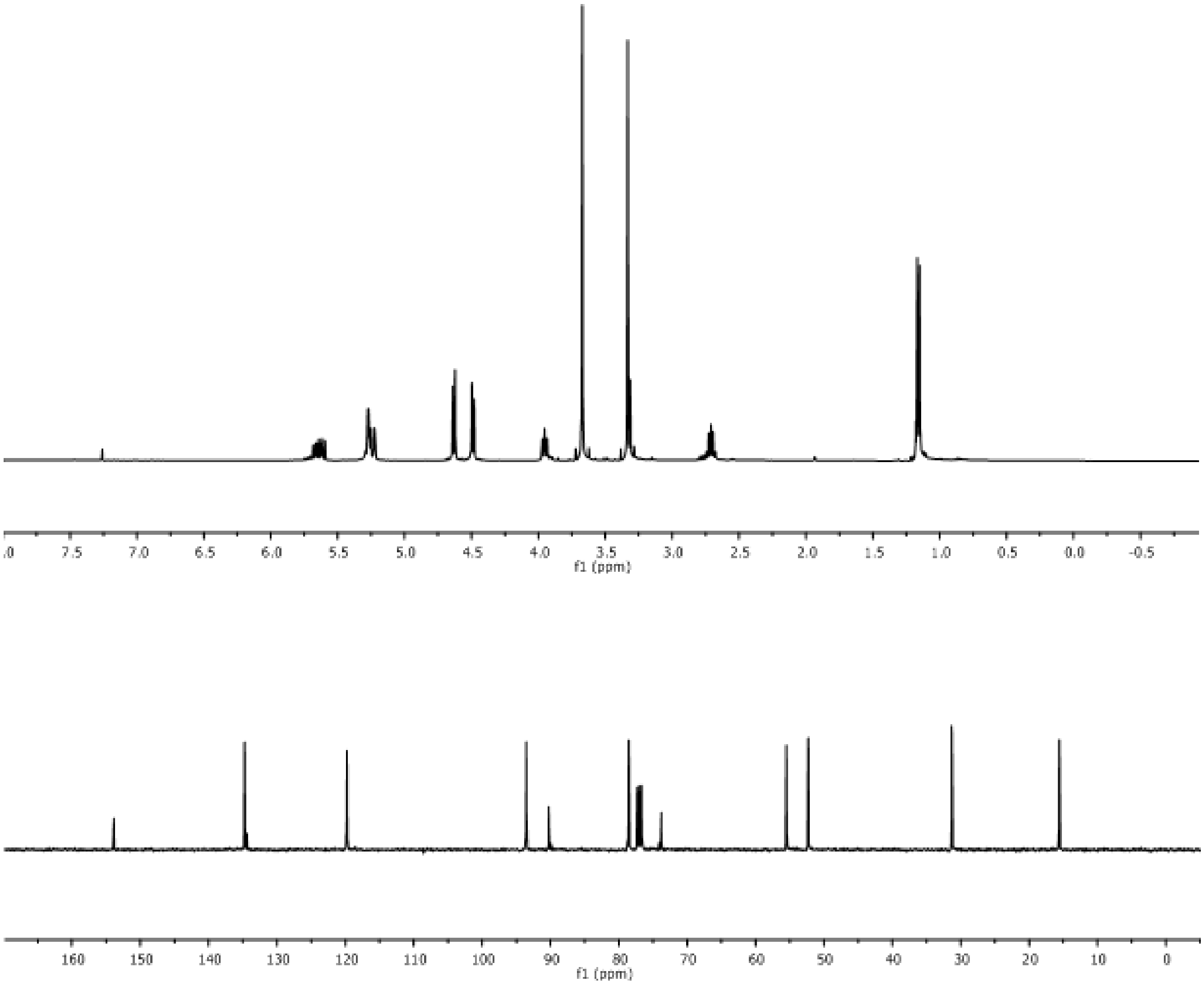

Figure S7 ^{1}H NMR (400 MHz, $CDCl_3$) and ^{13}C NMR (100 MHz, $CDCl_3$) of **21**

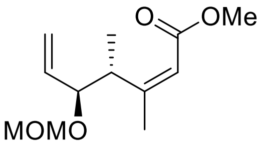

22

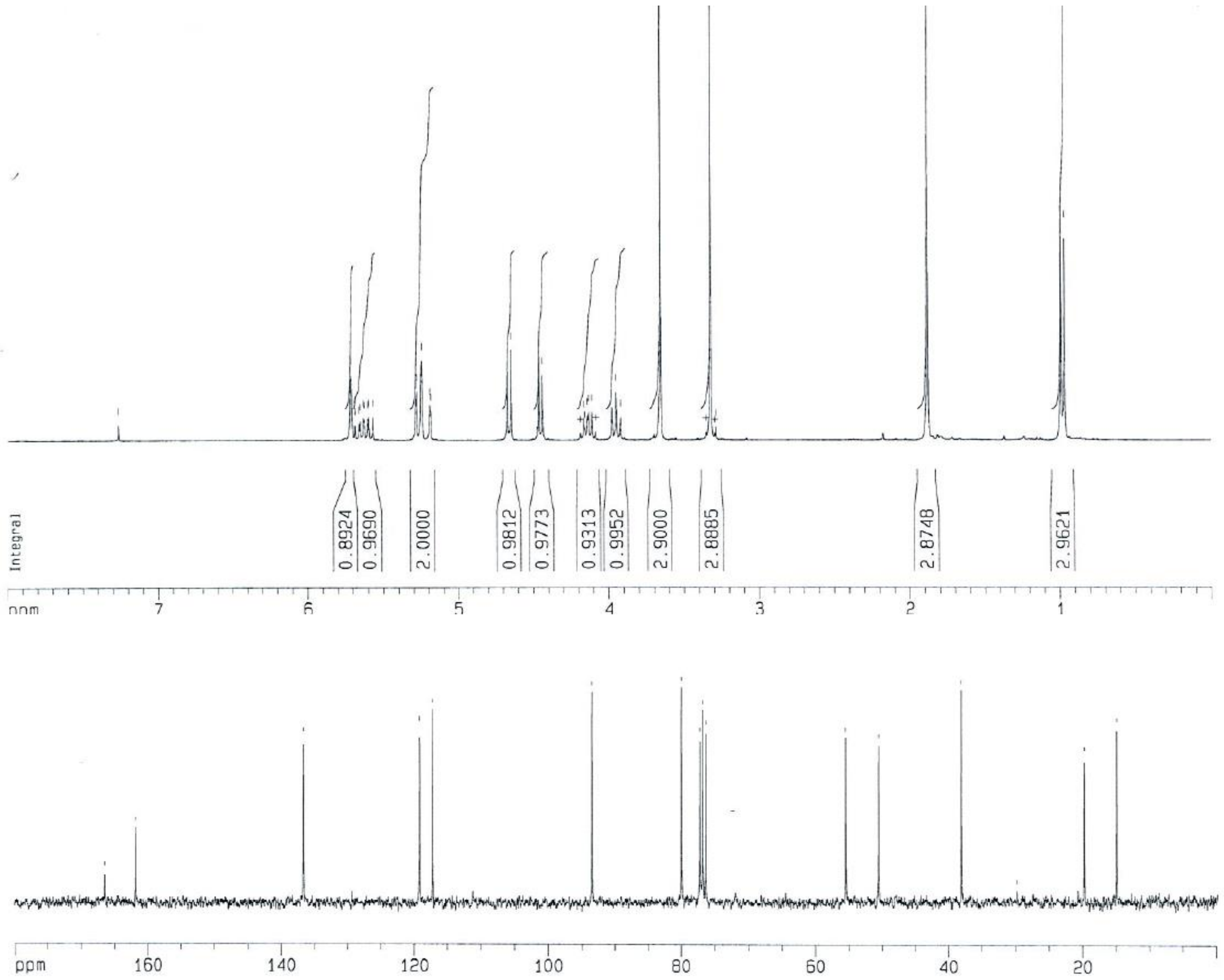

Figure S8 ^{1}H NMR (300 MHz, $CDCl_3$) and ^{13}C NMR (75 MHz, $CDCl_3$) of **22**

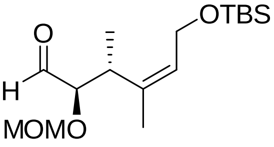

6

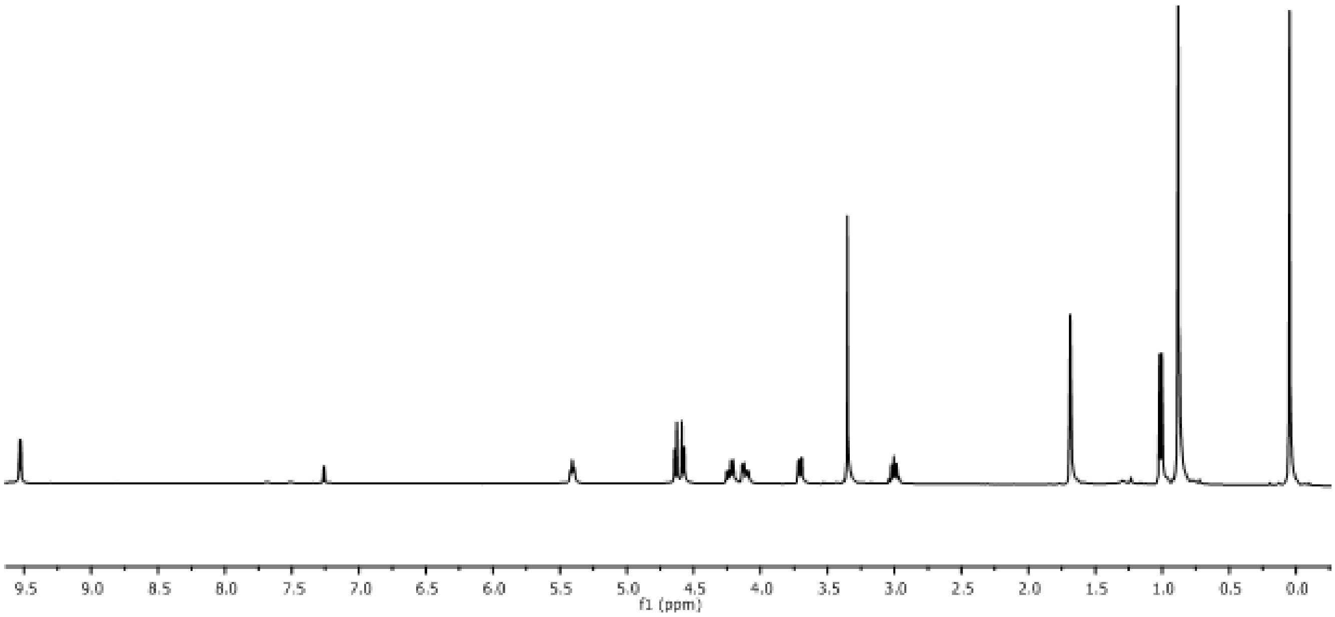

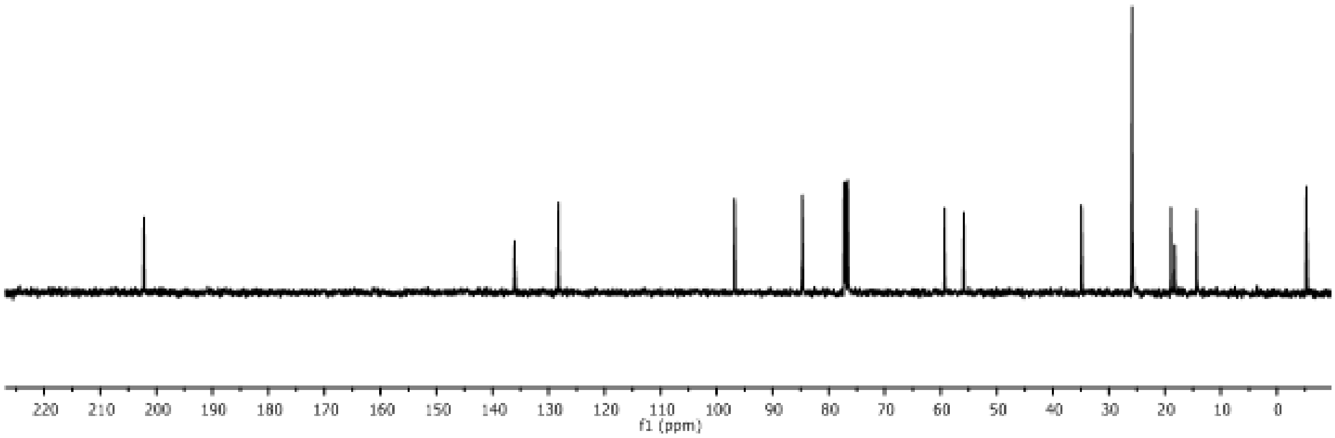

Figure S9 ^{1}H NMR (400 MHz, $CDCl_3$) and ^{13}C NMR (100 MHz, $CDCl_3$) of **6**

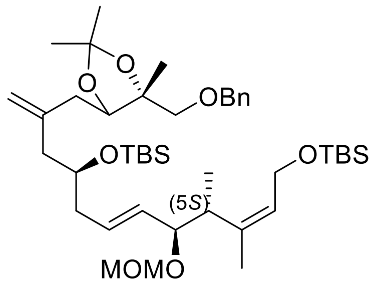

23

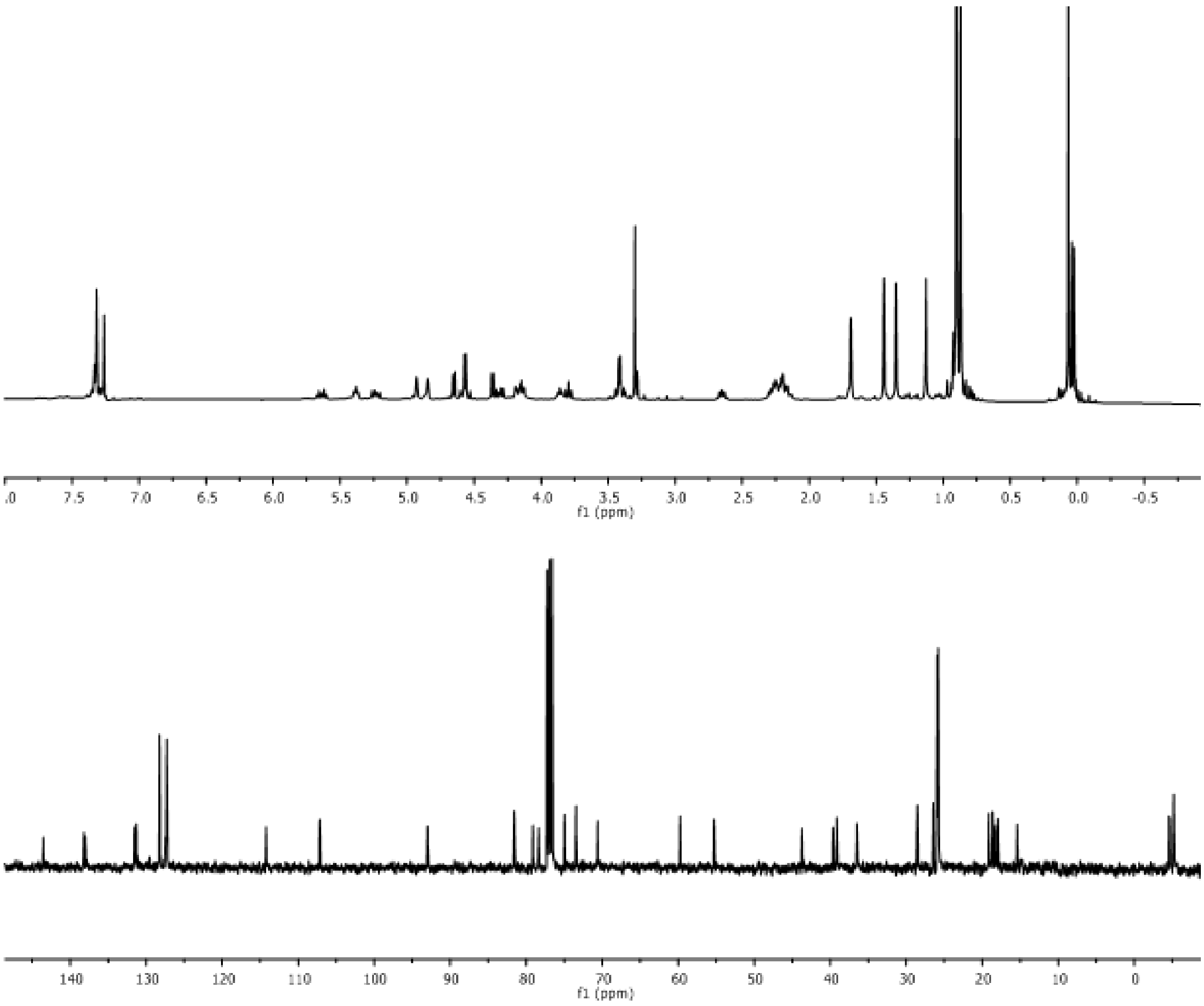

Figure S10 ¹H NMR (400 MHz, $CDCl_3$) and ¹³C NMR (100 MHz, $CDCl_3$) of **23**

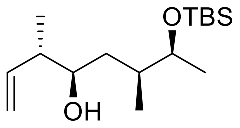

17

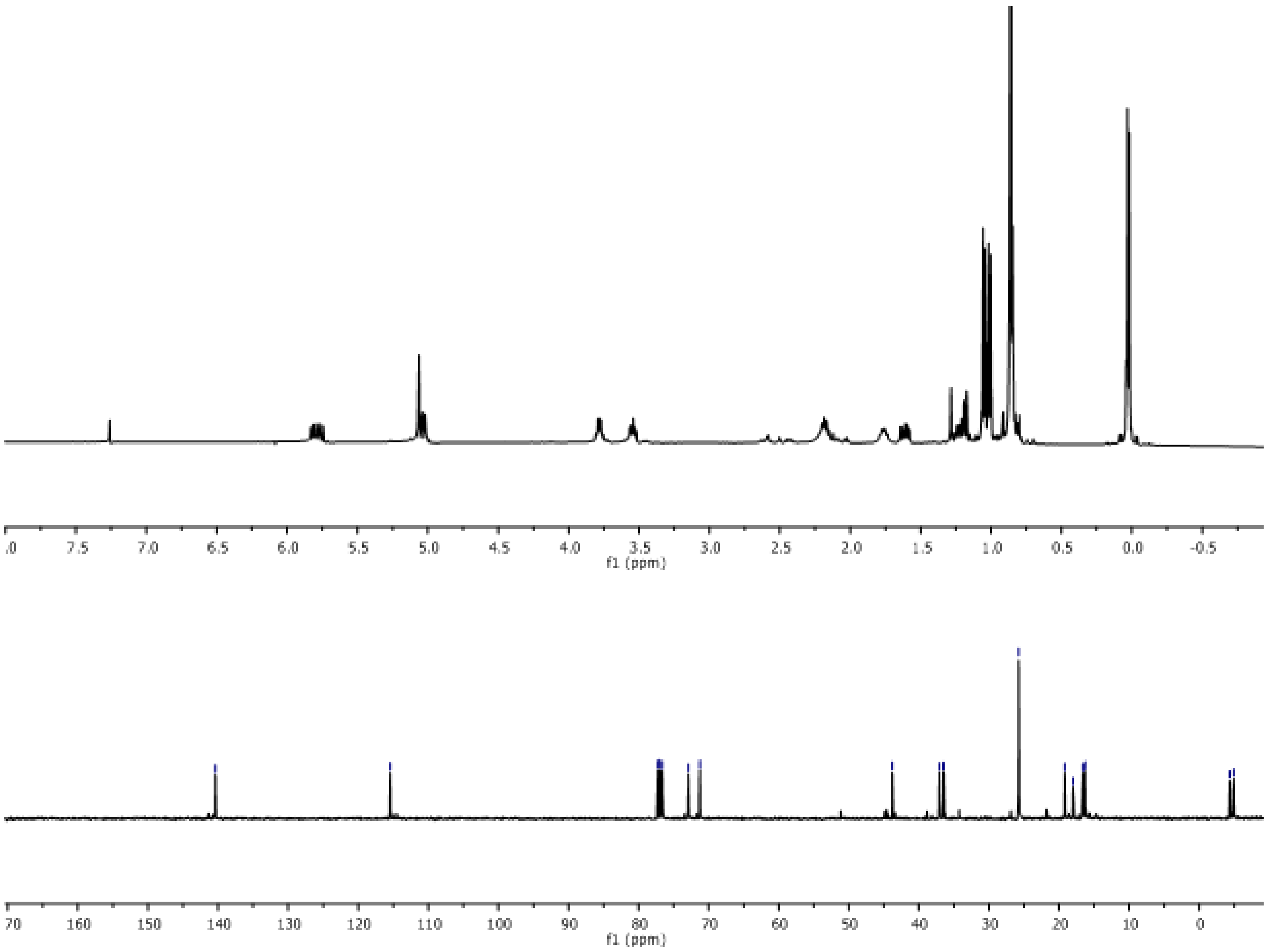

Figure S11 ^{1}H NMR (400 MHz, $CDCl_3$) and ^{13}C NMR (100 MHz, $CDCl_3$) of **17**

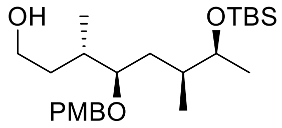

18

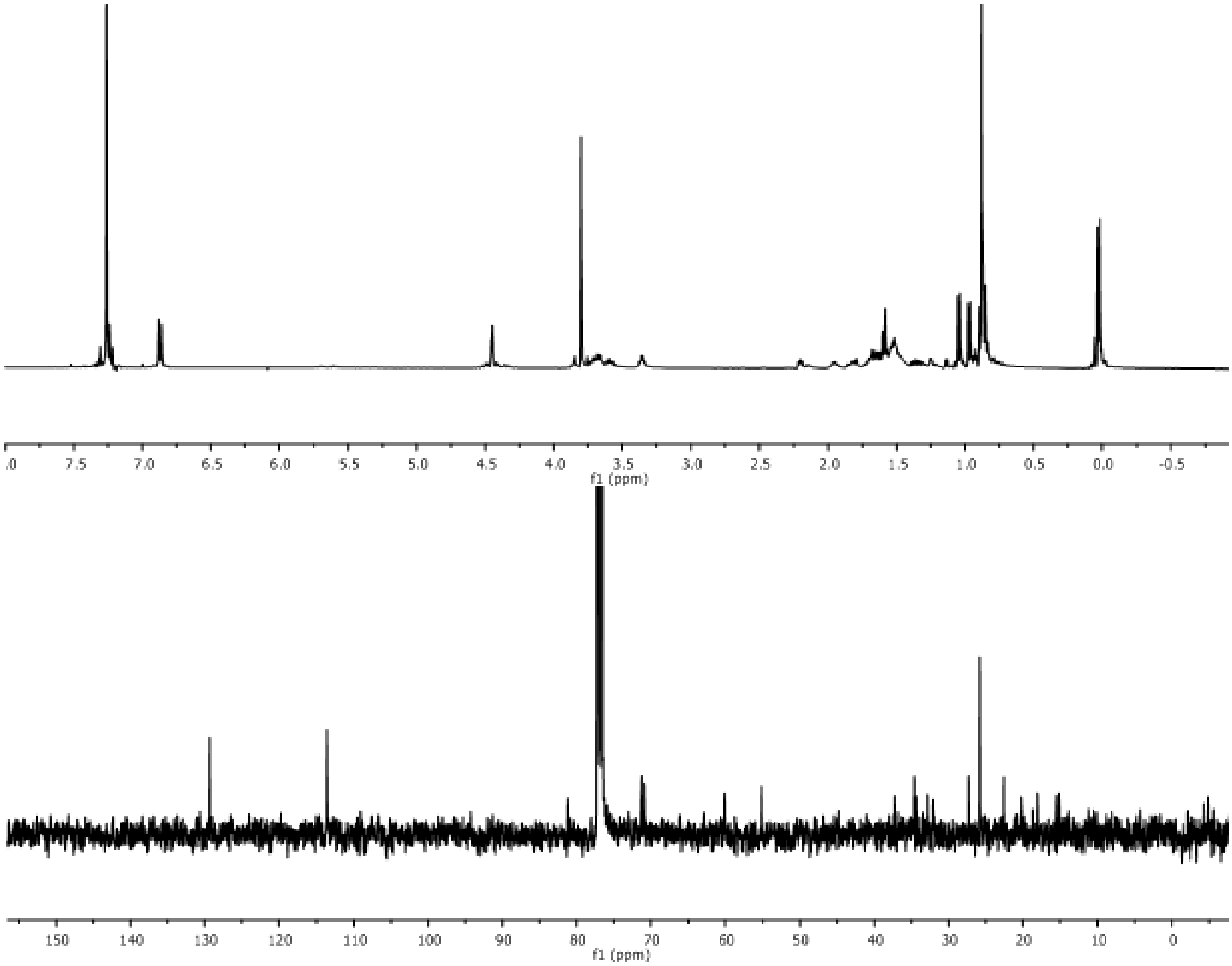

Figure S12 ^{1}H NMR (400 MHz, $CDCl_3$) and ^{13}C NMR (100 MHz, $CDCl_3$) of **18**

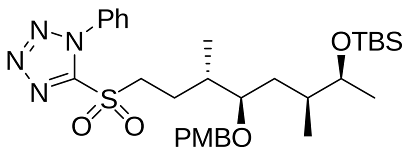

4

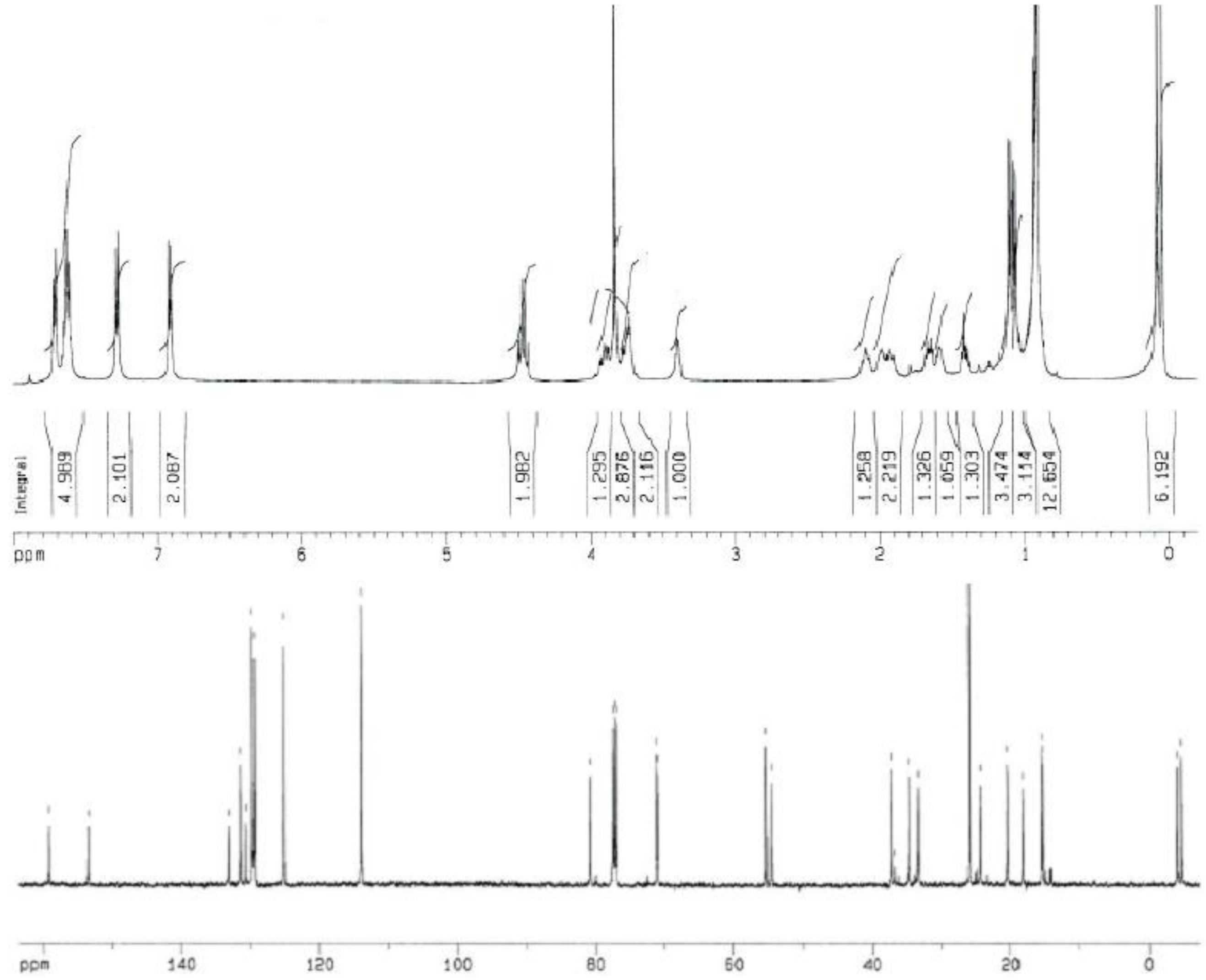

Figure S13 ^1H NMR (500 MHz, $CDCl_3$) and ^{13}C NMR (125 MHz, $CDCl_3$) of **4**

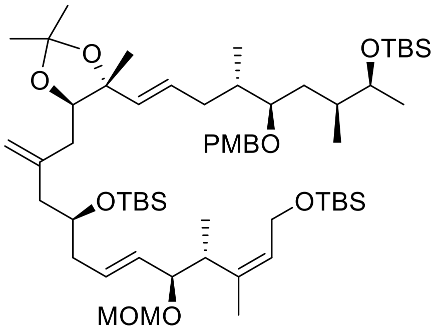

24

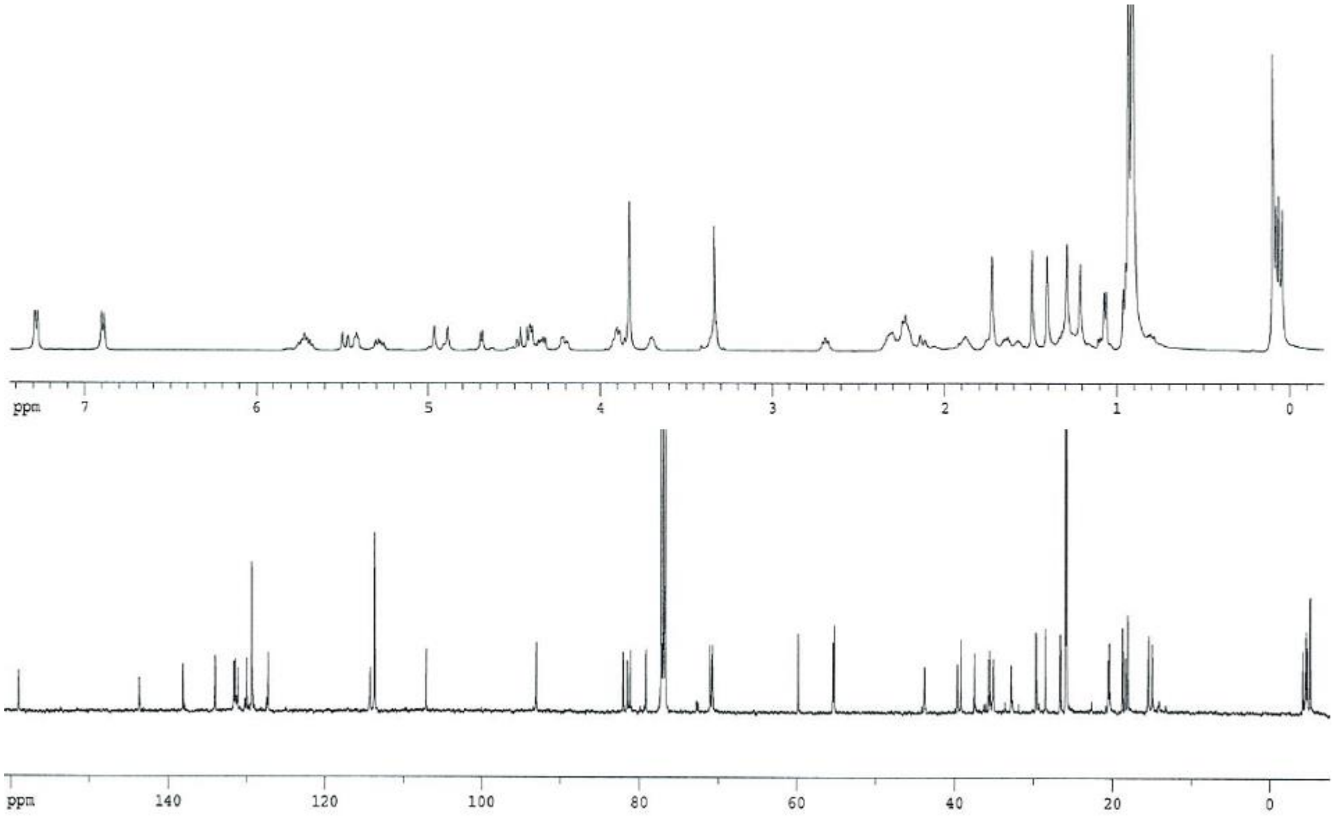

Figure S14 ^{1}H NMR (500 MHz, $CDCl_3$) and ^{13}C NMR (125 MHz, $CDCl_3$) of **24**

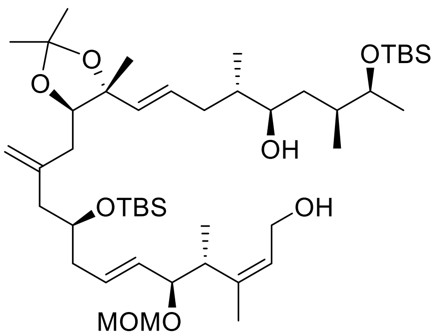

25

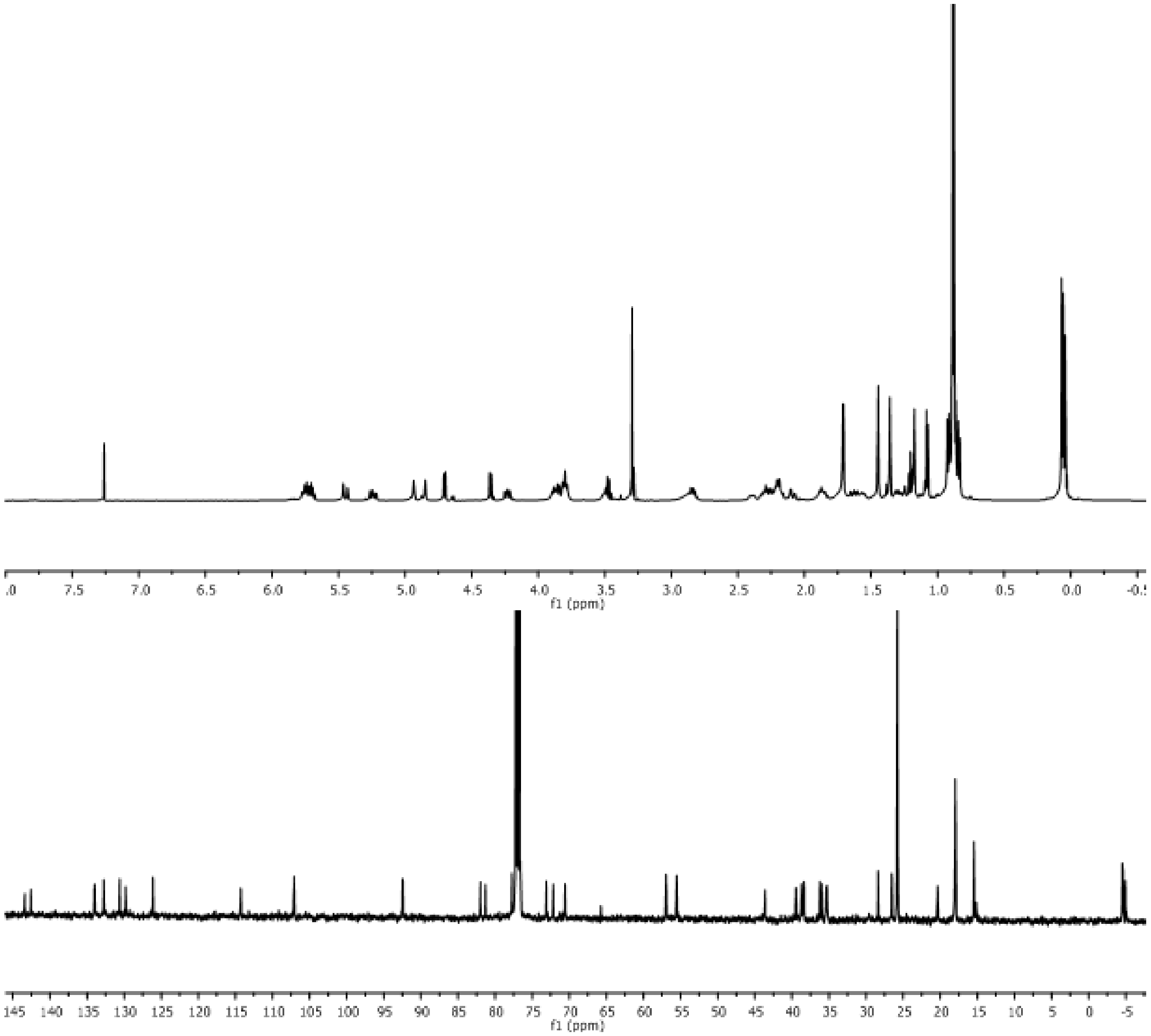

Figure S15 ^{1}H NMR (500 MHz, $CDCl_3$) and ^{13}C NMR (125 MHz, $CDCl_3$) of **25**

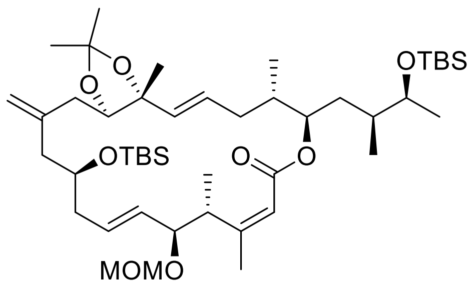

26

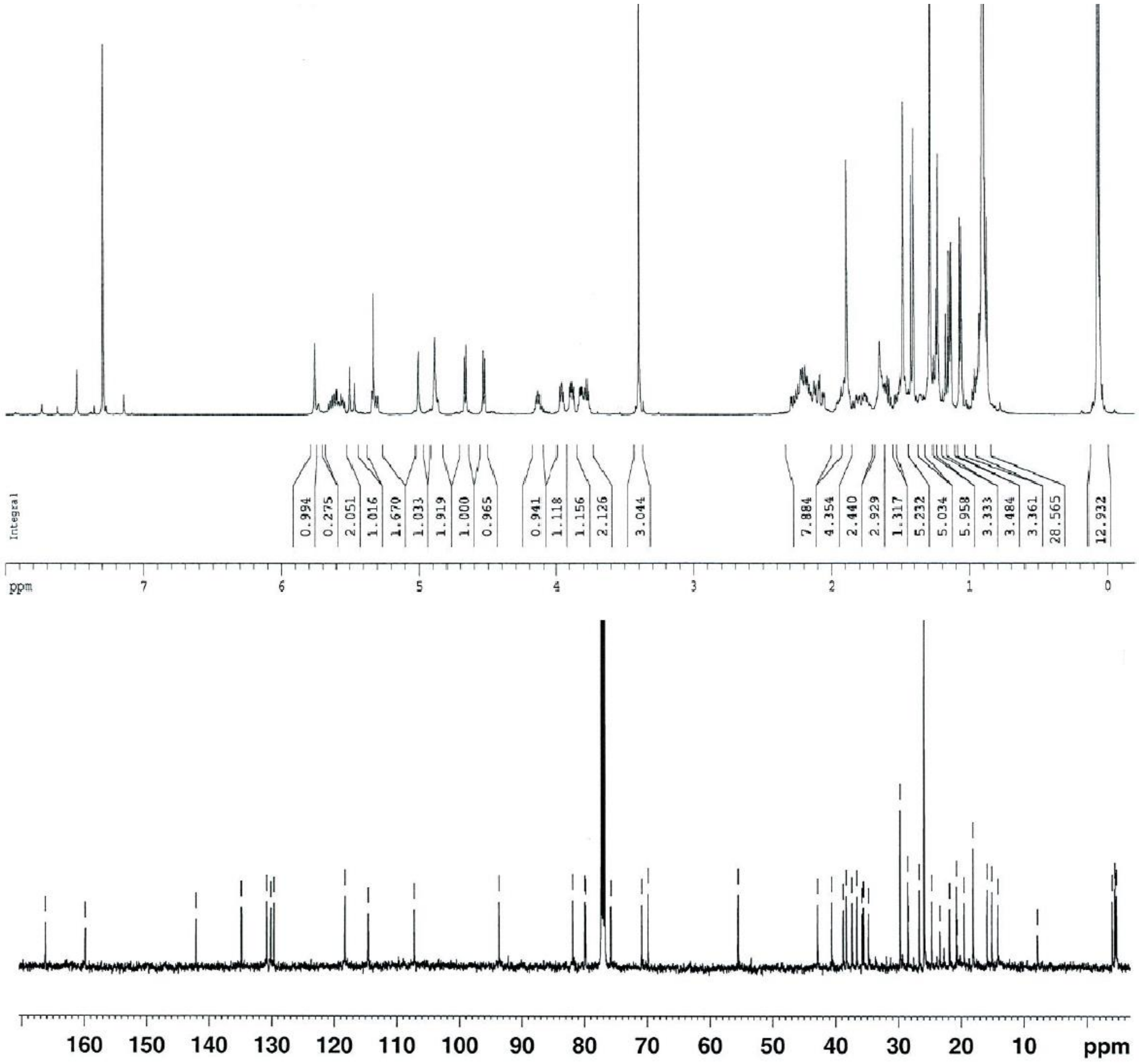

Figure S16 ^{1}H NMR (500 MHz, $CDCl_3$) and ^{13}C NMR (125 MHz, $CDCl_3$) of **26**

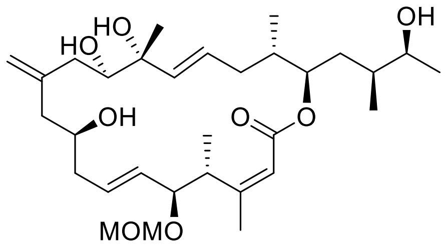

27

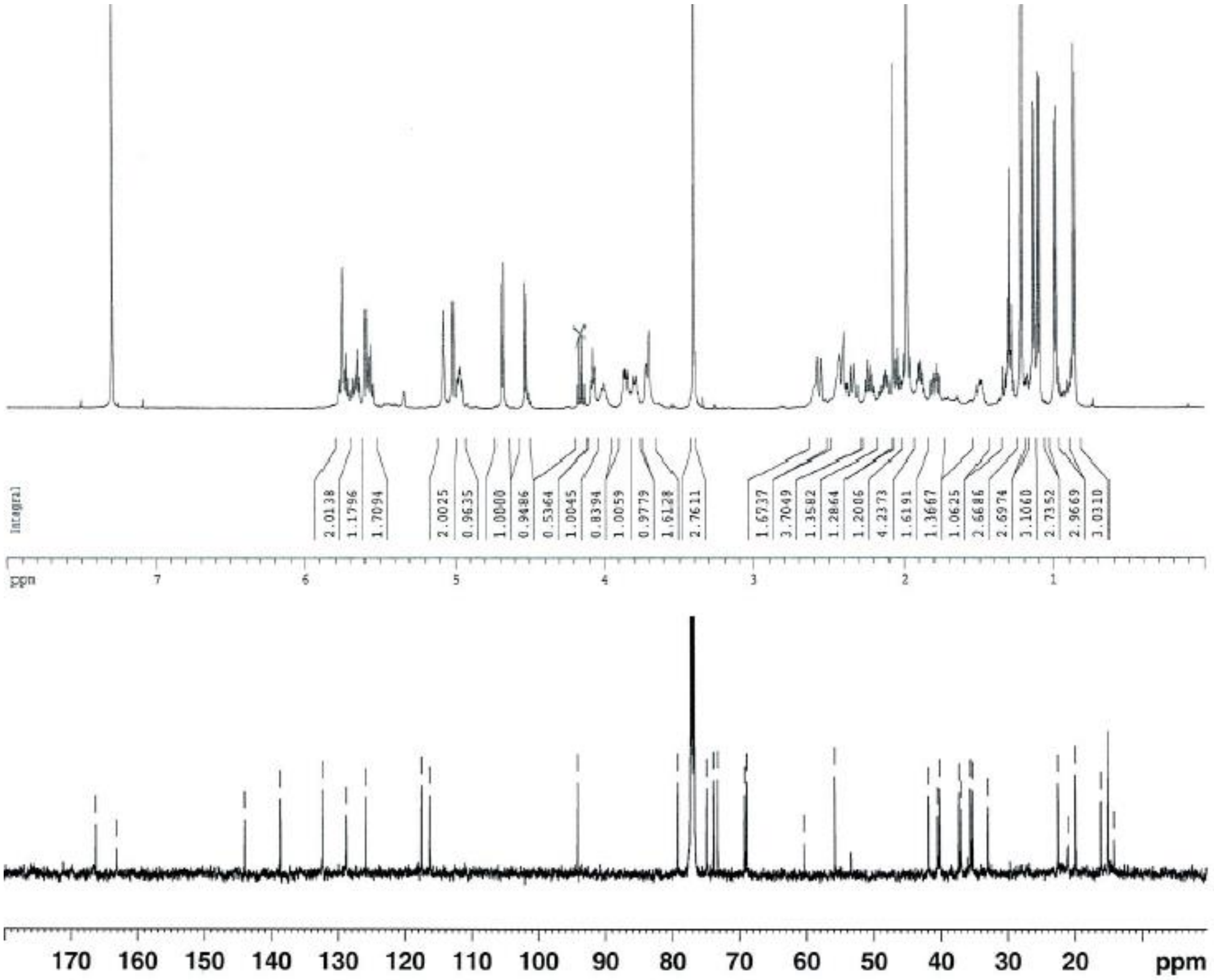

Figure S17 ^{1}H NMR (500 MHz, $CDCl_3$) and ^{13}C NMR (125 MHz, $CDCl_3$) of **27**

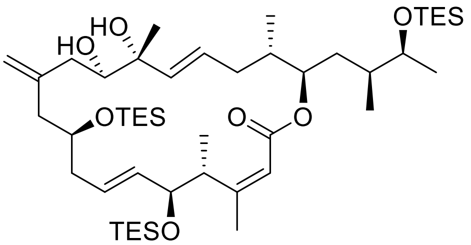

28

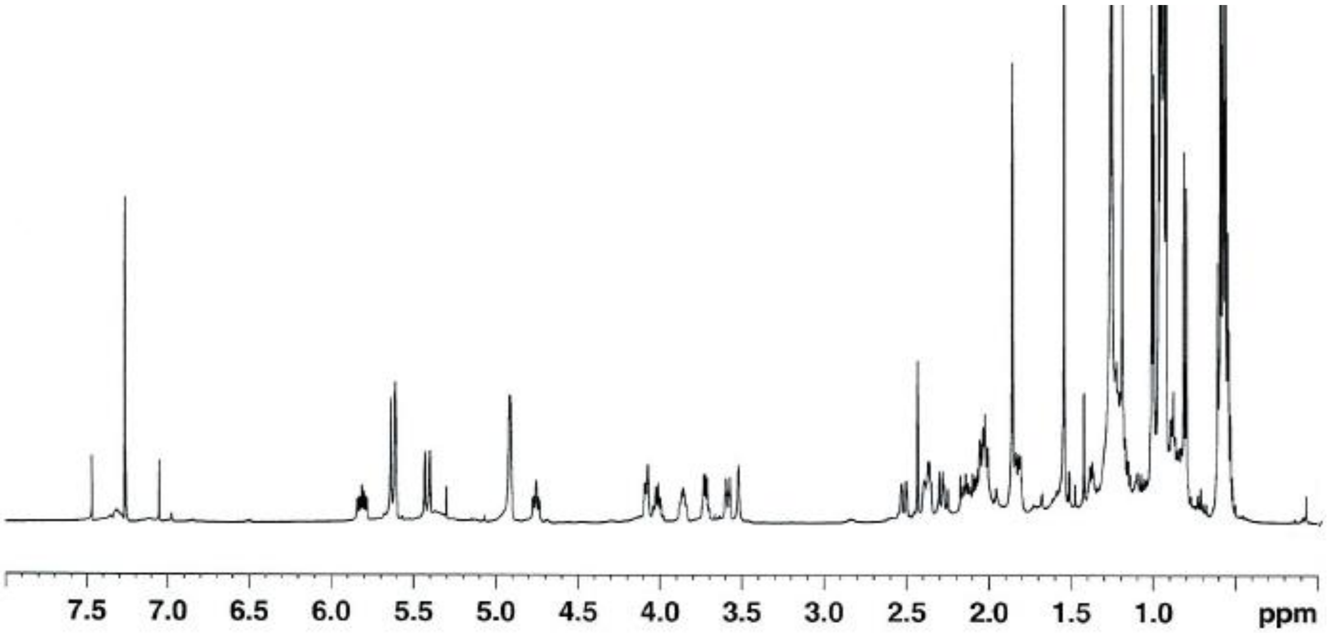

Figure S18 ^{1}H NMR (500 MHz, $CDCl_3$) of **28**

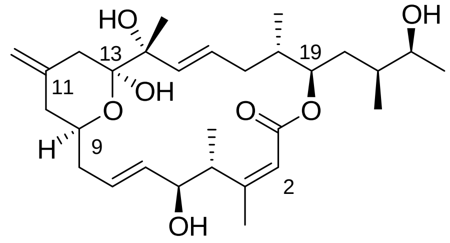

Iriomoteolide-1a (**1**)

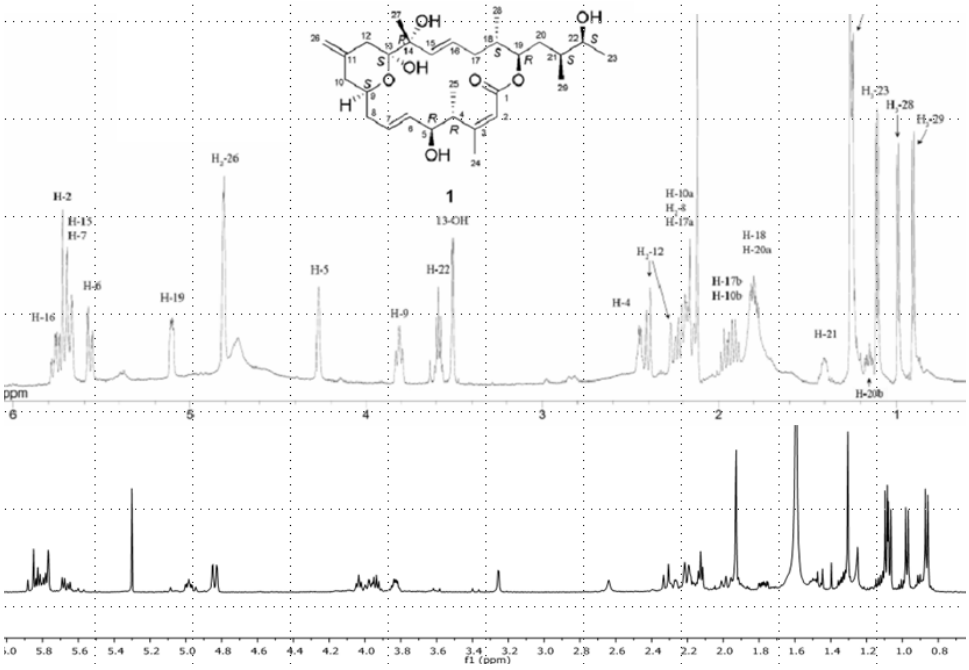

Figure S19 Comparison of ^{1}H NMR (500 MHz, $CDCl_3$) of Natural Iriomoteolide-1a (**1**) (Top) and Synthetic Iriomoteolide-1a (**1**) (Bottom)

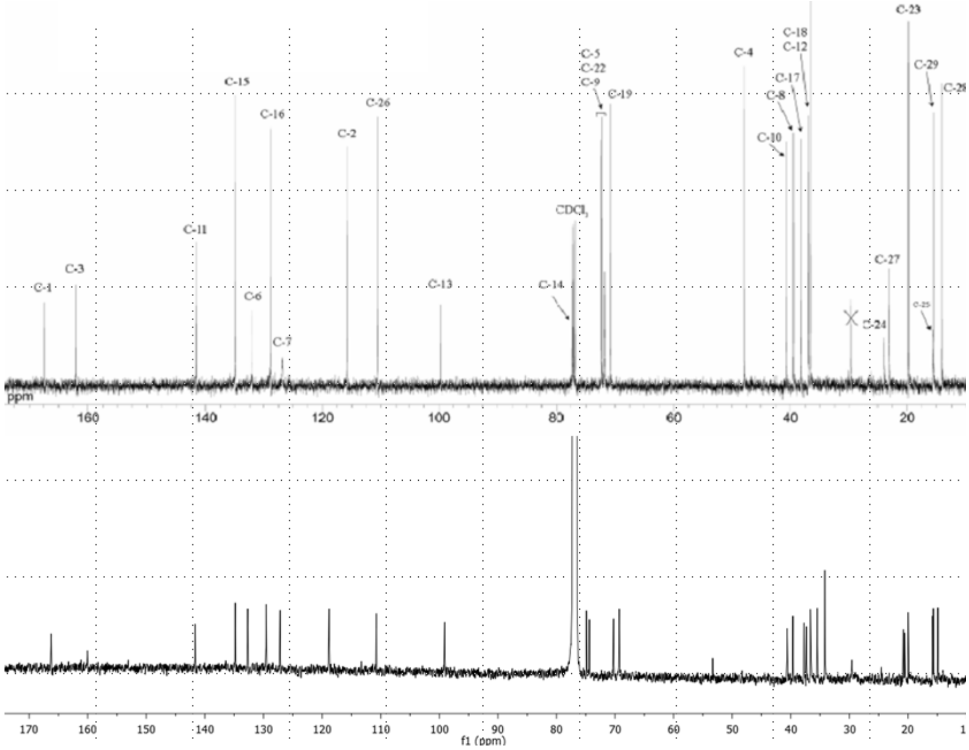

Figure S20 Comparison of ^{13}C NMR (125 MHz, $CDCl_3$) of Natural Iriomoteolide-1a (**1**) (Top) and Synthetic Iriomoteolide-1a (**1**) (Bottom)

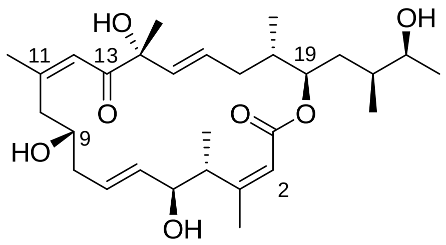

Iriomoteolide-1b (**2**)

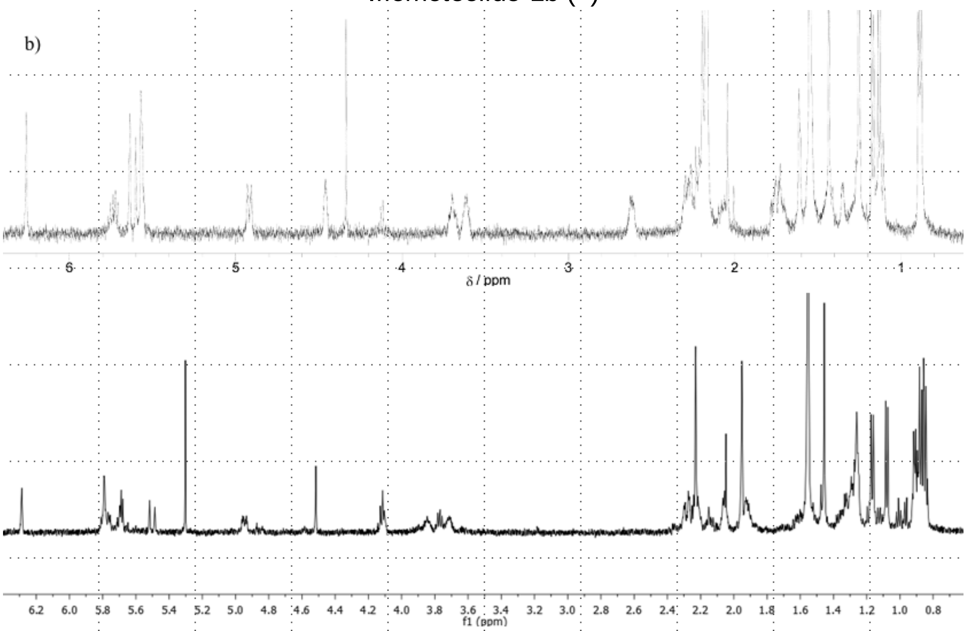

Figure S21 Comparison of ^{1}H NMR (500 MHz, $CDCl_3$) of Natural Iriomoteolide-1b (**2**) (Top) and Synthetic Iriomoteolide-1b (**2**) (Bottom)

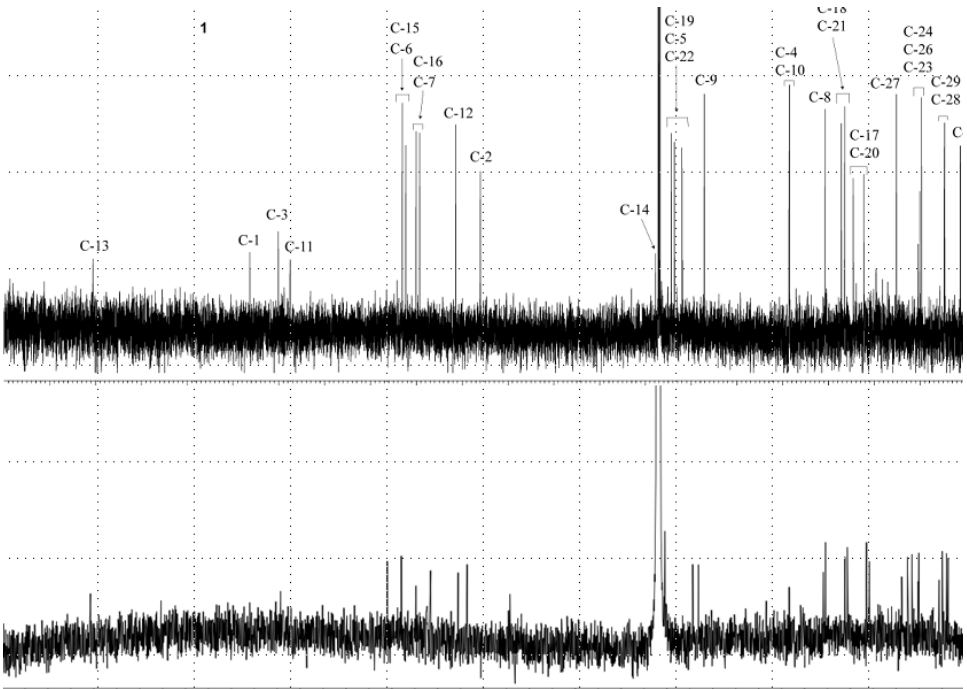

Figure S22 Comparison of ^{13}C NMR (125 MHz, $CDCl_3$) of Natural Iriomoteolide-1b (**2**) (Top) and Synthetic Iriomoteolide-1b (**2**) (Bottom)

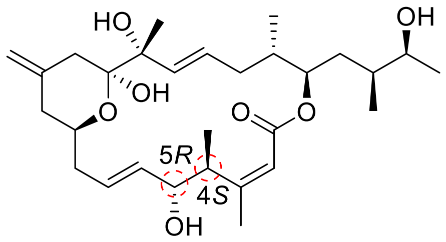

31 (4*S*, 5*R*)

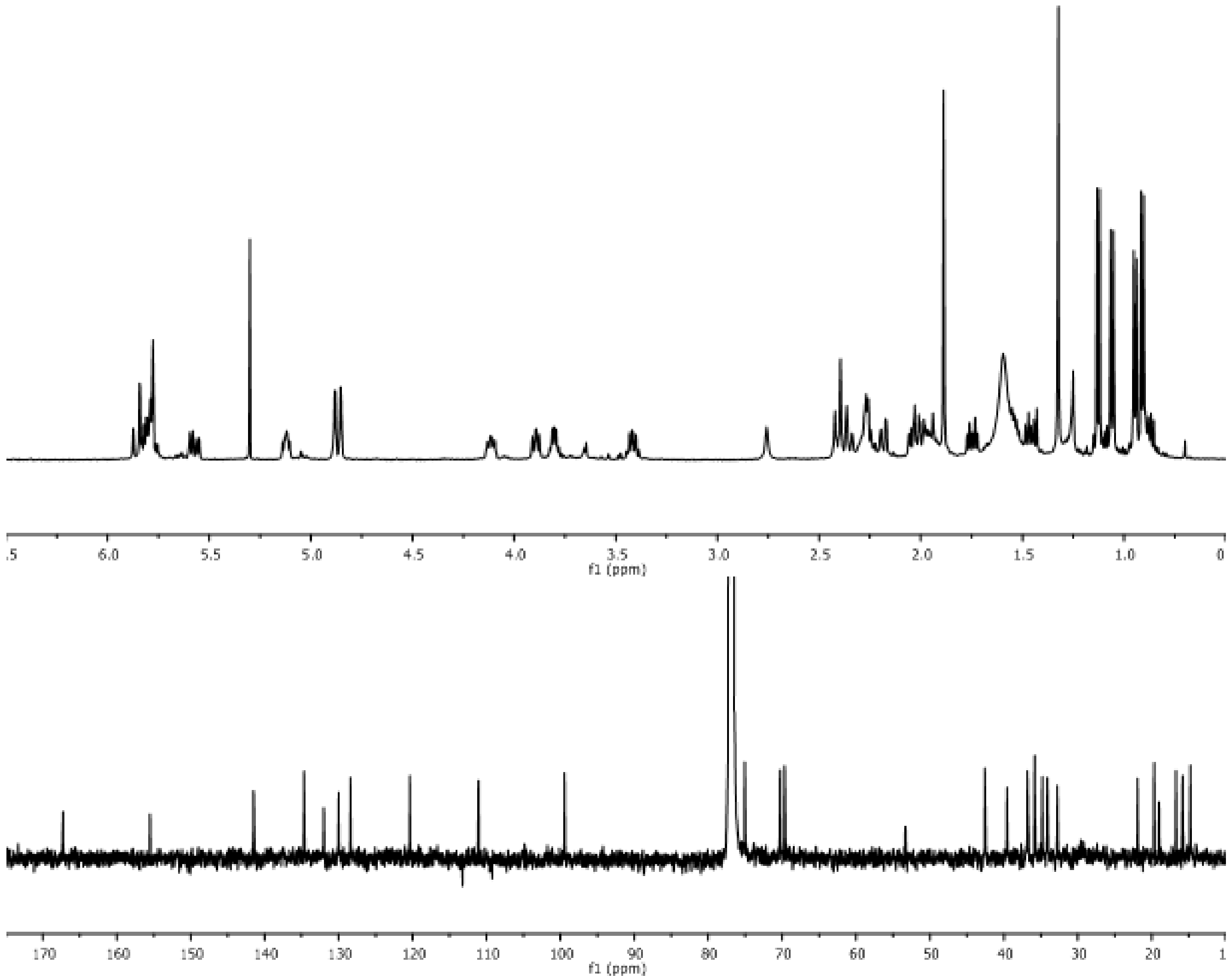

Figure S23 ^{1}H NMR (500 MHz, $CDCl_3$) and ^{13}C NMR (125 MHz, $CDCl_3$) of diastereomer **31**

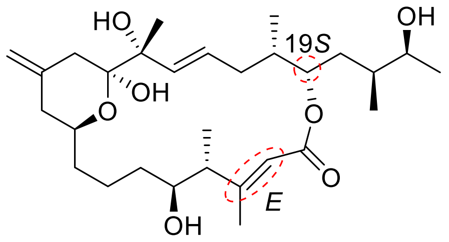

29 (2*E*, 19*S*)

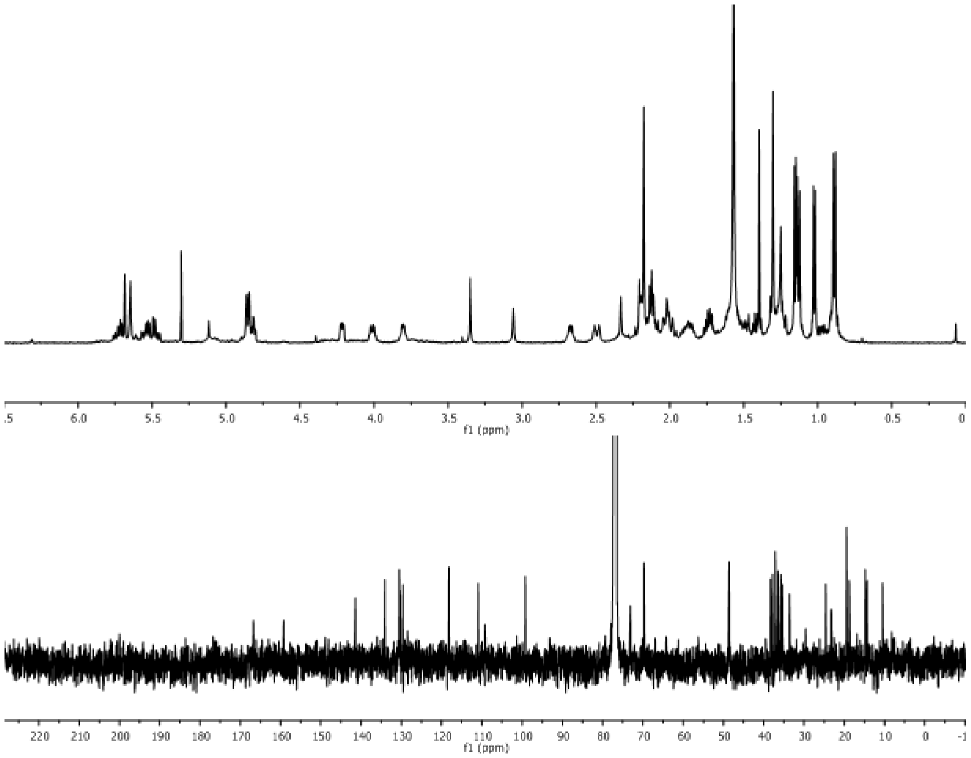

Figure S24 ^{1}H NMR (500 MHz, $CDCl_3$) and ^{13}C NMR (125 MHz, $CDCl_3$) of diastereomer **29**

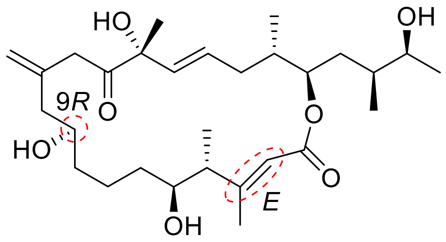

30 (2*E*, 9*R*)

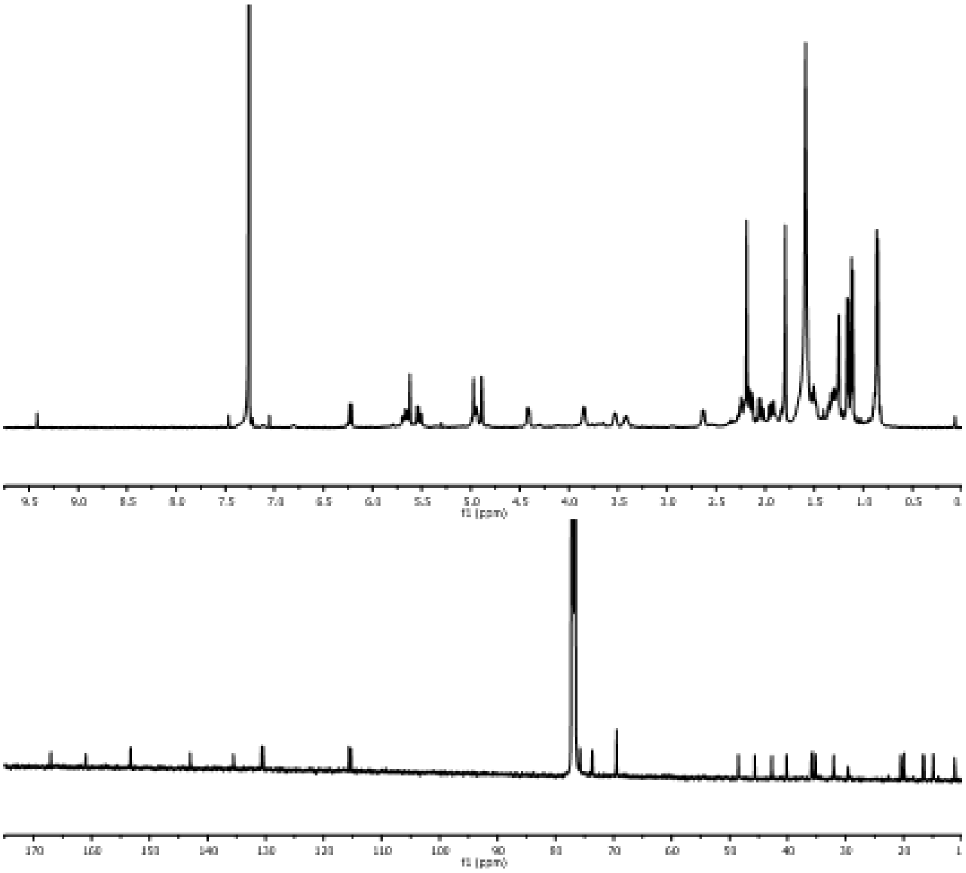

Figure S25 ^{1}H NMR (500 MHz, $CDCl_3$) and ^{13}C NMR (125 MHz, $CDCl_3$) of diastereomer **30**
